# Insight into Radical Initiation, Solvent Effects, and Biphenyl Production in Iron–Bisphosphine Cross-Couplings

**DOI:** 10.1021/acscatal.3c02008

**Published:** 2023-06-22

**Authors:** Maria Camila Aguilera, Achyut Ranjan Gogoi, Wes Lee, Lei Liu, William W. Brennessel, Osvaldo Gutierrez, Michael L. Neidig

**Affiliations:** † Department of Chemistry, University of Rochester, Rochester, New York 14627, United States; ‡ Department of Chemistry, Texas A&M University, College Station, Texas 77843, United States; § Department of Chemistry and Biochemistry, University of Maryland, College Park, Maryland 20742, United States; ∥ Inorganic Chemistry Laboratory, Department of Chemistry, University of Oxford, South Parks Road, Oxford OX1 3QR, U.K.

**Keywords:** iron catalysis, bisphosphine ligands, cross-coupling, mechanism, Mössbauer spectroscopy

## Abstract

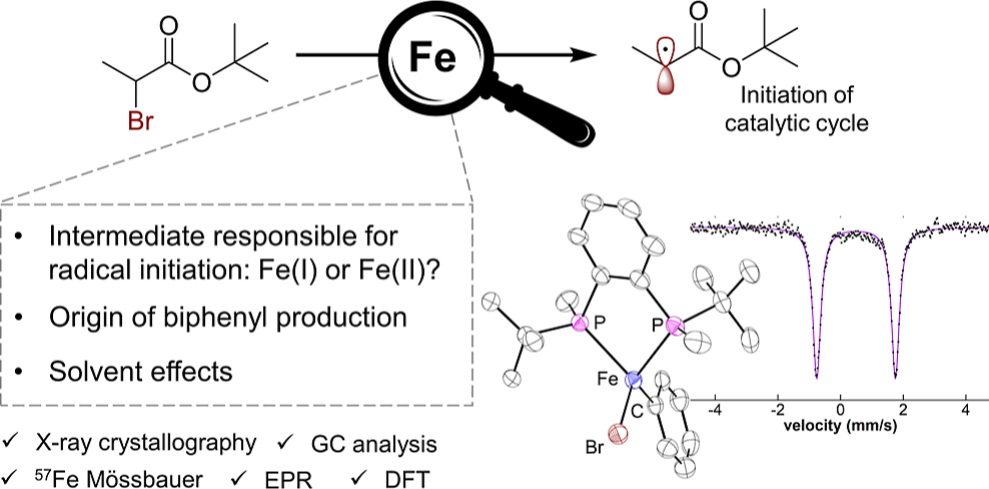

Iron–bisphosphines have attracted broad interest as highly effective and versatile catalytic systems for two- and three-component cross-coupling strategies. While recent mechanistic studies have defined the role of organoiron(II)–bisphosphine species as key intermediates for selective cross-coupled product formation in these systems, mechanistic features that are essential for catalytic performance remain undefined. Specifically, key questions include the following: what is the generality of iron(II) intermediates for radical initiation in cross-couplings? What factors control reactivity toward homocoupled biaryl side-products in these systems? Finally, what are the solvent effects in these reactions that enable high catalytic performance? Herein, we address these key questions by examining the mechanism of enantioselective coupling between α-chloro- and α-bromoalkanoates and aryl Grignard reagents catalyzed by chiral bisphosphine–iron complexes. By employing freeze-trapped ^57^Fe Mössbauer and EPR studies combined with inorganic synthesis, X-ray crystallography, reactivity studies, and quantum mechanical calculations, we define the key in situ iron speciation as well as their catalytic roles. In contrast to iron–SciOPP aryl–alkyl couplings, where monophenylated species were found to be the predominant reactive intermediate or prior proposals of reduced iron species to initiate catalysis, the enantioselective system utilizes an iron(II)-(*R*,*R*)-BenzP* bisphenylated intermediate to initiate the catalytic cycle. A profound consequence of this radical initiation process is that halogen abstraction and subsequent reductive elimination result in considerable amounts of biphenyl side products, limiting the efficiency of this method. Overall, this study offers key insights into the broader role of iron(II)–bisphosphine species for radical initiation, factors contributing to biphenyl side product generation, and protocol effects (solvent, Grignard reagent addition rate) that are critical to minimizing biphenyl generation to obtain more selective cross-coupling methods.

## Introduction

Iron-catalyzed cross-coupling reactions have attracted significant interest in organic synthesis as sustainable and low-cost alternatives to precious metal catalysts commonly used in these transformations.^1−4^ However, iron-catalyzed reactions remain uncompetitive with palladium catalysis for cross-couplings due to challenges including limited reaction methods and substrate scopes. In order to further advance iron cross-couplings, a broader molecular-level understanding of the key in situ-formed iron intermediates, reaction pathways, and mechanisms that enable effective cross-coupling catalysis is critical, yet they have historically remained poorly defined.^2,5−8^ Toward this goal, there has recently been a renewed focus on defining organoiron intermediates as well as ligands, additives, and reaction protocol effects on iron speciation during catalysis that have begun to illuminate the mechanisms of these reactions.^7,9−12^ For example, within the area of cross-couplings with simple ferric salts originally reported by Kochi in the 1970s,^13^ detailed synthetic, spectroscopic, and reaction studies have defined the importance of organoiron clusters such as [MgCl(THF)_5_][Fe_8_Me_12_] in these reactions.^14^ Subsequent studies have also defined the key role of the additive *N*-methyl-2-pyrrolidone (NMP) in accessing three-coordinated homoleptic organoiron complexes that enable productive catalysis across a broader electrophile scope than is accessible with cluster-based reactions.^15^

**Scheme 1:**
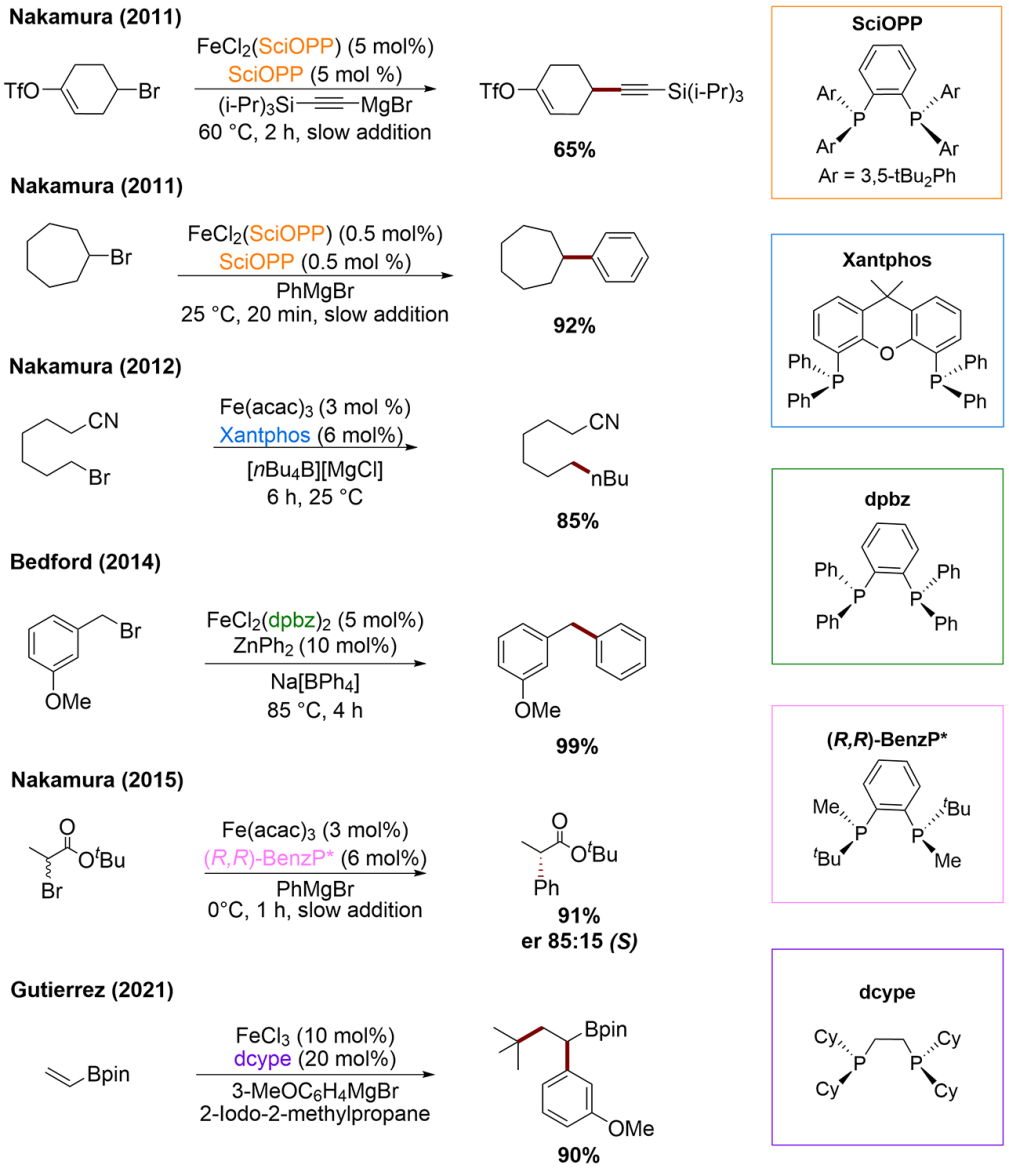
Iron-Catalyzed Cross-Coupling Reactions Supported by Bisphosphine Ligands

Methods employing iron–bisphosphines remain some of the most synthetically useful and versatile strategies for Kumada, Suzuki–Miyaura, and Negishi cross-couplings, employing sp^3^-hybridized coupling partners, leading to C(sp^3^)–C(sp), C(sp^3^)–C(sp^2^) and even challenging C(sp^3^)–C(sp^3^) bond formation in both traditional two-component and, more recently, three-component couplings (Scheme 1).^16−27^ Previous studies by our group have defined the role of transmetalated organoiron–SciOPP bisphosphine (i.e., mono- and bisaryl iron(II) species) (SciOPP = 1,2-(bis[3,5-di(*tert*-butyl)phenyl]phosphino)benzene) as crucial intermediates responsible for initial alkyl radical generation as well as their recombination to achieve selective C–C cross-coupling.^28−30^ This work also identified the formation of an off-cycle, reduced iron(0) [η^6^-biphenyl)(SciOPP)Fe] species, which does not promote productive catalysis. Subsequent mechanistic studies have extended the importance of bisphosphine–iron(II) intermediates for radical initiation and selective product formation in cross-couplings with alkynyl Grignard reagents as well as bisphosphine–iron(II)–aryl intermediates in three-component cross-couplings.^30,31^

Despite these significant advances in our understanding of reactive species and the mechanism of iron-catalyzed cross-couplings using iron–bisphosphines, numerous challenges and mechanistic ambiguities remain that continue to hinder the development of effective cross-coupling systems that can compete with palladium catalysis. For example, (1) how universal is radical initiation by transmetalated iron(II)-bisphosphines in cross-coupling? (2) Can reduced iron–bisphosphine species be effective for radical initiation? (3) What is the origin of biphenyl generation during catalysis that limits overall reaction selectivity? While this has been previously proposed to arise from the over-reduction of iron during catalysis or initial reduction to low-valent iron complexes to initiate catalysis, the prevalence of iron(II) reactive species in many of these cross-coupling protocols leads to ambiguity in the origin of substantial biphenyl generation that can occur. (4) What are the molecular-level effects of variations in solvents that enable selective catalysis? Iron–bisphosphine cross-couplings can be very sensitive to solvent changes (e.g., THF vs diethyl ether or toluene). These questions remained largely unresolved for these systems, yet they are essential to determine in order to facilitate the future development of improved synthetic methods in this area.

**Scheme 2:**
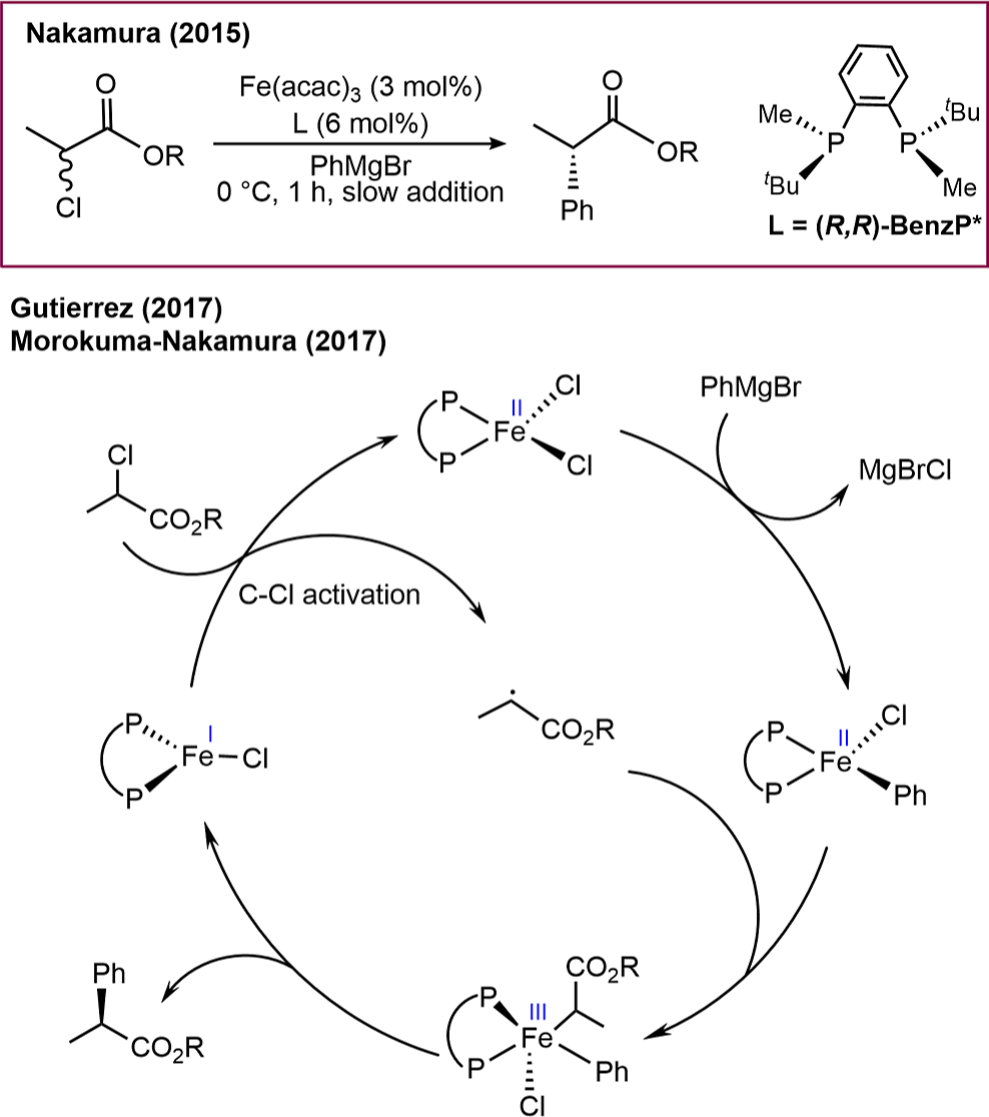
Previously Proposed Mechanism for the Iron-Catalyzed Enantioselective Coupling of α-Chloro- and α-Bromoalkanoates with Aryl Grignard Reagents

The development of asymmetric syntheses catalyzed by iron is an area of current research interest,^32−37^ including the iron–bisphosphine-catalyzed enantioselective coupling of α-chloro- and α-bromoalkanoates with aryl Grignard reagents.^38^ This reaction is successfully performed in the presence of catalytic concentration of an iron salt and the commercially available electron-rich chiral bisphosphine (*R*,*R*)-BenzP* ligand [(*R*,*R*)-(+)-1,2-bis(*t*-butylmethylphosphino)benzene]. Notably, subsequent independent DFT and DFT-AFIR studies by both Gutierrez and Morokuma–Nakamura, respectively, proposed the potential intermediacy of a three-coordinate *S* = 3/2 iron(I)–bisphosphine complex as the key organoiron catalytic species responsible for electrophile activation to initiate catalysis (Scheme 2).^39,40^ This novel mechanistic proposal presented the possibility of an alternative, reduced iron species responsible for radical initiation compared to that previously observed for iron–SciOPP catalysis. While this DFT-derived mechanistic hypothesis could have wide-ranging implications for the rational development of asymmetric iron cross-couplings, there are currently no direct experimental studies that unambiguously characterize the nature of any of the in situ-formed iron species as well as the key, postulated iron(I) intermediate. Therefore, we hypothesized that a detailed mechanistic investigation of this reaction could provide an opportunity to probe both the universality of iron(II) radical initiation in cross-couplings using aryl Grignard reagents and the origins of differences in biphenyl generation across these systems, as well as the effect of experimental protocols on the iron speciation, in order to maximize the catalytic performance.

Herein, we apply a combined synthetic and spectroscopic approach to study the mechanism of iron bisphosphine-catalyzed enantioselective coupling of α-chloro- and α-bromoalkanoates with aryl Grignard reagents. These analyses define the speciation of iron intermediates throughout the catalytic cycle. Combined with further computational studies, this study broadens the universality of iron(II) radical initiation in cross-couplings using aryl Grignard reagents and alkyl electrophiles, defines solvent effects on transmetalation and iron speciation, and provides a new molecular-level framework for understanding the origins of biphenyl generation in catalysis (∼10% for this system) despite predominantly iron(II) speciation in catalysis. We anticipate that this key mechanistic information will accelerate the development of (multicomponent) iron-catalyzed cross-couplings.

## Results

### Iron-(*R*,*R*)-BenzP* Speciation under Catalytically Relevant Conditions

Toward the goal of defining the key iron–bisphosphine species accessible in catalysis (including the species responsible for radical initiation), our initial studies focused on identifying the iron-(*R*,*R*)-BenzP* species that can be formed (and their stabilities) in stoichiometric reactions with phenylmagnesium bromide (PhMgBr) as a representative nucleophile under catalytically relevant conditions (solvent, temperature, and iron concentration). For these reactions, ^57^FeBr_2_ was used as the starting iron salt since it was reported by Nakamura to be equally effective in catalysis as Fe(acac)_3_ and FeCl_2_.^38^ Note that analogous stoichiometric reactions performed using ^57^Fe(acac)_3_ could also access the same iron speciation distribution as ^57^FeBr_2_ following initial reaction to reduce iron(III) to iron(II) (Figure S1).

**Figure 1:**
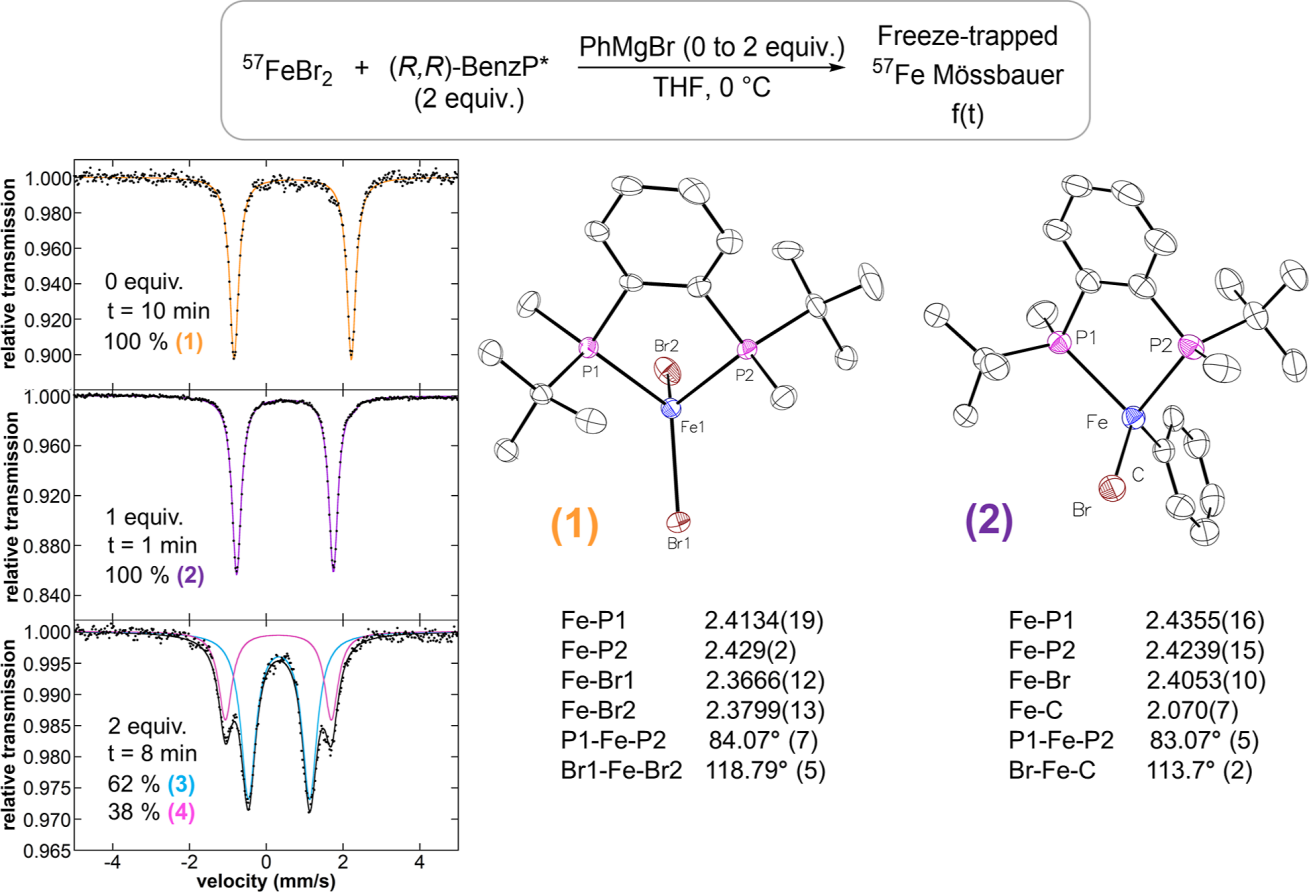
Freeze-trapped 80 K ^57^Fe Mössbauer spectra of stoichiometric reactions. Combining SC-XRD and Mössbauer studies of the crystalline material, the individual components were assigned as the (**1**) Fe(BenzP*)Br_2_ orange component, (**2**) Fe(BenzP*)PhBr purple component, and (**3**) Fe(BenzP*)Ph_2_ and (**4**) Fe(BenzP*)Ph_2_(THF) blue and pink components, respectively. Raw data are shown as black dots, total fit as a black line, and individual components as colored lines. Thermal ellipsoids are shown at 50% probability.

The reaction of ^57^FeBr_2_ with 2 equiv of (*R*,*R*)-BenzP* at 0 °C in THF revealed the formation of a single iron species by freeze-trapped ^57^Fe Mössbauer spectroscopy with Mössbauer parameters of δ = 0.77 mm/s and |Δ*E*_Q_| = 3.00 mm/s (Figure 1). This iron complex was isolated as single crystals by vapor diffusion and characterized by X-ray crystallography as the high spin, distorted tetrahedral dihalide iron(II) species Fe(BenzP*)Br_2_ (**1**) (μ_eff_ = 5.2(2) B.M, as determined by Evans NMR). Note that the chloride analogue of **1** has been previously reported by Nakamura and co-workers.^39^ Reaction of **1** with 1 equiv of PhMgBr in THF at 0 °C resulted in a rapid color change from colorless to pale yellow, with complete conversion to a new iron species as indicated by freeze-trapped 80 K Mössbauer spectroscopy (δ = 0.52 mm/s and |Δ*E*_Q_| = 2.45 mm/s, **2**). The Mössbauer parameters of **2** suggested the formation of a high-spin, monophenylated iron(II) species, consistent with previous reports (Table 1).^28−30^ Highly unstable single crystals were isolated at −80 °C, assigned by X-ray crystallography, and further characterized by Mössbauer spectroscopy as the distorted tetrahedral high-spin Fe(BenzP*)BrPh complex (μ_eff_ = 5.0 (2) B.M, as determined by Evans, δ = 0.49 mm/s and |Δ*E*_Q_| = 2.43 mm/s for isolated material) (Figure 1). It is worth noting that this species is stable in THF, with no evidence of disproportionation even after 1 h at 0 °C. Upon the addition of a second equivalent of PhMgBr, the reaction solution immediately turned orange, and the formation of two new iron species was confirmed by freeze-trapped Mössbauer spectroscopy with a parameter of δ = 0.33 mm/s, |Δ*E*_Q_| = 1.65 mm/s (**3**, 62% after 8 min), and δ = 0.35 mm/s, |Δ*E*_Q_| = 2.85 mm/s (**4**, 38% after 8 min), respectively. The observed parameters of **3** and **4** can be assigned as tetrahedral bisphenylated iron(II)–bisphosphine complexes, consistent with previous reports (Table 1).^28,29^ Performing the addition of 2 equiv of PhMgBr at 0 °C in a non-coordinating solvent (i.e., diethyl ether or toluene), species **4** was not observed despite the formation of an abundance of **3**, consistent with previous studies and further supporting the assignment of **4** as the THF adduct complex of the tetrahedral **3** species (Figure S2).^28^

**Table 1:** Mössbauer Parameters of In Situ-Formed Iron Species Compared to Previously Reported Iron–Bisphosphine Intermediates^a^

species	geometry	isomer shift δ (mm/s)	quadrupole splitting |Δ*E*_Q_| (mm/s)
Fe(BenzP*)Br_2_ this work	dist. *T*_d_	0.77	3.00
Fe(SciOPP)Br_2_^28^	dist. *T*_d_	0.94	2.80
Fe(dcype)Br_2_^30^	dist. *T*_d_	0.72	3.21
Fe(BenzP*)BrPh this work	dist. *T*_d_	0.52	2.45
Fe(SciOPP)BrPh^28^	dist. *T*_d_	0.51	2.35
Fe(SciOPP)BrMes^29^	dist. *T*_d_	0.52	1.97
Fe(dcype)Br(3-MeOC_6_H_4_)^30^	dist. *T*_d_	0.51	2.49
Fe(BenzP*)Ph_2_ this work	dist. *T*_d_	0.33	1.65
Fe(SciOPP)Ph_2_^28^	dist. *T*_d_	0.32	1.50
Fe(SciOPP)(Mes)_2_^29^	sq. planar	0.28	3.67
Fe(dcype)(3-MeOC_6_H_4_)_2_^30^	sq. planar	0.23	4.35
Fe(BenzP*)(THF)Ph_2_ this work		0.35	2.85
Fe(SciOPP)(THF)Ph_2_^28^		0.33	3.13

a dcype = 1,2-bis(dicyclohexylphosphino)ethane. SciOPP = 1,2-(bis[3,5-di(*tert*-butyl)phenyl]phosphino)benzene.

No additional iron species were observed to form upon further reaction up to 1 h at 0 °C and no EPR active species were observed. These observations contrast the prior DFT studies, which suggested the rapid reduction of such bis-arylated iron(II)–bisphosphine complexes (as previously reported).^39^ This experimental observation highlights the relatively high kinetic stability of **3** and **4** against reduction at 0 °C compared to the time of the catalytic reaction (1 h, Figure S3). Consistent with these observations, calculations show that the barrier to form an iron(0)–biphenyl complex directly from **3** is prohibitively high in energy and thermodynamically unfavorable (i.e., barrier is >32 and ∼3.6 kcal/mol uphill). The resistance to undergo reduction is attributed to the energy cost of distorting the high-spin, tetrahedral iron(II) to the corresponding square planar-like geometry in addition to an increase in severe steric interactions between the aryl rings and the alkyl substituents in the transition state structure (see Figure S10 in the Supporting Information). Taken together, this information provides strong support against biaryl formation (and the concomitant formation of iron(0)) directly from reduction of the bisaryl chiral bisphosphine iron(II) under catalysis. Critically, no experimental evidence was found in any of these stoichiometric reaction studies for the formation of the three-coordinate reduced iron(I) *S* = 3/2 species proposed by previous DFT studies as the key active intermediate formed spontaneously under catalytic conditions. Finally, the addition of further equivalents of PhMgBr (3 and 4 equiv in total) at 0 °C in THF also led to the formation of only **3** and **4** in situ.

### Identification of the Iron Intermediate Responsible for Radical Initiation and Selective Product Formation

Having identified the iron-(*R*,*R*)-BenzP* species that can be formed upon reaction with PhMgBr under catalytically relevant conditions, we next proceeded to evaluate their reactivities toward electrophiles (e.g., *tert*-butyl 2-bromopropionate) with the goal of identifying the iron species responsible for radical generation to initiate catalysis. For these studies, enantioselectivity analysis will be disregarded since it has already been comprehensively studied,^39,40^ and we will focus on tracking only product formation (% yield). We employed pseudo-single turnover studies in the presence of excess electrophile to evaluate the reactivities for each complex. While **1** is unreactive with *tert*-butyl 2-bromopropionate, the reaction of **2** (generated in situ) with 33 equiv of *tert*-butyl 2-bromopropionate (the same ratio and concentrations of iron/electrophile present in catalysis) in THF at 0 °C resulted in the formation of **1** (as determined via freeze-trapped Mössbauer) with concomitant, selective formation of a cross-coupled product at an observed rate of ∼0.012 min^–1^ (Figures S4 and S5, and Table S1). However, this reaction occurs far too slowly to be catalytically relevant, as the average turnover frequency during catalysis is nearly 50-fold faster (∼0.56 min^–1^) than the observed rate of reaction of **2**. Thus, these results are inconsistent, with monophenylated species **2** being the iron species responsible for initial radical generation in catalysis.

**Figure 2:**
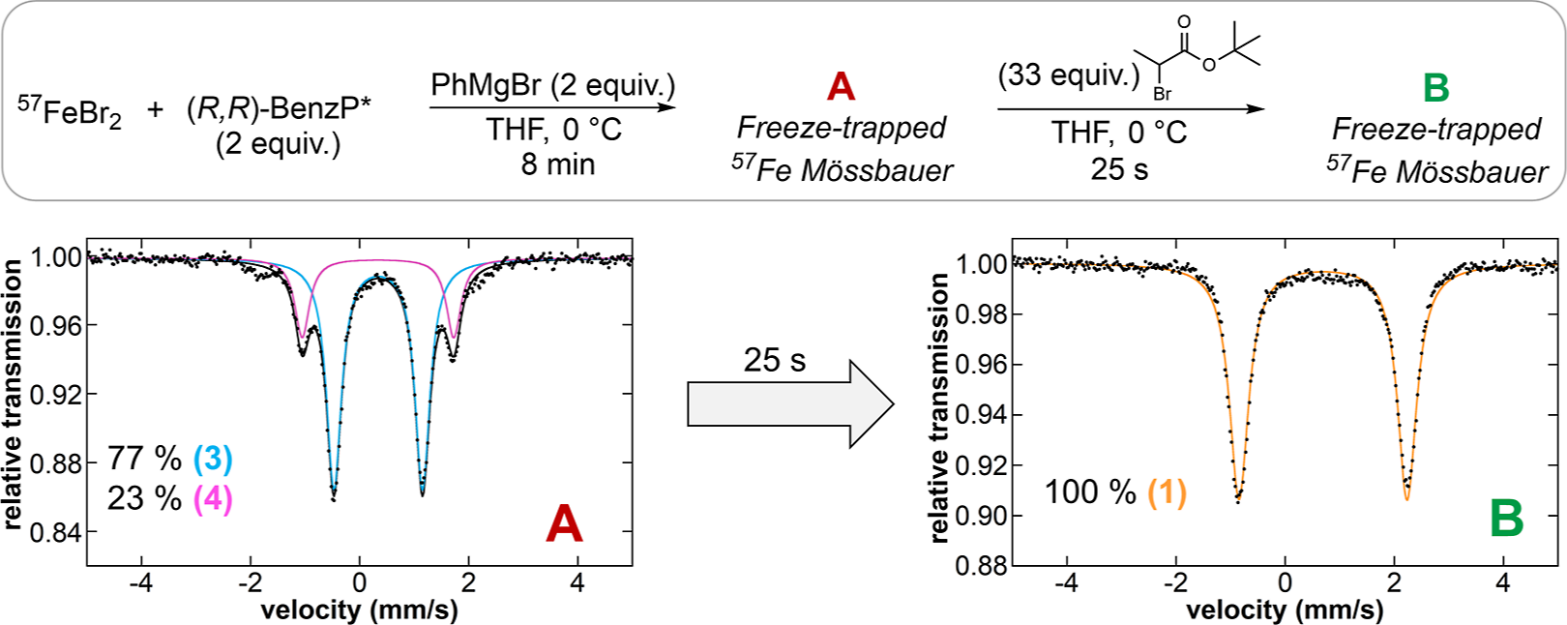
Freeze-trapped 80 K Mössbauer spectra of the in situ formed iron species upon reaction of ^57^FeBr_2_ and 2 equiv of (*R*,*R*)-BenzP*, with 2 equiv of PhMgBr for 8 min (A) and following subsequent reaction with *tert*-butyl 2-bromopropionate for 25 s (B). Raw data are shown as black dots, total fit as a black line, and individual components as colored lines.

We proceeded to evaluate the reactivity of the bisphenylated species **3** and **4** with electrophiles. In contrast to monophenyl iron **2**, these species were found to be highly reactive (*k*_obs_ > 12 min^–1^) upon the addition of 33 equiv of *tert*-butyl 2-bromopropionate in THF at 0 °C, as indicated by an immediate color change from dark orange to colorless after the addition of electrophile (Figure 2). The distinct reactivity for radical formation from mono-aryl and bis-aryl iron species is further corroborated with dispersion-corrected DFT (vide infra; Figures S7 and S8). It is worth noting that Mössbauer spectroscopy showed complete consumption of **3** and **4** and the generation of a single iron species, corresponding to the dihalide species **1**, suggesting that more than one turnover has occurred. Therefore, the fast reactivity observed for **3** and **4** defines these bisphenylated complexes iron(II) as the key active intermediate responsible for the initial radical formation with the electrophile, which is required as the first step in the catalytic cycle. However, when the organic product distribution of this reaction (Figure 2) was analyzed, only 30% yield of the desired cross-coupled product was observed while the rest of the electrophile was consumed, forming side products such as *tert*-butyl acrylate and the dehalogenated product *tert*-butylpropionate. In addition, a significant amount of biphenyl was observed by GC analysis (∼0.8 equiv with respect to iron). To avoid ambiguity about the generation of biphenyl from quenching itself (as stated in previous reports^6,28^), we also performed an NMR analysis of this reaction without the addition of any quenching agent (Figure S6). In this analysis, the formation of biphenyl was also observed upon the reaction of bisphenylated species **3** and **4** with *tert*-butyl 2-bromopropionate, indicating that biphenyl is certainly a result of this reaction (complementary discussion and DFT results vide infra).

These observations led us to propose that the presence of both mono- and bisphenylated species is required to perform catalysis with high yield/selectivity and fast kinetics within relevant time frames, such as the ones reported by Nakamura and co-workers.^38^ Following this hypothesis, we designed an experiment where the monophenylated **2** was generated in situ in the presence of 33 equiv of electrophile (simulating the speciation distribution during catalysis); after 1 min, when we ensured the complete formation of **2**, another equivalent of phenyl magnesium bromide was added dropwise in order to start inducing the formation of **3** and **4** (in low concentration) responsible for the initial radical generation. This experiment sets a scenario in which transmetalation of **2** and subsequent radical formation from either **3** or **4** are in competition with potential radical formation from reaction of **2** with the substrate). Finally, this reaction was quenched after 30 s when GC analysis showed the formation of 90% yield of the desired product. These results imply that since the second transmetalation progresses slowly while radicals are being generated, the remaining high concentration of monophenylated species in solution enables the recombination of these radicals, which is responsible for the selectivity. Thus, while the in situ formation of bisphenylated species **3** and **4** is essential for the initiation of catalysis by radical generation, these results indicate that radical recombination with iron(II) monophenylated **2** is critical to achieve selective C–C bond formation.

**Figure 3:**
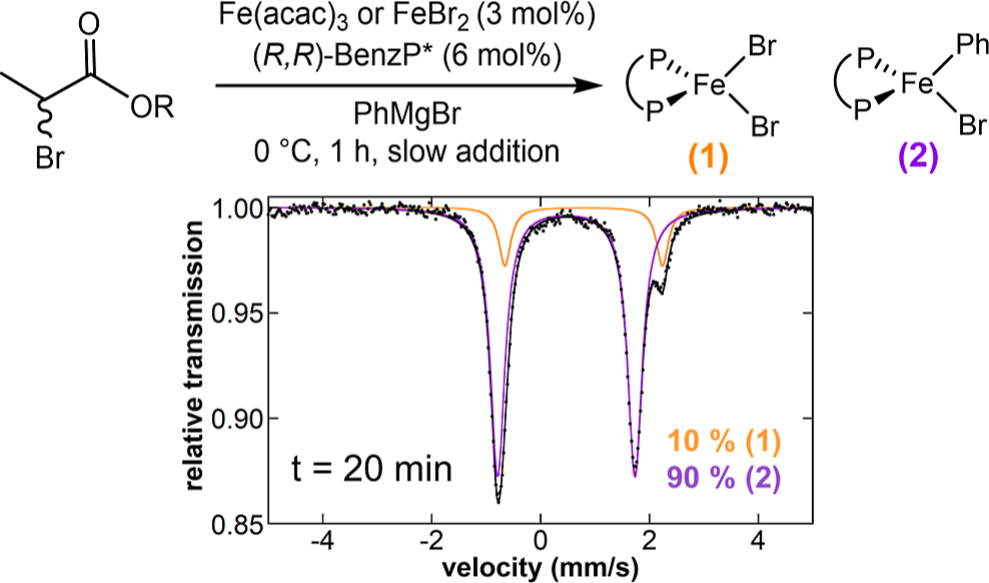
Freeze-trapped 80 K ^57^Fe Mössbauer spectra of the catalytic reaction. Note that the same iron distribution is also observed at 40 min into catalysis. Raw data are shown as black dots, total fit as a black line, and individual components as colored lines.

In order to complement these initial reactivity studies, we also evaluated the in situ speciation distribution at different time points during catalysis using 80 K Mössbauer and 10 K EPR spectroscopy experiments and freeze-trapped samples. Mössbauer spectroscopy showed a high concentration of monophenylated species **2** throughout the catalytic reaction (90% of total iron), while the 10% remaining corresponded to the dihalide species **1**; no EPR active species or bisphenylated iron(II) were observed. The speciation distribution found during catalysis is consistent with the slow reactivity of **2** toward electrophiles and the fast reactivity of **3** and **4** to form alkyl radicals with electrophiles, which are transient intermediates (Figure 3).

Overall, these studies suggest that in the presence of high concentrations of complexes **3** and **4**, the formation of selective cross-coupled products is not favorable. On the other hand, while the kinetics (for radical formation) of the monophenylated species **2** are not catalytically competent, its high concentration during catalysis increases the probability of radical recombination with **2** to enable selective C–C bond formation.

### New Proposal for Biphenyl Side-Product Generation in Iron–Bisphosphine Cross-Coupling

**Figure 4:**
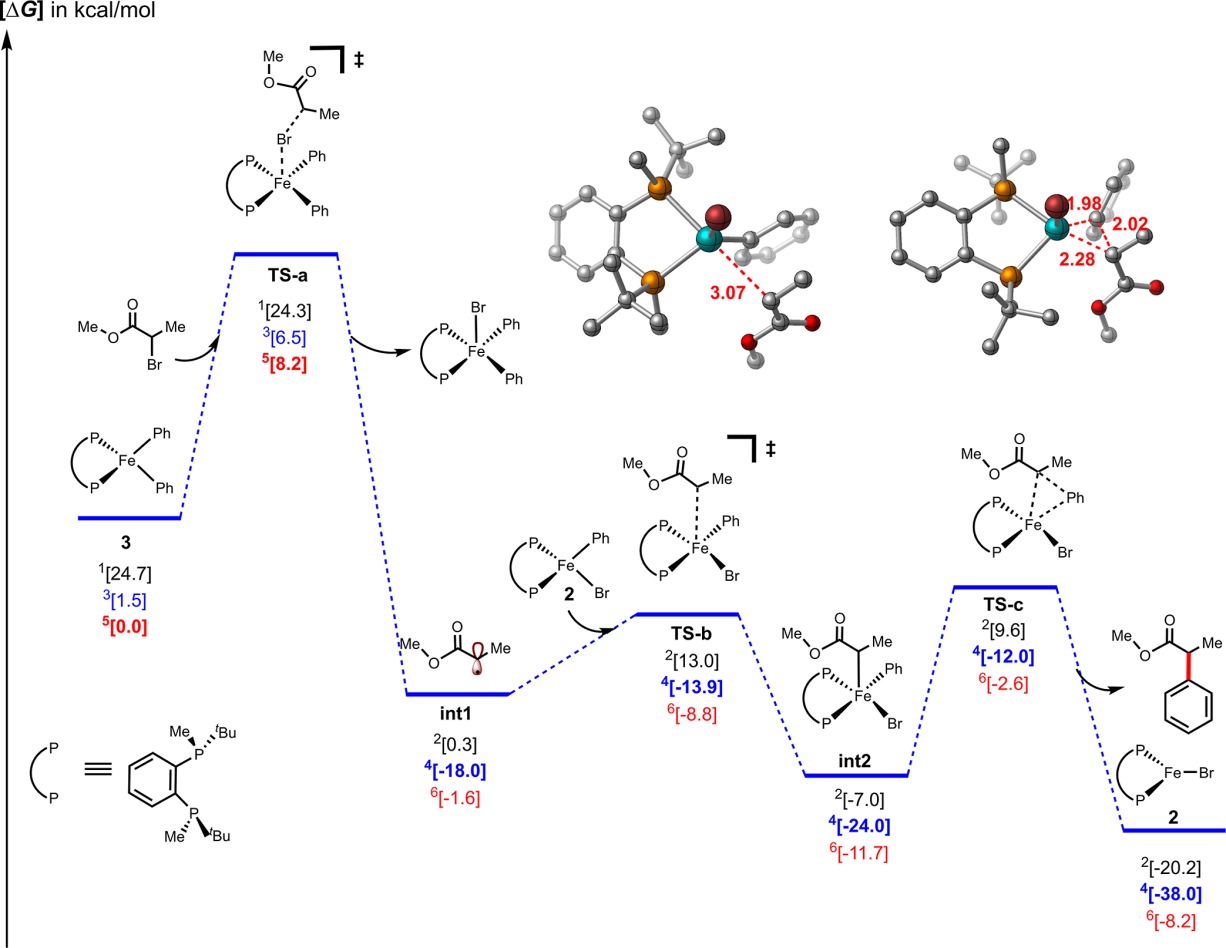
Gibbs free energy (UB3LYP-D3/6-31G(d,p)-THF(SMD); kcal/mol) profile for halogen abstraction by biphenylated iron(II)-bisphosphine complexes and radical recombination by monophenylated iron(II), leading to the formation of the Fe(I)–Br complex along with the desired organic product by reductive elimination. Multiplicities in superscripts.

**Scheme 3:**
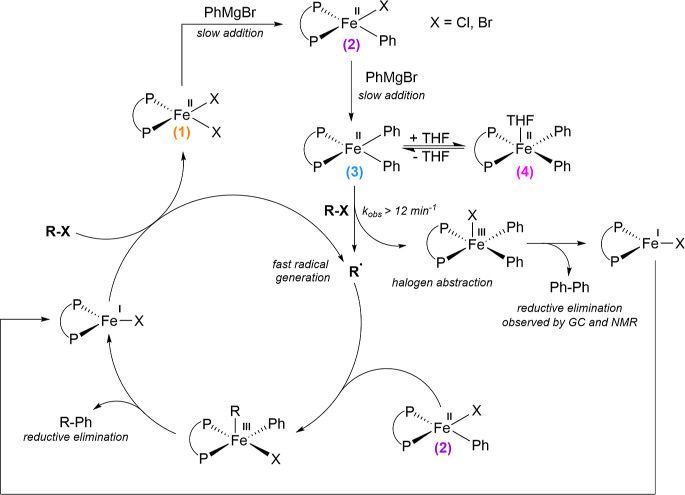
Reaction Pathways and Mechanism for the Enantioselective Iron-Catalyzed Coupling of α-Chloro- and α-Bromoalkanoates with Aryl Grignard Reagents Kinetic rates measured under catalytically relevant conditions: 3 mol % of iron, in THF at 0 °C (as reported in Nakamura’s original synthetic method^38^)

In order to expand our understanding of the reactivity of the bisphenylated species, its second turnover (observed by Mössbauer, Figure 2), and the generation of the biphenyl as a result of its reactivity, we revisited the previously proposed mechanism using dispersion-corrected DFT calculations with full chiral ligands and implicit solvents (THF). As shown in Figure 4, these calculations supported initial radical formation from a bisphenylated iron(II) species via halogen abstraction (barrier of ∼6.5 kcal/mol), resulting in the formation of the alkyl radical and a five-coordinate iron(III). In turn, a potential fate of this five-coordinate iron(III) species is the formation of a tri-coordinate iron(I) and biphenyl through direct reductive elimination (barrier of 8.5 kcal/mol and downhill in energy by 17.0 kcal/mol, as shown in Figure S7). Finally, this putative iron(I) species can rapidly react with the electrophile, enabling the formation of another radical and the regeneration of a distorted tetrahedral dihalide iron(II) (Figure S9). Meanwhile, as previously determined, the alkyl radical can then undergo spin-selective (via quartet spin state) C–C bond formation via radical addition to monophenyl **2** (barrier of only 4.1 kcal/mol) to form iron(III) species (downhill in energy by 6.0 kcal/mol). Finally, reductive elimination will form the desired product (barrier of only 12.0 kcal/mol from the iron(III)). In the reductive elimination step, which is the enantiodetermining step, the barrier for the formation of the (*S*)-product is 3.4 kcal lower than the barrier for the formation of (*R*)-product (Figure S9). Taken together, these calculations are consistent with the faster reactivity for radical formation from bisaryl iron(II) in comparison to monoaryl iron(II), the second turnover shown by Mössbauer spectroscopy (Figure 2), as well as the high amounts of biphenyl generation enabled by halogen abstraction by the bisphenylated species. Once the radical is formed, selective recombination will ensue with the monophenylated species (due to its high concentration during catalysis observed by Mössbauer, Figure 3) to generate the desired cross-coupled product (Scheme 3).

A major implication of these studies is the origin of the undesired, homocoupled biaryl side product in iron-bisphosphine cross-couplings with aryl nucleophiles. Specifically, whereas the amount of homocoupled biphenyl (or biaryl) formed in these reactions has often been correlated in the literature to the degree of reduction of iron in catalysis, resulting in proposals of iron(I) or iron(0) species as the prevalent oxidation states during catalysis,^16,41^ these studies suggest an alternative correlation for biphenyl production that must be taken into consideration. Specifically, the total amount of biphenyl generation during catalysis can be correlated with how often a radical is generated by the bisphenylated species, as radical generation by iron(I) leads to the formation of dihalide species **1,** which is subsequently transmetalated to regenerate the catalytic cycle by forming species **2** and subsequently **3** and **4** that start a new radical cascade (Scheme 3). This revised view of biphenyl generation that ultimately limits catalytic efficiency and selectivity indicates that in order to minimize biphenyl generation (and maximize product selectivity), radical initiation from mono-phenylated iron(II) species would be required.

In fact, previous mechanistic studies of iron–SciOPP phenylated complexes reported the rapid activation of the C–halogen bond of the electrophile and subsequently selective product formation enabled by the monophenylated Fe(SciOPP)PhBr species without needing a higher transmetalated degree species involved in the catalytic cycle.^28^ In contrast, the nature of the slow reactivity of the monophenylated species **2** in this system forces a different mechanism where the formation of the radicals and their subsequent recombination (to form C–C bond) processes go through different iron species. As a consequence, the overall efficiency of the reaction is affected; yields may be more limited since the radical is more likely to find dead ends, generating side products (i.e., dehalogenated side products), in addition to an increase in the amount of the biaryl homocoupled side product (biphenyl) formed during catalysis, which requires the use of more nucleophile, PhMgBr. Therefore, the nature of the iron(II) species responsible for radical initiation (i.e., mono vs bisphenylated) not only changes the reaction pathway but also affects the overall efficiency of catalysis. Therefore, the study of the structural aspects of bisphosphines that lead to desirable reactive monophenylated complexes (**2**) is an important consideration for future ligand design and the development of more efficient synthetic methods.

### Molecular Effects of Experimental Protocols to Maximize Yields: Slow Nucleophile Addition and Solvent Effects

The rigorous requirements of some experimental protocols, such as slow addition of the nucleophile in order to maximize yields, are directly correlated to how these protocols affect the iron intermediates distribution during catalysis. For example, the control of the concentration of bisphenylated species **3** and **4** is crucial to minimize the generation of biphenyl as well as excess radicals that lead to side products. Therefore, experimental protocols that enable control of the transmetalation step are crucial to maximize yields.

Nakamura reported an increase of 77% product yield when the nucleophile is added slowly over the course of catalysis (91% yield) compared to when added in one portion (14% yield).^38^ This is a consequence of controlling the minimum formation of bisphenylated species **3** and **4** during catalysis, which are highly reactive but not selective intermediates, while favoring a high concentration of monophenylated complex **2** that carries out the C–C bond-forming step selectively, achieving the desired organic product in high yields. Notably, for this specific reaction, the nucleophile addition rate needs to be approximately ∼7 times slower than for other methods that require slow addition such as iron/SciOPP (7 equiv wrt iron/min vs 1.1 equiv wrt iron/min for iron/BenzP*).^20,38^ Thus, more rigorous experimental protocols need to be implemented to achieve desired yields as a consequence of the lack of reactivity of monophenylated species (**2**).

Lastly, iron–bisphosphine cross-coupling reactions often show a remarkable sensitivity to the solvent with regards to catalytic performance and product selectivity. While THF is commonly selected as an optimal solvent during the development of iron-catalyzed cross-coupling methods, the molecular-level effects of variations in the solvent that enable selective catalysis remain largely undefined. In the case of this cross-coupling method, we observed a decrease in the product yield when non-coordinating solvents are employed for catalysis (∼91% yield for THF vs ∼78% yield for toluene), even while the nucleophile is added slowly (0.017 μL/min, i.e., ∼1.1 equiv wrt iron/min). Therefore, we proceeded to evaluate the effect of employing non-coordinating solvents on the iron speciation generated and their reactivity. Surprisingly, we initially observed no effect on the type of iron intermediates formed under catalytically relevant conditions (mono and bisphenylated intermediates were formed) or their reactivity (exact same kinetic rate for the reactivity of bisphenylated species *k*_obs_ > 12 min^–1^). By analyzing the organic product distribution when toluene is employed to perform catalysis, we also observed an increase in the total amount of biphenyl generated (∼20% vs 12% for THF). This observation led us to consider the effect of the solvent on the formation rate of the bisphenylated species (i.e., transmetalation from **2** to **3**).

By performing stoichiometric reactions under catalytic conditions employing THF, we observed the full conversion from **2** to a mixture of **3** and **4** within 8 min (monophenylated to bisphenylated species). In contrast, by using only non-coordinating solvents, such as toluene, the monophenylated intermediate (**2**) is transmetalated much faster, yielding the formation of **3** within only 2 min (we do not observe the formation of **4** since it corresponds to a THF adduct species as aforementioned). This observed accelerated transmetalation process is correlated to the decrease of product yield along with an increase of the amount of biphenyl produced as a consequence of the higher concentrations of **3** and **4** in situ in solution available for radical formation. Overall, these observations highlight the importance of experimental protocols that enable controlling the transmetalation degree of the iron speciation to achieve high yields. We are currently exploring these effects of controlling the transmetalation step as they relate to efficiency in stereoselective multicomponent cross-couplings and will report in due course.

## Conclusions

In this study, the iron intermediates and the mechanism of the enantioselective coupling of α-chloro- and α-bromoalkanoates with aryl Grignard reagents have been defined through the use of a combination of spectroscopic methods, kinetic studies, and low temperature synthesis complemented by DFT calculations. In contrast to the previous proposal based on DFT studies, we observed only the initial formation of phenylated iron(II) intermediates (Fe(BenzP*)BrPh (**2**) and Fe(BenzP*)Ph_2_ (**3**)), a prevalent oxidation state during catalysis. No further reduced iron species were observed to form at a catalytically relevant time. These observations are consistent with previous reports for other iron/bisphosphine catalytic systems, where iron(II) intermediates have a relevant role for the initiation of the catalytic cycle. However, this study emphasizes that the nature of the iron(II) intermediate (mono vs bisphenylated complexes) responsible for radical generation to start catalysis has a direct effect on the reaction pathway, which is also reflected in the catalytic efficiency. In contrast to the phenylated iron–SciOPP system, we found that the monophenylated species iron-BenzP* (**2**) is less reactive toward electrophiles, leading to the requirement of forming a more reactive intermediate with a higher transmetalation degree (bisphenylated) for radical formation. Our experimental observations as well as complementary DFT results indicated the generation of biphenyl as a side product as a consequence of the radical generation process by the bisphenylated intermediate through halogen abstraction, yielding a decrease in the overall catalytic efficiency compared to systems that enable the formation of reactive monophenylated intermediates. In addition, we defined the effect of experimental protocols such as slow nucleophile addition and optimal solvent on controlling the transmetalation step, which is crucial to maximize product yield by minimizing the formation of the bis-phenylated complexes **3** and **4** that would lead to biphenyl generation upon reaction with the electrophile. Overall, by elucidating the reaction pathway operated for the enantioselective coupling of α-chloro- and α-bromoalkanoates with aryl Grignard reagents catalyzed by iron-(*R*,*R*)-BenzP*, we address significant experimental aspects related to the reactivity of iron/bisphosphine catalytic systems, providing insights for the development of more efficient cross-coupling strategies catalyzed by iron.

## Supporting Information Available

Experimental procedures, including Mössbauer spectroscopy, EPR spectroscopy, NMR spectroscopy, and synthetic procedures, and characterization data, including Mössbauer spectra, EPR spectra, NMR spectra, and DFT data (PDF)
Crystallographic data for Fe(BenzP*)Br_2_ (**1**) and Fe(BenzP*)BrPh (**2**) (CIF)
checkCIF/PLATON report for structure (**2**)(PDF)
checkCIF/PLATON report for complex (**1**) (PDF)

## Supplementary Material

cs3c02008_si_001.pdf

cs3c02008_si_002.cif
