# Supplementary material for: Insight into Radical Initiation, Solvent Effects, and Biphenyl Production in Iron–Bisphosphine Cross-Couplings
Source: ACS Catal. 2023 Jun 22;13(13):8987–96. doi: 10.1021/acscatal.3c02008 (PMC10334425; doi:10.1021/acscatal.3c02008)
Supplement: Supplementary file 1 — cs3c02008_si_001.pdf [file cs3c02008_si_001.pdf]

Supporting Information for

Insight into Radical Initiation, Solvent Effects and Biphenyl Production in Iron-Bisphosphine Cross-Couplings

Maria Camila Aguilera,[1] Achyut Ranjan Gogoi,[2] Wes Lee,[3] Lei Liu,[2] William W. Brennessel,[1] Osvaldo Gutierrez,[2,3*] and Michael L. Neidig[1,4*]

[1]Department of Chemistry, University of Rochester, Rochester, New York 14627, United States.
[2]Department of Chemistry, Texas A&M University, College Station, Texas 77843, United States.
[3]Department of Chemistry and Biochemistry, University of Maryland, College Park, Maryland 20742, United States.
[4]Inorganic Chemistry Laboratory, Department of Chemistry, University of Oxford, South Parks Road, Oxford OX1 3QR, United Kingdom

Table of Contents

1. Experimental Procedures S2
  1.1. Mössbauer Spectroscopy S2
  1.2. Electron Paramagnetic Resonance Spectroscopy (EPR) S2
  1.3. NMR Spectroscopy S3
  1.4. Synthetic Procedures S3

2. Supplementary Data S5
  2.1. ^{57}Fe Mössbauer S5
  2.2. NMR Spectra S9
  2.3. X-Ray Crystallography S10
  2.4. Computational Supporting Information S22

1. Experimental Procedures

1.1. Mössbauer Spectroscopy. All samples were prepared under nitrogen atmosphere in a glovebox equipped with a liquid nitrogen fill port to enable sample freezing at 77 K within the glovebox. Samples were loaded in Derlin Mössbauer sample cups, and subsequently frozen in liquid N_2. Zero field 80 K Mössbauer measurements were performed using a SeeCo. MS4 Mössbauer spectrometer integrated with a Janis SVT-400 T He/N_2 cryostat. Isomer shifts reported were determined relative to α-Fe at 298 K. In addition, reported ΔE_Q parameters for zero field correspond to absolute values. All Mössbauer spectra were fit using the program WMoss (SeeCo). ^{57}Fe metal was purchased from Isoflex (>95% isotope enriched). Freeze trapped solution Mössbauer samples were prepared utilizing $^{57}FeBr_2$. Experiments were carried out under catalytically relevant conditions as reported for the method (iron concentration, temperature, and solvent). Solid Mössbauer samples were prepared by isolating crystalline material obtained from crystallization procedures performed starting from $^{57}FeBr_2$.

1.2. Electron Paramagnetic Resonance Spectroscopy (EPR). Samples for EPR spectroscopy were prepared in an inert atmosphere glove box equipped with a liquid nitrogen fill port to enable sample freezing to 77 K within the glove box. EPR samples were prepared in 4 mm OD suprasil quartz EPR tubes from Wilmad Labglass. Samples for spin integration utilized high precision suprasil quartz tubes to allow for direct comparison of intensities between different samples. X-band EPR spectra were recorded on a Bruker EMXplus spectrometer equipped with a 4119HS cavity and an Oxford ESR-900 helium flow cryostat. The instrumental parameters employed for all samples were as follows: 0.0012 mW power;

time constant 0.01 ms; modulation amplitude 1 G; 9.38 GHz; modulation frequency 100 kHz. A 5 mM $CuSO_4$ standard was used as reference under non-saturating conditions. Identical instrumentation parameters were used for both the iron and standard samples. Reactions were performed at catalytic relevant conditions and subsequently diluted at 5 mM in order to prepare the EPR samples by freeze quenching.

1.3. NMR spectroscopy. ^1H NMR spectrum was recorded at 400 MHz on a Bruker DPX-400 spectrometer, respectively, locked on the signal of deuterated THF solvent. d8-THF was purchased from Cambridge Isotope Laboratories and degassed by freeze−pump−thaw cycles.

1.4. Synthetic Procedures

All synthetic procedures were performed under nitrogen atmosphere in a glovebox.

Preparation of 1 (Fe(BenzP*)Br_2): A 20 mL scintillation vial was charged with 3.3 mg of $FeBr_2$, 8.5 mg of (*R,R*)-BenzP* (2 equiv) and 1 mL of THF. The colorless solution formed was stirred for 30 min at RT. This mixture was transferred to a 4 mL vial and allowed to crystallize by vapor diffusion with dry diethyl ether at RT, resulting in colorless needles of **1** suitable for X-ray crystallography after 1 day.

Preparation of 2 (Fe(BenzP*)BrPh): A 20 mL scintillation vial was charged with 3.5 mg of $FeBr_2$, 9.0 mg of (*R,R*)-BenzP* and 1 mL of THF. The colorless solution formed was stirred for 30 min at RT. This mixture was placed at 0 °C and 1 equiv. of PhMgBr was added dropwise, resulting in a yellow solution. After 5 min of stirring, THF was removed under vacuum at -30 °C. The solid obtained was chilled to -80 °C followed by the addition of 2.5 mL of cold dry diethyl ether. After 30 min at -80 °C, the resulting solution was filtered through celite and 1 mL pentane was added. Solution was stored at -80 °C for 1 week

rendering colorless crystalline needles of **2**. These crystals co-crystallize with 11% of **1**, likely due to disproportionation over time.

2. Supplementary Data

2.1. ^{57}Fe Mössbauer

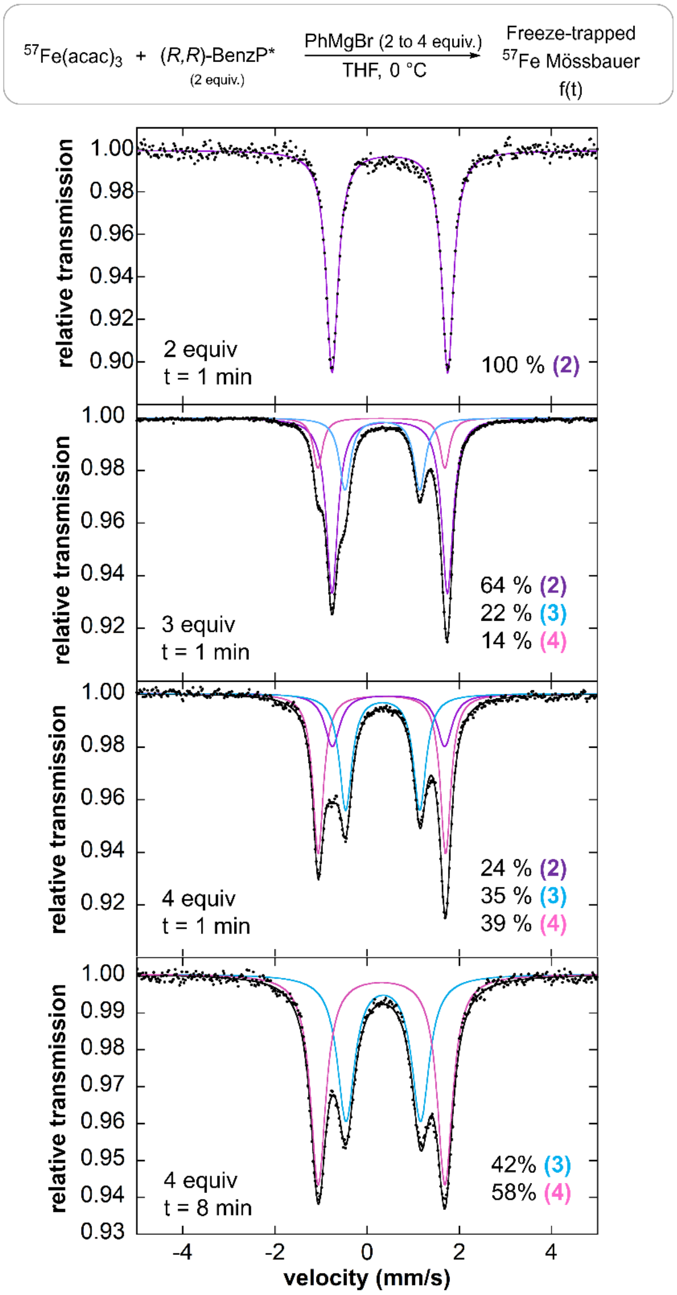

Figure S1. Freeze-trapped 80K ^{57}Fe Mössbauer spectra of stochiometric reactions starting with ^{57}Fe(acac)$_3$. the individual components were assigned as (**2**) Fe(BenzP*)PhBr purple component (**3**) Fe(BenzP*)Ph_2 and (**4**) Fe(BenzP*)Ph_2(THF) to blue and pink components respectively.

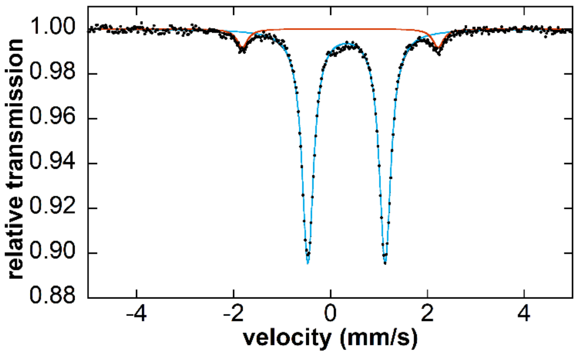

Figure S2. Freeze-trapped 80 K ^{57}Fe Mössbauer spectrum of reaction of $FeBr_2$, 2 equiv of (*R,R*)-BenzP* and 2 equiv of PhMgBr in toluene at 0 °C within 2 min. Blue component (96%) is assigned to (**3**) Fe(BenzP*)Ph_2 (parameters listed in table 1 in the manuscript). Red component has Mössbauer parameters of δ = 0.23 mm/s and $|\Delta E_Q|$ = 4.00 mm/s, it is assigned to the square planar analogue of **3.**

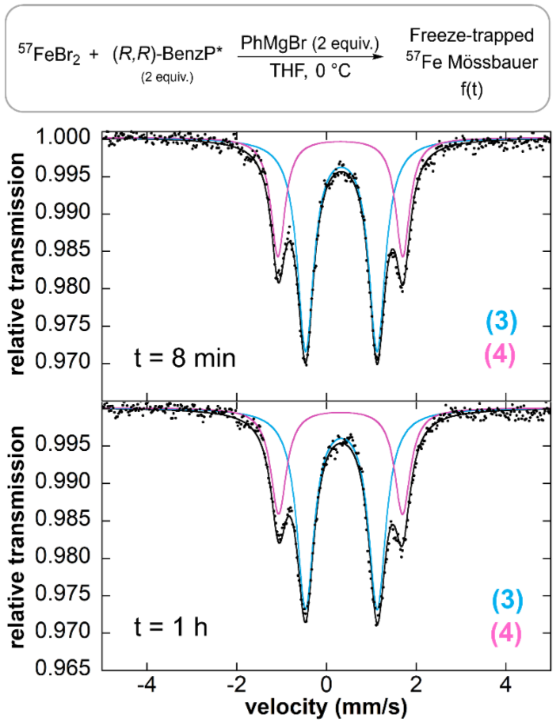

Figure S3. Freeze-trapped ^{57}Fe Mössbauer spectrum of reaction of $FeBr_2$, 2 equiv of (*R,R*)-BenzP* and 2 equiv of PhMgBr in THF at 0 °C at different time points to evaluate the stability of the iron speciation. the individual components were assigned as (**3**) Fe(BenzP*)Ph_2 and (**4**) Fe(BenzP*)Ph_2(THF) to blue and pink components respectively.

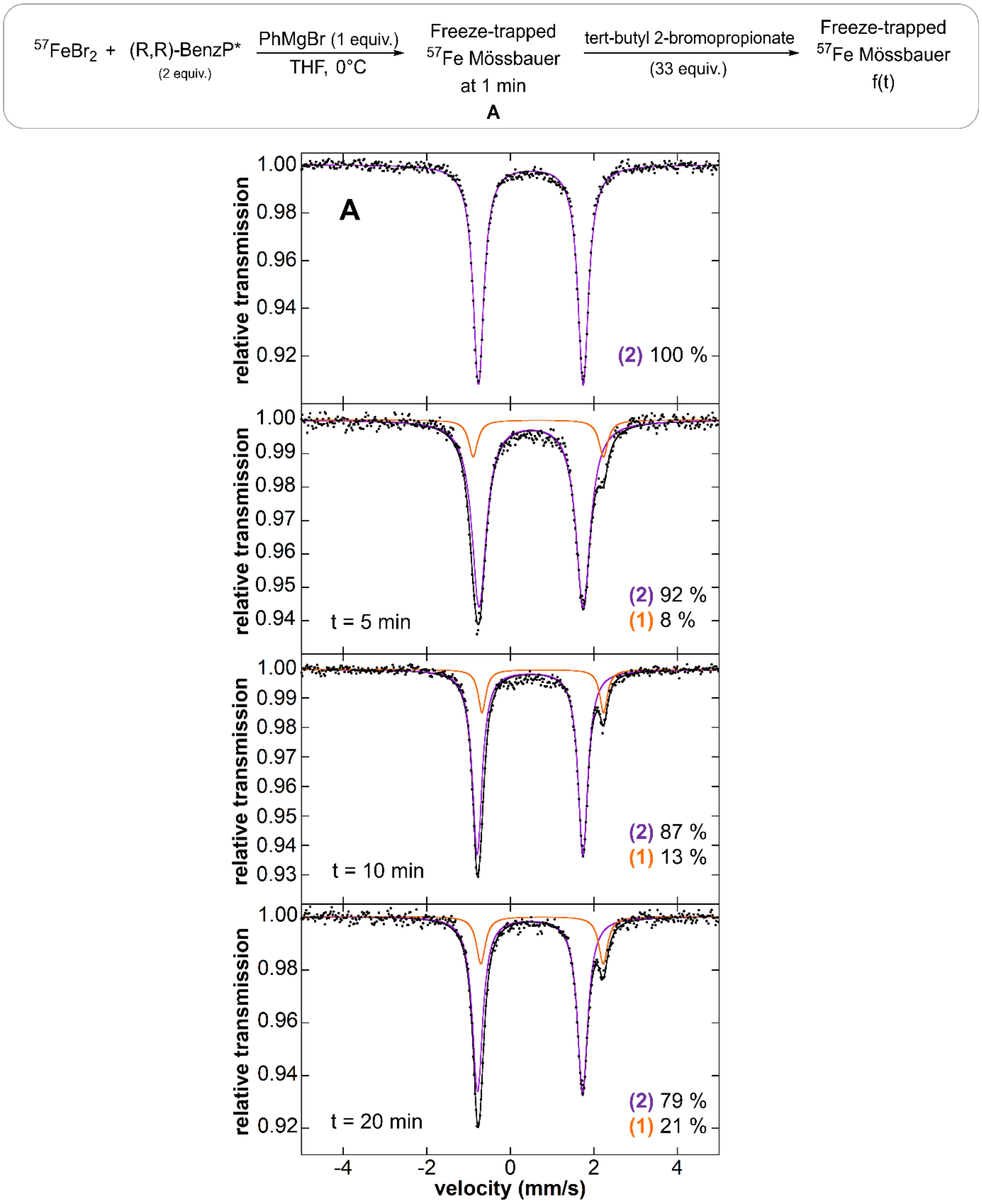

Figure S4. Freeze-trapped 80 K ^{57}Fe Mössbauer spectra of the reaction of **2** (purple) with *tert*-butyl 2-bromopropionate in THF at 0 °C, as a function of reaction time. The top spectrum indicates initial distribution before addition of electrophile. 15 min spectrum was not included; however quantification information is reported in Table S1.

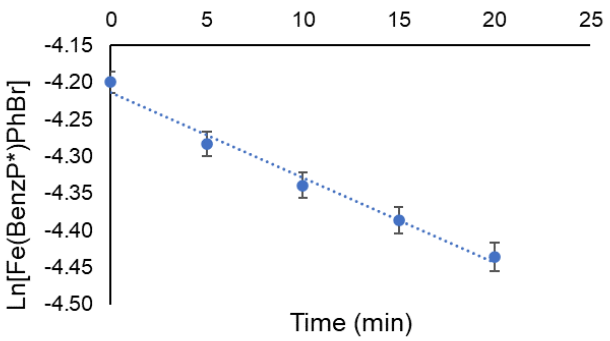

Figure S5. First order kinetic data for the reaction of **2** with *tert*-butyl 2-bromopropionate in THF at 0 °C using freeze-trapped ^{57}Fe Mössbauer spectroscopy measurements. k_{obs} = ~ 0.012 min^{-1} ± 0.002.

Table S1. Results of simultaneous analyses (^{57}Fe Mössbauer and GC) of the reactivity of **2** towards tert-butyl 2-bromopropionate. Organic product analysis can be correlated to the amount of **2** consumed generating **1.**

	**Mössbauer analysis**		**GC analysis**
**Time points**	Fe(BenzP*)PhBr **(2)** (%)	Fe(BenzP*)Br_2 **(1)** (%)	Product yield (%)
**0 min**	100	0	N/A
**5 min**	92	8	9
**10 min**	87	13	14
**15 min**	83	17	17
**20 min**	79	21	19

2.2. NMR Spectra

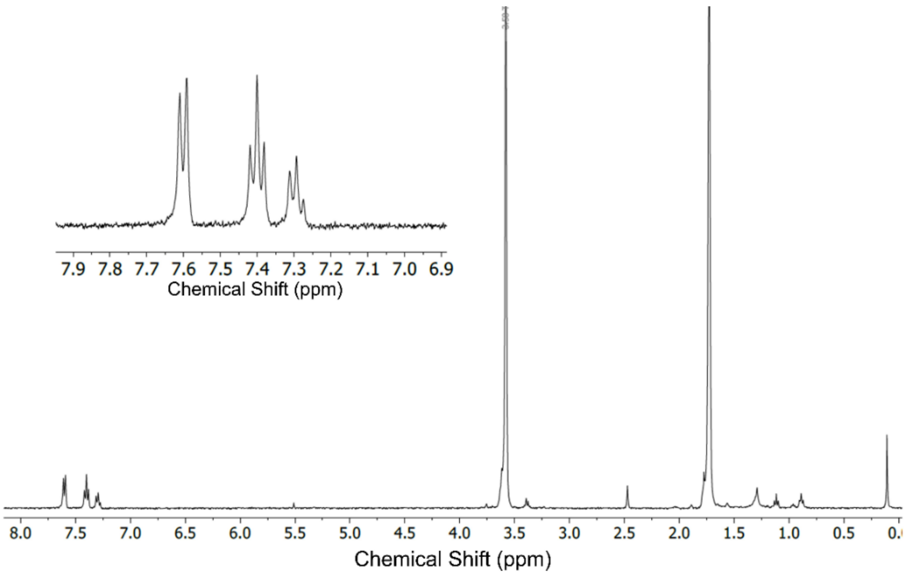

Figure S6. NMR analysis of the solution resulted after the reaction of Fe(BenzP*)Ph_2 (Complexes **3** and **4**) with 2 equiv of tert-butyl 2-bromopropionate. Reaction was performed under catalytically relevant conditions at 0 °C in THF. After the addition of electrophile, the solution immediately went from dark orange to colorless, indicating the formation of Fe(BenzP*)Br_2. Next, THF was evacuated under vacuum and 1 mL of hexane added to extract the organic products and remove Fe(BenzP*)Br_2 (since it is not soluble in hexane). Hexane solution was dried out under vacuum at RT, organic solids finally dissolved in d8-THF for NMR analysis. No quenching agent was added.

2.3. X-Ray Crystallography

Fe(BenzP*)Br_2 (1)

Data collection

A crystal (0.311 x 0.118 x 0.109 mm^3) was placed onto a thin glass optical fiber or a nylon loop and mounted on a Rigaku XtaLab Synergy-S Dualflex diffractometer equipped with a HyPix-6000HE HPC area detector for data collection at 99.9(3) K. A preliminary set of cell constants and an orientation matrix were calculated from a small sampling of reflections.[1] A short pre-experiment was run, from which an optimal data collection strategy was determined. The full data collection was carried out using a PhotonJet (Mo) X-ray Source with a frame time of 8.52 seconds and a detector distance of 34.0 mm. Series of frames were collected in 0.50° steps in ω at different 2θ, κ, and ϕ settings. After the intensity data were corrected for absorption, the final cell constants were calculated from the xyz centroids of 14872 strong reflections from the actual data collection after integration.[1] See Table S2 for additional crystal and refinement information.

Structure solution and refinement

The structure was solved using ShelXT[2] and refined using ShelXL.[3] The space group $P2_12_12_1$ was determined based on systematic absences. Most or all non-hydrogen atoms were assigned from the solution. Full-matrix least squares / difference Fourier cycles were performed which located any remaining non-hydrogen atoms. All non-hydrogen atoms were refined with anisotropic displacement parameters. All hydrogen atoms were placed in ideal positions and refined as riding atoms with relative isotropic displacement parameters. The

final full matrix least squares refinement converged to $R1 = 0.0499$ (F^2, $I > 2\sigma(I)$) and $wR2 = 0.1039$ (F^2, all data).

Structure description

The structure is the one suggested. The asymmetric unit contains one Fe complex in a general position and one-half of a cocrystallized diethyl ether molecule located along a crystallographic two-fold screw axis. The solvent is modeled as disordered over the axis (0.50:0.50).

Structure manipulation and figure generation were performed using Olex2.[4] Unless noted otherwise all structural diagrams containing thermal displacement ellipsoids are drawn at the 50 % probability level.

Data collection, structure solution, and structure refinement were conducted at the X-ray Crystallographic Facility, B04 Hutchison Hall, Department of Chemistry, University of Rochester. The instrument was purchased with funding from NSF MRI program grant CHE-1725028. All publications arising from this report MUST either 1) include William W. Brennessel as a coauthor or 2) acknowledge William W. Brennessel and the X-ray Crystallographic Facility of the Department of Chemistry at the University of Rochester.

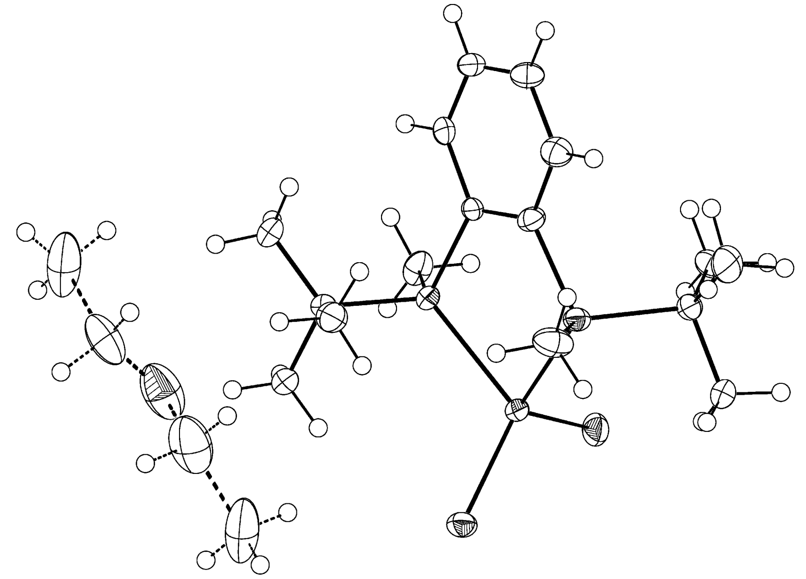

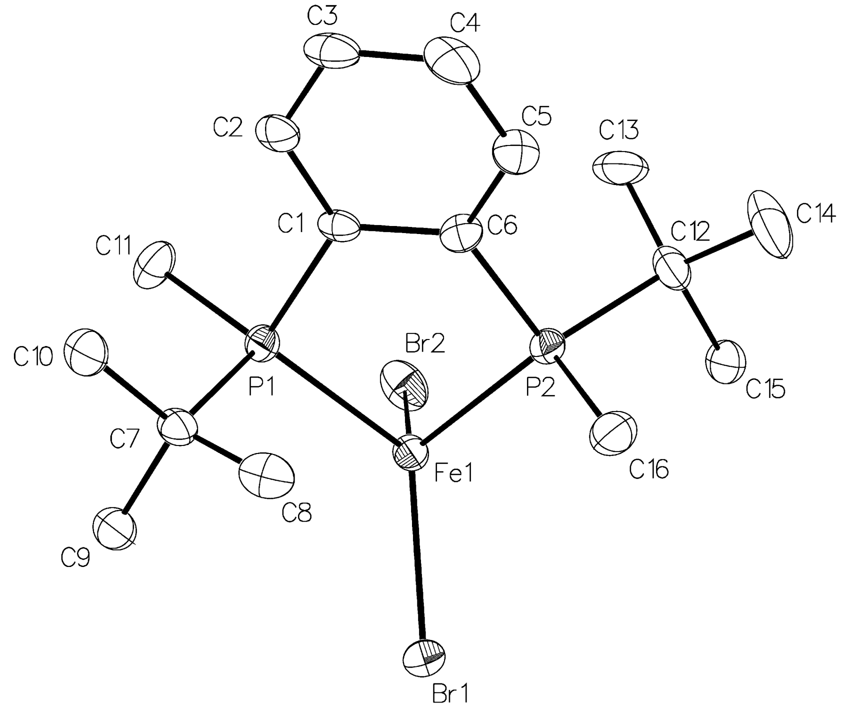
C3
C4
C2
C5
C13
C1
C6
C12
C14
C11
Br2
C10
P1
P2
C15
C7
Fe1
C16
C8
C9
Br1

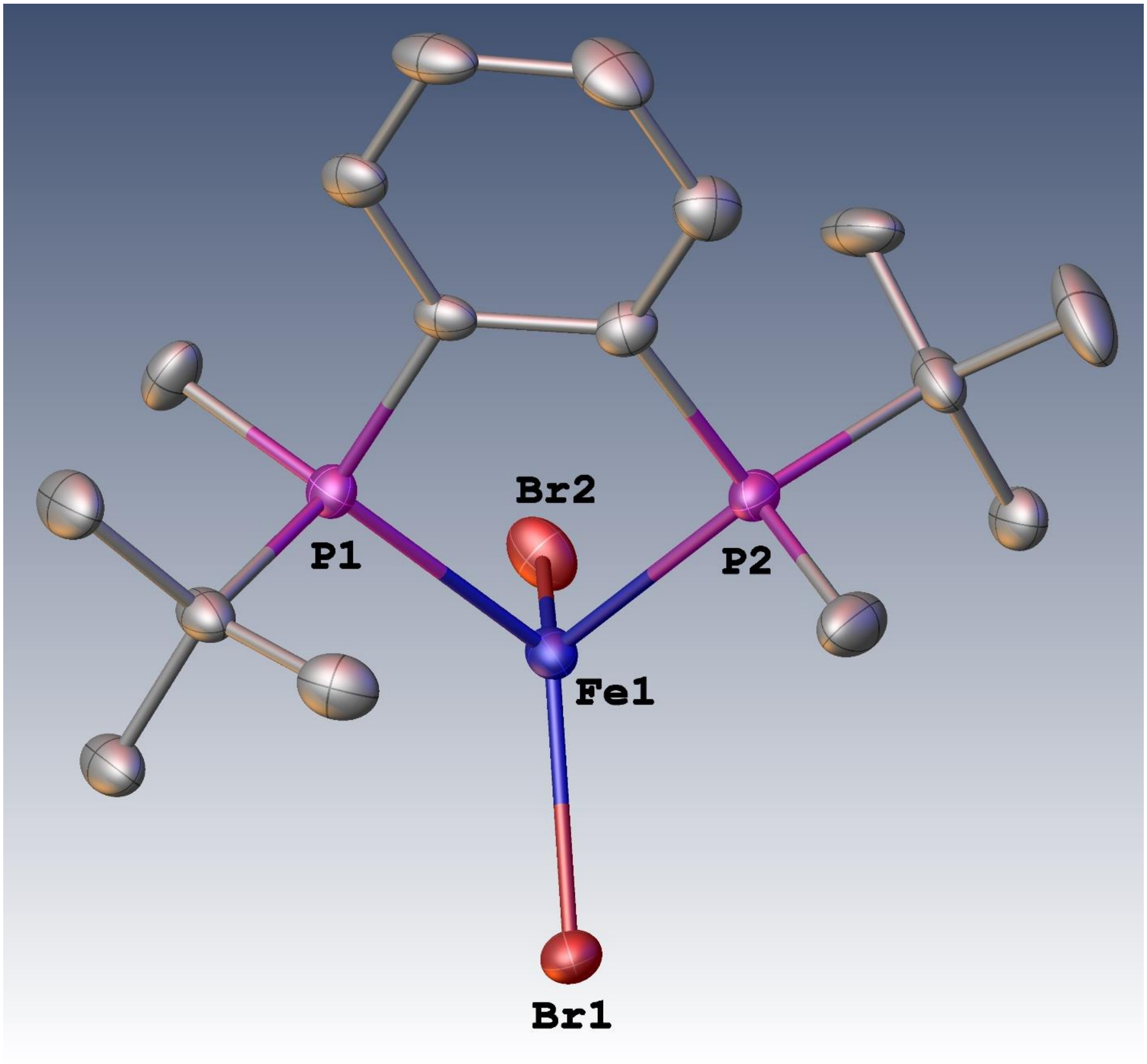
Br2
P1
P2
Fe1
Br1

Table S2. Crystal data and structure refinement for **1**

Identification code	Fe(BenzP*)Br_2	
Empirical formula	C18 H33 Br2 Fe O0.50 P2	
Formula weight	535.05	
Temperature	99.9(3) K	
Wavelength	0.71073 Å	
Crystal system	orthorhombic	
Space group	$P2_12_12_1$	
Unit cell dimensions	a = 9.6327(2) Å	α = 90°
	b = 11.1804(4) Å	β = 90°
	c = 22.2889(6) Å	γ = 90°
Volume	2400.46(12) Å^3	
Z	4	
Density (calculated)	1.481 Mg/m^3	
Absorption coefficient	4.092 mm^{-1}	
F(000)	1084	
Crystal color, morphology	colourless, needle	
Crystal size	0.311 x 0.118 x 0.109 mm^3	
Theta range for data collection	2.303 to 29.129°	
Index ranges	$-12 \leq h \leq 13$, $-15 \leq k \leq 14$, $-29 \leq l \leq 30$	
Reflections collected	28378	
Independent reflections	6459 [R(int) = 0.0474]	
Observed reflections	5859	
Completeness to theta = 29.129°	99.9%	
Absorption correction	Multi-scan	
Max. and min. transmission	1.00000 and 0.62821	
Refinement method	Full-matrix least-squares on F^2	
Data / restraints / parameters	6459 / 27 / 237	
Goodness-of-fit on F^2	1.232	
Final R indices [I>2sigma(I)]	$R1$ = 0.0499, $wR2$ = 0.1023	
R indices (all data)	$R1$ = 0.0571, $wR2$ = 0.1039	
Absolute structure parameter	0.033(6)	
Largest diff. peak and hole	0.654 and -0.776 e.Å^{-3}	

Fe(BenzP*)PhBr (2)

Data collection

A crystal (0.112 x 0.07 x 0.066 mm^3) was placed onto a thin glass optical fiber or a nylon loop and mounted on a Rigaku XtaLab Synergy-S Dualflex diffractometer equipped with a HyPix-6000HE HPC area detector for data collection at 99.99(10) K. A preliminary set of cell constants and an orientation matrix were calculated from a small sampling of reflections.[1] A short pre-experiment was run, from which an optimal data collection strategy was determined. The full data collection was carried out using a PhotonJet (Cu) X-ray Source with a frame time of 12.54 and 0.80 seconds and a detector distance of 31.2 mm. Series of frames were collected in 0.50° steps in ω at different 2θ, κ, and ϕ settings. After the intensity data were corrected for absorption, the final cell constants were calculated from the xyz centroids of 24680 strong reflections from the actual data collection after integration.[1] See Table S3 for additional crystal and refinement information.

Structure solution and refinement

The structure was solved using ShelXT[2] and refined using ShelXL.[3] The space group $P2_12_12_1$ was determined based on systematic absences. Most or all non-hydrogen atoms were assigned from the solution. Full-matrix least squares / difference Fourier cycles were performed which located any remaining non-hydrogen atoms. All non-hydrogen atoms were refined with anisotropic displacement parameters. All hydrogen atoms were placed in ideal positions and refined as riding atoms with relative isotropic displacement parameters. The final full matrix least squares refinement converged to $R1 = 0.0439$ (F^2, $I > 2\sigma(I)$) and $wR2 = 0.1143$ (F^2, all data).

Structure description

The structure is the one suggested. The asymmetric unit contains two independent molecules in general positions. Both molecules are modeled as disorders of two distinct species, ((R,R)-BenzP*)FeBr(Ph) and ((R,R)-BenzP*)$FeBr_2$, with site disorder ratios of 0.96:0.04 and 0.83:0.17, respectively, for molecules containing Fe1 and Fe2. The bromido ligand set of the minor component of disorder for molecule Fe2 is additionally modeled as disordered over two positions (0.90:0.10).

Structure manipulation and figure generation were performed using Olex2.[4] Unless noted otherwise all structural diagrams containing thermal displacement ellipsoids are drawn at the 50 % probability level.

Data collection, structure solution, and structure refinement were conducted at the X-ray Crystallographic Facility, B04 Hutchison Hall, Department of Chemistry, University of Rochester. The instrument was purchased with funding from NSF MRI program grant CHE-1725028. All publications arising from this report MUST either 1) include William W. Brennessel as a coauthor or 2) acknowledge William W. Brennessel and the X-ray Crystallographic Facility of the Department of Chemistry at the University of Rochester.

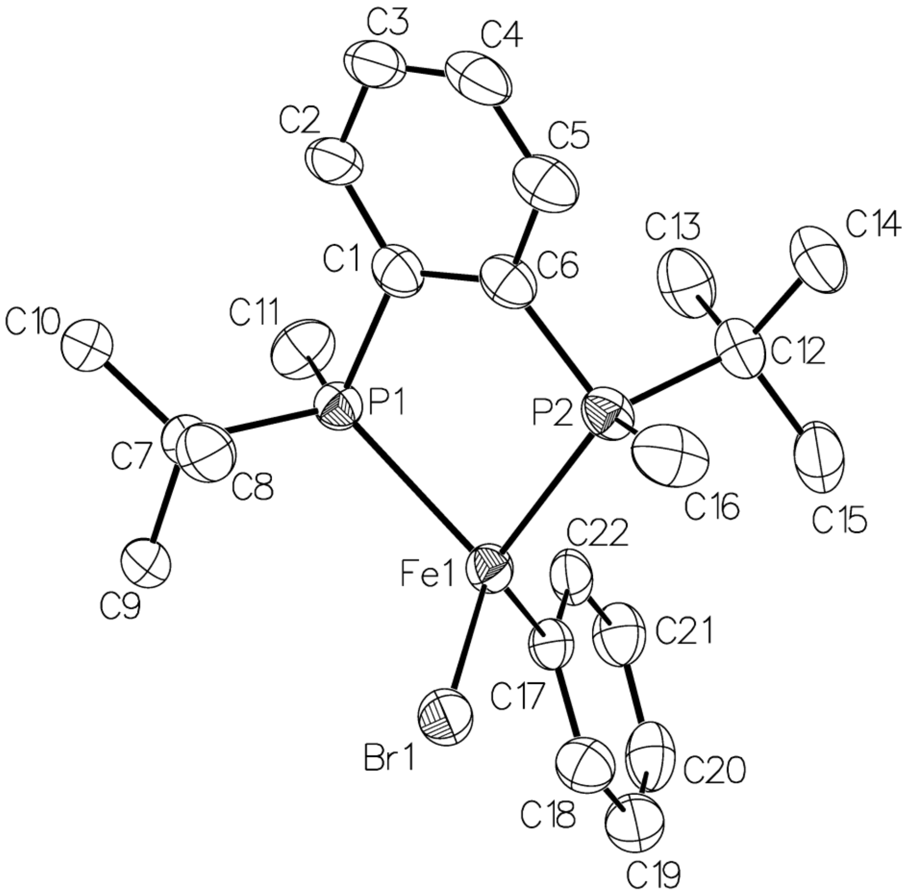
C3
C4
C2
C5
C13
C14
C1
C6
C10
C11
C12
P1
P2
C7
C8
C16
C15
C22
Fe1
C9
C21
C17
Br1
C20
C18
C19

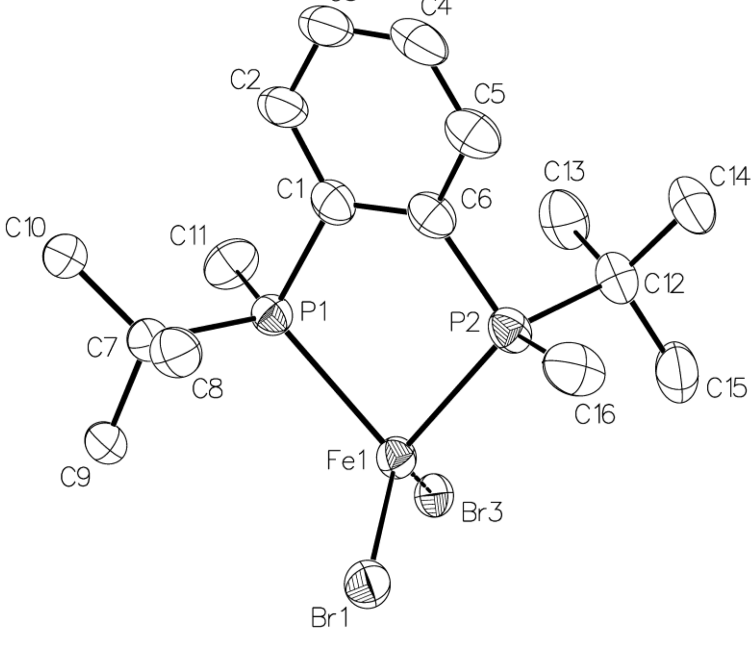
C3
C4
C2
C5
C13
C14
C1
C6
C10
C11
C12
P1
P2
C7
C8
C15
C16
Fe1
C9
Br3
Br1

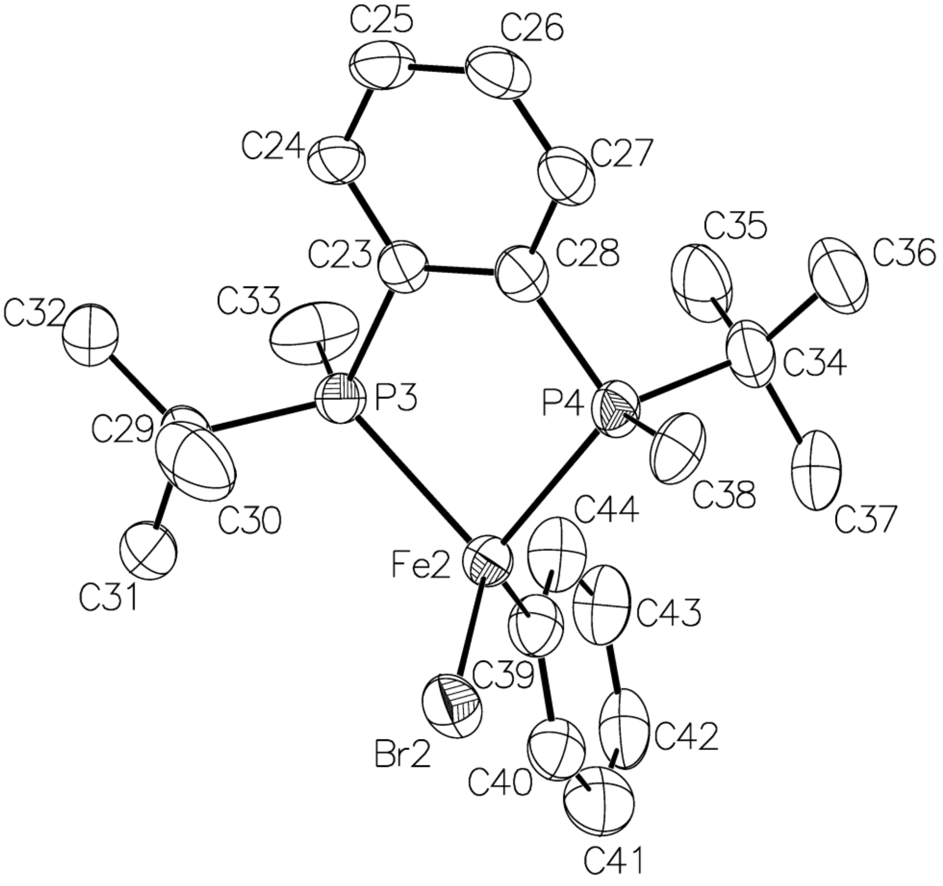
C25
C26
C24
C27
C23
C28
C35
C36
C32
C33
C34
P3
P4
C29
C30
C38
C37
C44
Fe2
C31
C43
C39
C42
Br2
C40
C41

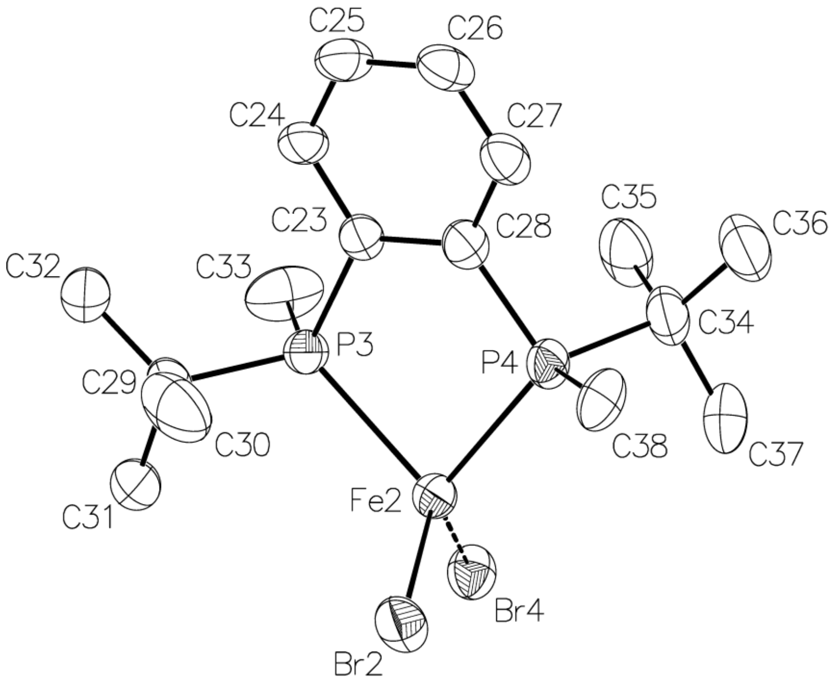
C25
C26
C24
C27
C23
C28
C35
C36
C32
C33
P3
C34
P4
C29
C30
C38
C37
Fe2
C31
Br4
Br2

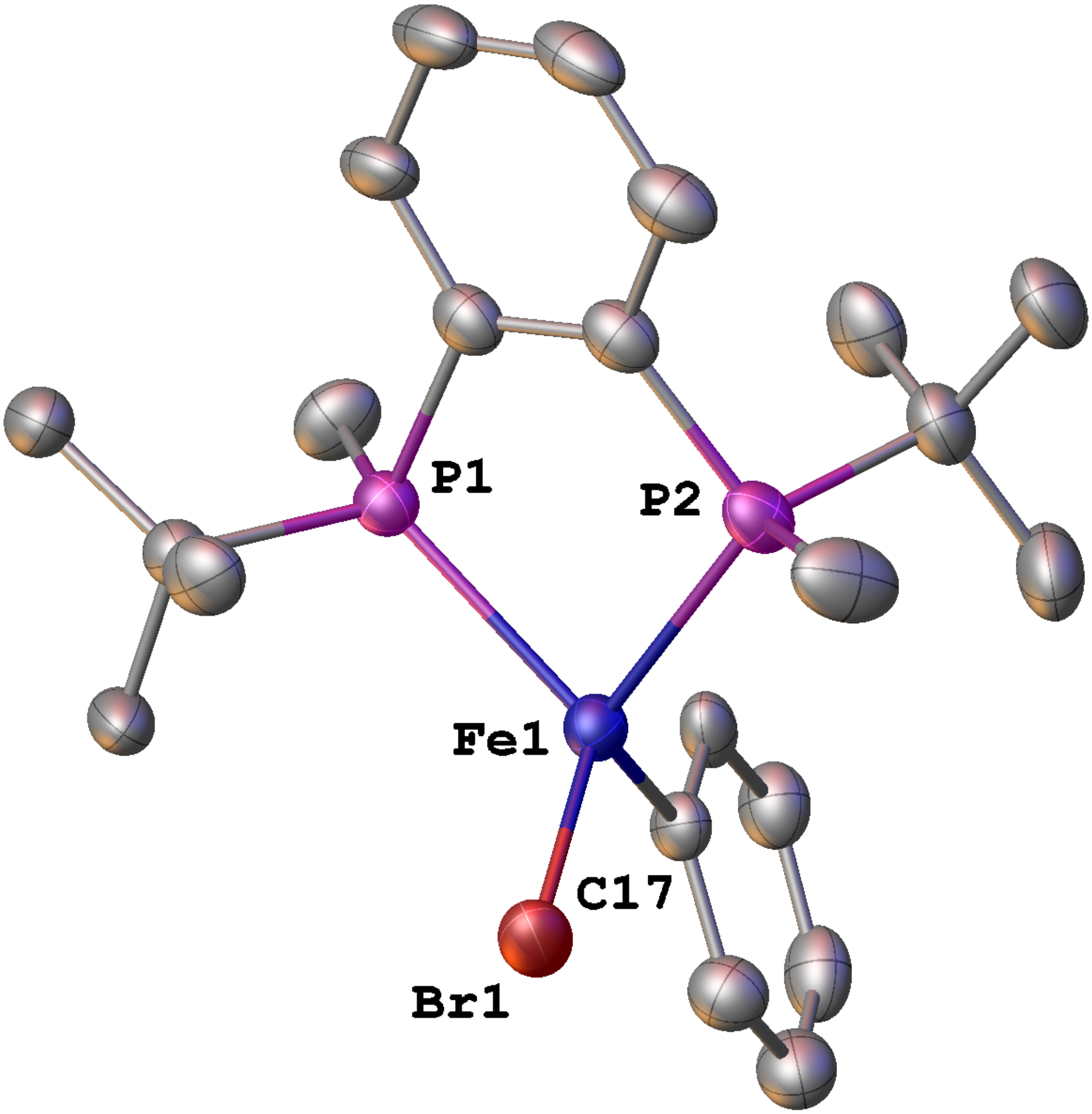
P1
P2
Fe1
C17
Br1

Table S3. Crystal data and structure refinement for **2**.

Identification code	Fe(BenzP*)PhBr	
Empirical formula	C21.34 H32.45 Br1.11 Fe P2	
Formula weight	495.49	
Temperature	99.99(10) K	
Wavelength	1.54184 Å	
Crystal system	orthorhombic	
Space group	$P2_12_12_1$	
Unit cell dimensions	*a* = 9.89150(12) Å	α = 90°
	b = 17.8509(2) Å	β = 90°
	c = 26.9080(4) Å	γ = 90°
Volume	4751.21(11) $Å^3$	
Z	8	
Density (calculated)	1.385 Mg/m^3	
Absorption coefficient	8.553 mm^{-1}	
F(000)	2043	
Crystal color, morphology	colourless, block	
Crystal size	0.112 x 0.07 x 0.066 mm^3	
Theta range for data collection	2.971 to 77.968°	
Index ranges	$-12 \le h \le 12$, $-22 \le k \le 22$, $-23 \le l \le 34$	
Reflections collected	44494	
Independent reflections	9952 [*R*(int) = 0.0562]	
Observed reflections	9205	
Completeness to theta = 74.504°	100.0%	
Absorption correction	Multi-scan	
Max. and min. transmission	1.00000 and 0.43152	
Refinement method	Full-matrix least-squares on F^2	
Data / restraints / parameters	9952 / 57 / 507	
Goodness-of-fit on F^2	1.051	
Final *R* indices [*I*>2sigma(*I*)]	*R*1 = 0.0439, *wR*2 = 0.1124	
R indices (all data)	*R*1 = 0.0475, *wR*2 = 0.1143	
Absolute structure parameter	0.004(3)	
Largest diff. peak and hole	1.015 and -0.472 e.$Å^{-3}$	

2.4. Computational Supporting Information

All geometry optimizations of intermediates and transition states were achieved using spin-unrestricted UB3LYP[5]-D3[6]/6-31G(d,p)[7]method, in THF solvent using the SMD solvent model[8] with "opt=noeigen" and "guess=mix" keywords as implemented in Gaussian16[9]. Frequency calculations were also done for all the stationary points, and transition states were characterized by the presence of one unique imaginary frequency, which suggested that they were first-order saddle points on the potential energy surface. Intrinsic Reaction Coordinate (IRC) calculations were done on the transition states to verify that they were the correct transition state associated with the reaction. The endpoint geometries obtained from the IRC calculations were further optimized to verify the authenticity of the transition state. The thermochemistry: enthalpy (ΔH) and free energy (ΔG) were obtained at the temperature of 298 K. All structural figures were generated with CYLview.[10] Distances in structural figures are shown in Å and energies are in kcal/mol.

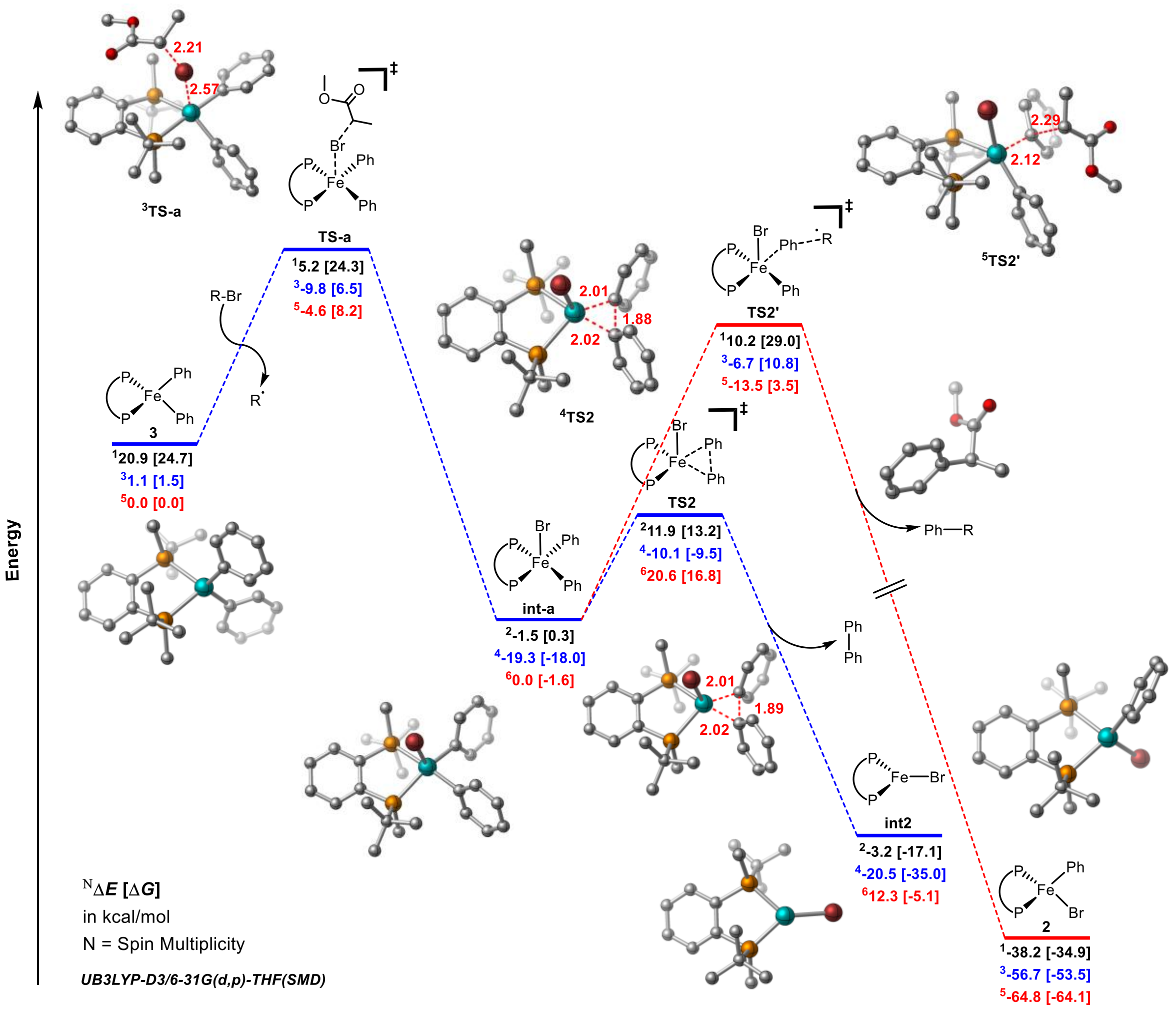

Figure S7. Gibbs free energy profile for halogen abstraction by bisphenylated Fe(II)-bisphosphine complexes and formation Fe(I)-Br complex along with biphenyl byproduct.

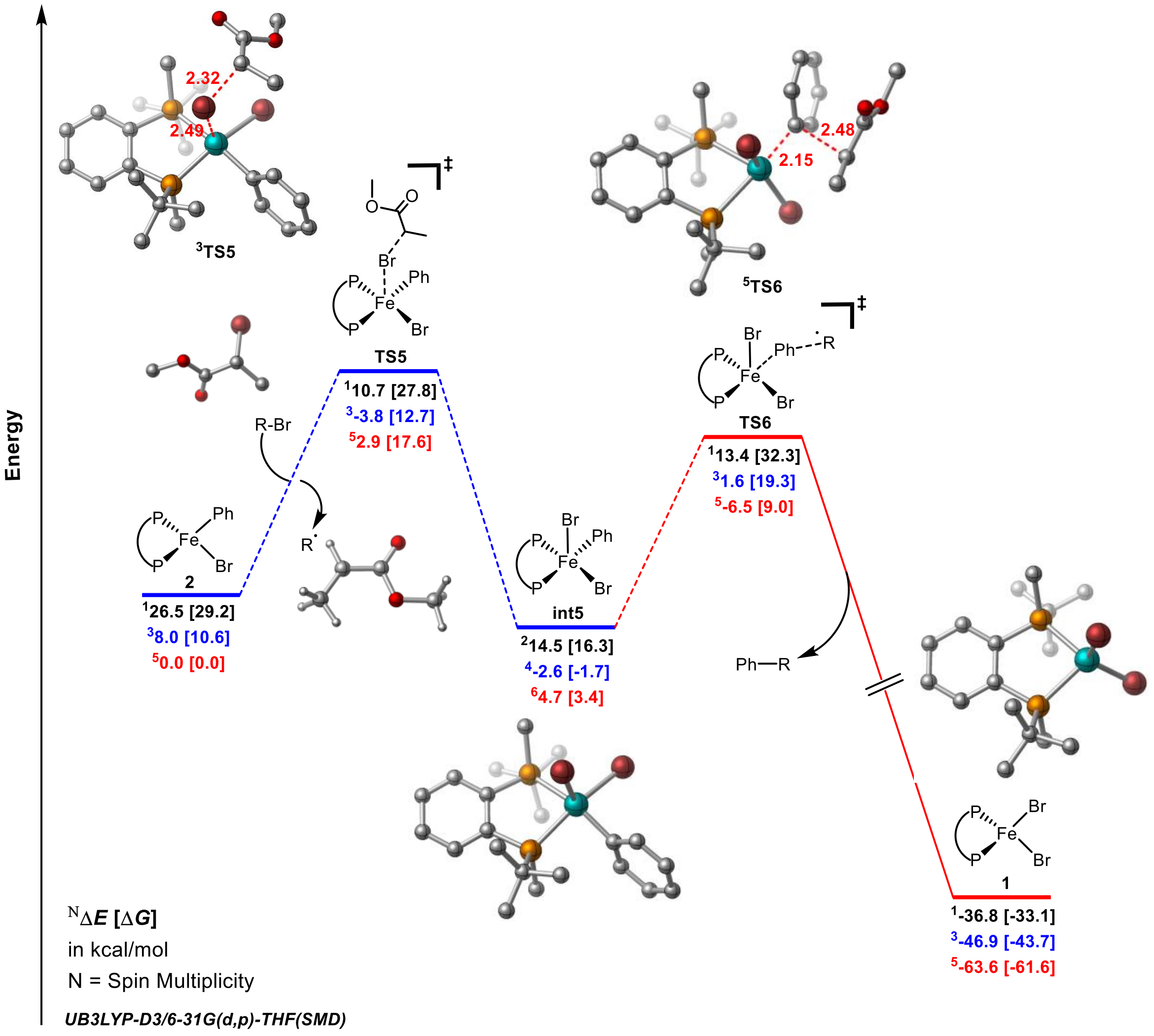

Figure S8. Gibbs free energy profile for 2-component coupling transformation involving only the monophenylated Fe(II) complex without formation of any Fe(I) complex.

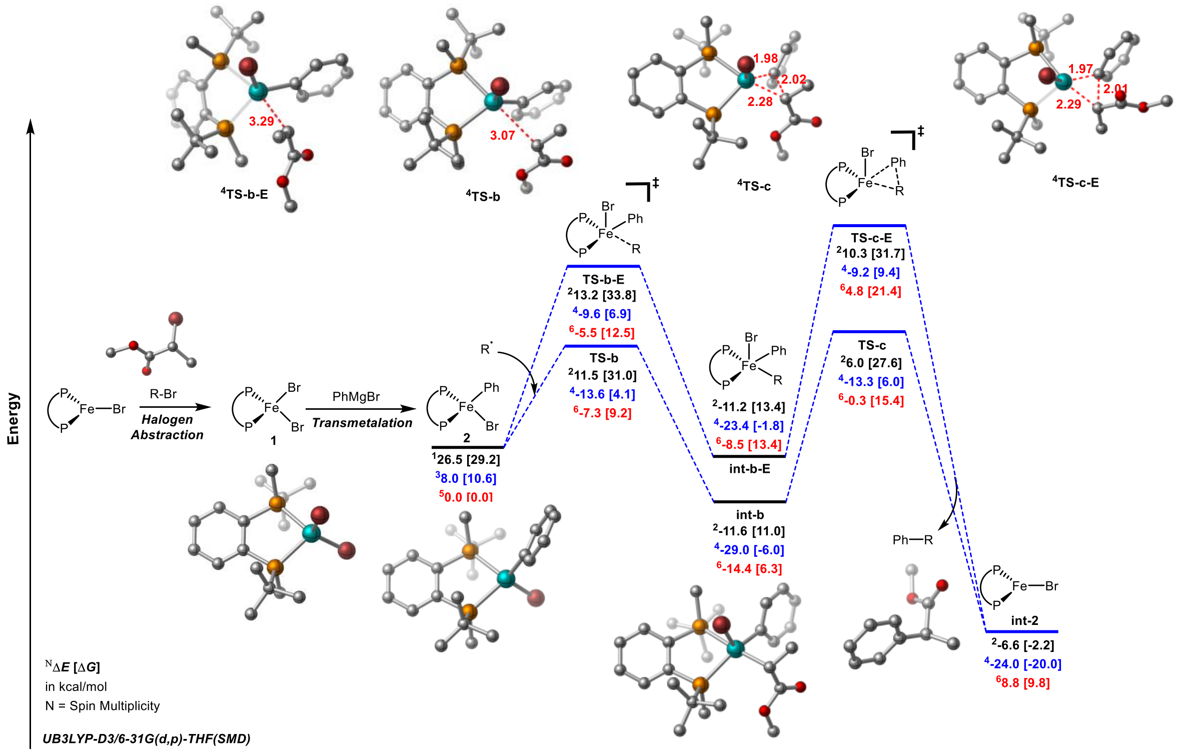

Figure S9. Gibbs free energy profile for radical recombination to monophenylated Fe(II)-bisphosphine complex and formation of 2 component coupling product along with regeneration of Fe(I)-Br complex.

Here in Figure **S9**, **TS-b** and **TS-b-E** corresponds to the two enantiomeric transition states for the radical recombination to iron step. Similarly, **TS-c** and **TS-c-E** correspond to the enantiomeric transition states for the reductive elimination step.

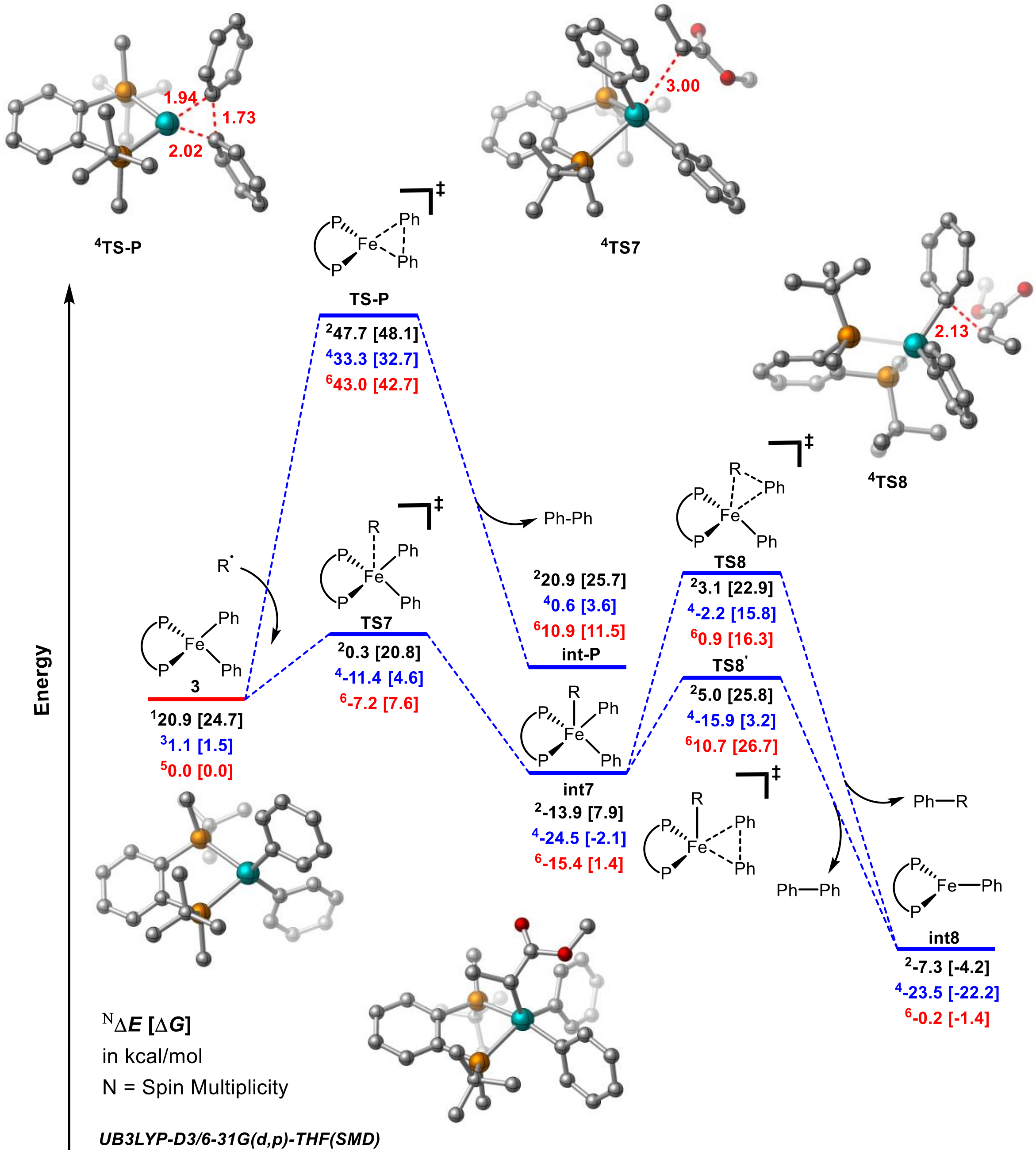

Figure S10. Gibbs free energy profile for radical recombination to bisphenylated Fe(II)-bisphosphine complex and formation of 2 component coupling product along with the competing pathway for the formation of biphenyl.

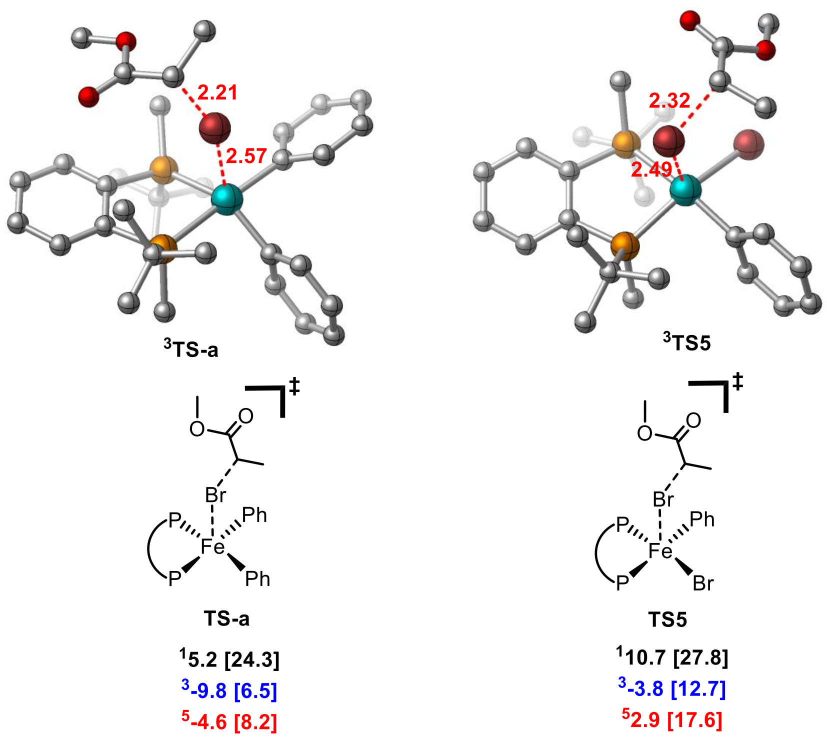

Figure S11. Relative energy barriers for the radical generation step involving bisphenylated iron as the active species (on left) and monophenylated iron as the active species (on the right).

The transition state for the radical generation step involving bisphenylated iron as the active species has lower barrier than that for the monophenylated iron as active species. This observation is consistent with our experimental results which show that the bisphenylated iron species is much more reactive than the monophenylated species.

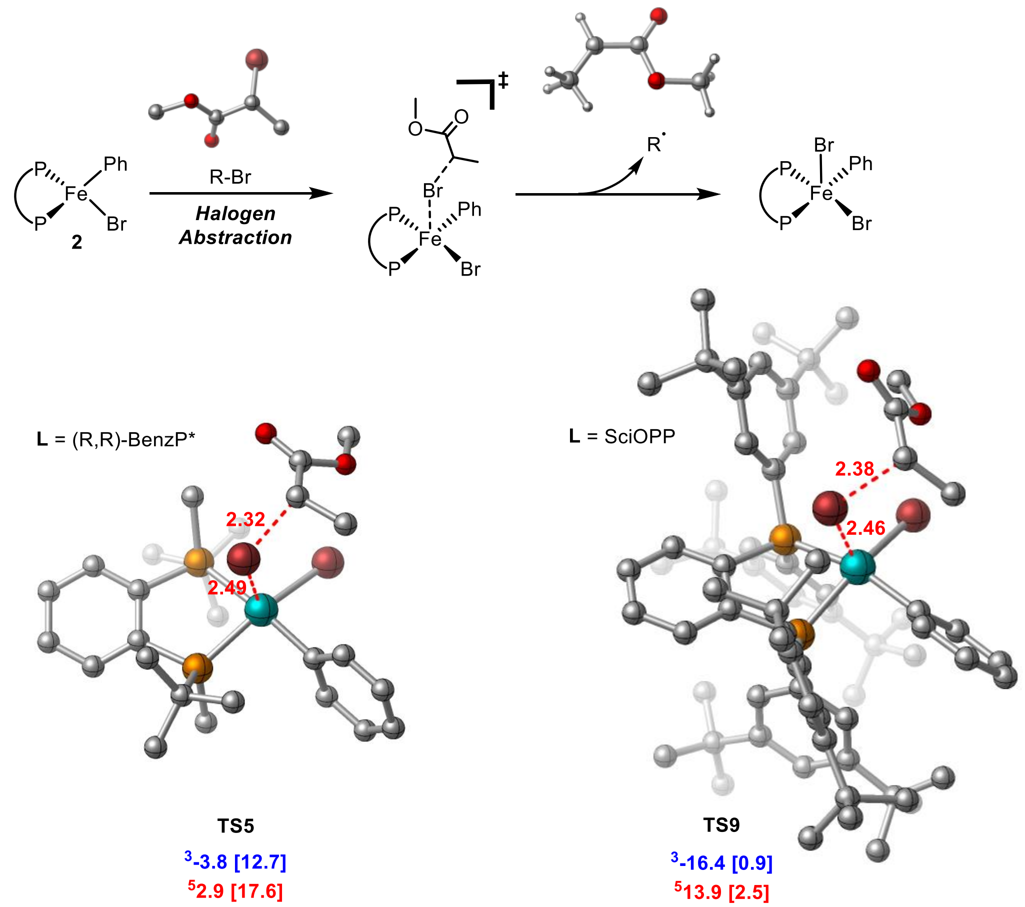

Figure S12. Comparison of energy barrier for the radical generation step involving BenzP* as the ligand (on left) vs SciOPP as ligand (on the right).

Table S4. Cartesian coordinates (xyz format) and energies of all the structures involved in each reaction mechanism calculated at the UB3LYP-D3/6-31G(d,p)-THF(SMD) level of theory.

^{1}1

E(scf) = -7716.60193814 a.u.

ν_{min} = 19.5 cm^{-1}

Br	9.460918	6.198996	14.155304	H	3.250192	6.917877	12.634534
Br	8.883892	10.054158	13.706880	H	3.859614	6.331031	11.085117
Fe	7.947846	8.001652	14.431085	H	3.813915	8.068140	11.402184
P	5.838978	8.285736	13.710873	C	5.370414	9.912797	13.016636
P	7.062434	7.436577	16.414965	H	5.563260	10.671567	13.778822
C	4.676679	8.014960	15.110735	H	4.317765	9.950704	12.726395
C	3.293462	8.228774	15.021397	H	6.000775	10.128239	12.152143
H	2.863190	8.654700	14.121148	C	7.582134	8.697820	17.719877
C	2.458687	7.892378	16.086785	C	7.046655	10.070431	17.269017
H	1.388663	8.060030	16.008049	H	7.423971	10.345755	16.278838
C	2.996342	7.327108	17.245820	H	7.378768	10.838067	17.977507
H	2.344474	7.042850	18.066499	H	5.952306	10.088974	17.240935
C	4.373837	7.140189	17.358125	C	7.065518	8.368036	19.127351
H	4.781221	6.710944	18.267347	H	5.974281	8.404744	19.181636
C	5.226687	7.508570	16.307457	H	7.453362	9.114288	19.831350
C	5.430505	6.976777	12.413709	H	7.401319	7.385443	19.472994
C	5.629747	5.599450	13.075247	C	9.124960	8.704150	17.712838
H	6.653814	5.470597	13.440571	H	9.538580	7.733748	18.006703
H	5.438283	4.812460	12.336697	H	9.487093	9.453228	18.426325
H	4.941257	5.449302	13.913269	H	9.524996	8.958239	16.725990
C	6.455389	7.170500	11.277624	C	7.422319	5.771072	17.083196
H	6.339381	8.141632	10.785587	H	7.118355	5.036018	16.333868
H	6.308737	6.391695	10.520448	H	6.886213	5.579544	18.016229
H	7.485013	7.094938	11.642688	H	8.497117	5.671272	17.244495
C	4.002983	7.092742	11.861110				

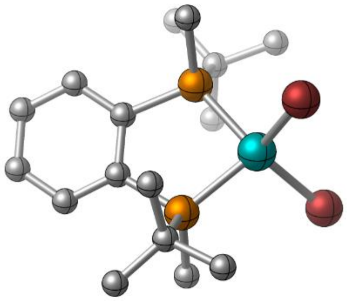

Zero-point correction= 0.409163 (Hartree/Particle)
Thermal correction to Energy= 0.436942
Thermal correction to Enthalpy= 0.437886
Thermal correction to Gibbs Free Energy= 0.350948
Sum of electronic and zero-point Energies= -7716.192775
Sum of electronic and thermal Energies= -7716.164996
Sum of electronic and thermal Enthalpies= -7716.164052
Sum of electronic and thermal Free Energies= -7716.250990

^{1}2

E(scf) = -5376.46585046 a.u.

ν_{min} = 17.8 cm^{-1}

Br	3.052809	6.142714	12.874912	C	5.753718	9.138859	16.809847
Fe	4.252877	7.981358	13.732831	H	6.190458	9.949514	16.224546
P	5.308512	7.749366	15.685722	H	6.479478	8.813087	17.559426
P	6.246670	7.879735	12.743042	H	4.862622	9.510297	17.320856
C	6.958464	7.014208	15.287871	C	7.231841	9.440529	12.316604
C	7.823272	6.468480	16.247531	C	7.249025	10.335837	13.566228
H	7.584182	6.540848	17.302389	H	6.238237	10.623976	13.863797
C	8.997018	5.822963	15.859446	H	7.816859	11.249542	13.353622
H	9.653635	5.397545	16.612762	H	7.731336	9.832019	14.410329
C	9.319602	5.713182	14.504761	C	8.677464	9.140904	11.885838
H	10.222220	5.192326	14.198917	H	9.264116	8.701108	12.696537
C	8.490389	6.289944	13.543567	H	9.162958	10.082056	11.599810
H	8.762691	6.224302	12.495396	H	8.728609	8.472878	11.020567
C	7.319605	6.961098	13.925094	C	6.491855	10.148810	11.167086
C	4.396189	6.524758	16.824314	H	6.487557	9.547281	10.252125
C	4.530625	5.106767	16.243755	H	7.002010	11.092247	10.938088
H	4.202212	5.069360	15.201609	H	5.459329	10.385735	11.432994
H	3.898426	4.419333	16.818806	C	6.281961	6.857079	11.214498
H	5.560430	4.741256	16.298551	H	5.964074	5.846274	11.478414
C	2.918515	6.969293	16.778197	H	7.266637	6.819426	10.741922
H	2.795360	8.006967	17.109193	H	5.558212	7.264445	10.504281
H	2.319615	6.332731	17.440347	C	3.723955	9.833041	13.682810
H	2.508874	6.880674	15.767189	C	3.129049	10.136881	12.431509
C	4.874140	6.537380	18.286034	H	3.208463	9.425832	11.607243
H	5.914647	6.221123	18.391991	C	2.446987	11.332191	12.192691
H	4.263122	5.830374	18.860754	H	2.012196	11.523366	11.214049
H	4.761072	7.519359	18.753711	C	2.322734	12.281737	13.211819

H	1.793951	13.214385	13.033828	C	3.572970	10.813735	14.683744
C	2.887022	12.013729	14.459965	H	4.000448	10.650132	15.666814
H	2.793790	12.740225	15.264624				

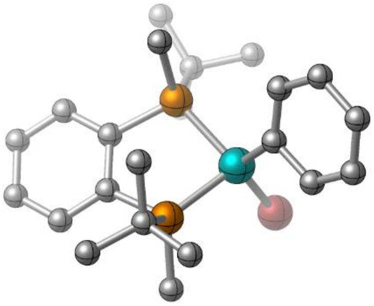

Zero-point correction= 0.497848 (Hartree/Particle)
Thermal correction to Energy= 0.529101
Thermal correction to Enthalpy= 0.530045
Thermal correction to Gibbs Free Energy= 0.436424
Sum of electronic and zero-point Energies= -5375.968003
Sum of electronic and thermal Energies= -5375.936750
Sum of electronic and thermal Enthalpies= -5375.935805
Sum of electronic and thermal Free Energies= -5376.029426

^{1}**3**

E(scf) = -3036.33656969 a.u.

ν_{min} = 35.2 cm^{-1}

C	-1.508613	-1.052690	0.162429	H	2.344977	0.509860	-3.509342
C	-1.886349	-2.090423	1.026265	C	2.555986	-2.859729	-3.832413
C	-2.331977	-0.731513	-0.940804	H	1.335408	-4.282095	-2.762786
C	-3.540853	-1.418194	-1.115763	H	3.592604	-1.198822	-4.737348
C	-3.078206	-2.787052	0.822079	H	3.106885	-3.617330	-4.383800
H	-3.355324	-3.594140	1.494109	C	0.698557	2.766301	-1.370484
C	-3.912627	-2.441142	-0.242083	C	-0.048397	3.964921	-1.400562
H	-4.847032	-2.972395	-0.398890	C	2.107372	2.941646	-1.364123
H	-1.248787	-2.363873	1.860427	C	0.549699	5.229587	-1.407617
H	-4.195427	-1.167111	-1.942889	H	-1.134071	3.918913	-1.405517
P	0.034048	-0.056467	0.378578	C	2.725798	4.196632	-1.370601
P	-1.702779	0.581026	-2.089083	H	2.761327	2.062516	-1.327745
Fe	0.438336	0.825597	-1.604464	C	1.942080	5.353243	-1.391332
C	-0.286657	1.039169	1.902503	H	-0.071077	6.123520	-1.425847
C	-2.131293	0.116110	-3.894291	H	3.811131	4.273717	-1.355439
C	1.092373	-0.867607	-2.379906	H	2.408663	6.335022	-1.393708
C	0.854885	-2.251451	-2.215185	C	1.275423	-1.264814	1.014881
C	2.107143	-0.539152	-3.311375	H	2.230537	-0.745755	1.134879
C	1.561744	-3.229074	-2.919020	H	0.996509	-1.711603	1.971559
H	0.070631	-2.577849	-1.534433	H	1.412781	-2.053638	0.275519
C	2.826230	-1.504528	-4.027912	C	-2.906037	1.944989	-1.762772

H	-2.765886	2.745325	-2.493056	H	0.079872	-0.473806	3.464582
H	-3.937036	1.586528	-1.819116	C	-1.873571	-1.374256	-4.164402
H	-2.732263	2.348828	-0.766796	H	-2.038610	-1.578240	-5.229778
C	1.015668	1.818017	2.169573	H	-0.850229	-1.661258	-3.923563
H	1.332124	2.384513	1.289026	H	-2.550462	-2.017837	-3.595119
H	0.851770	2.531898	2.986185	C	-1.162268	0.966452	-4.743509
H	1.835261	1.158109	2.472143	H	-1.369046	0.813003	-5.809822
C	-1.411139	2.026453	1.559583	H	-1.270071	2.036877	-4.532754
H	-2.345000	1.504656	1.326233	H	-0.121603	0.683914	-4.557740
H	-1.601148	2.680903	2.419115	C	-3.576489	0.457978	-4.304445
H	-1.135821	2.656541	0.711815	H	-3.727418	0.149671	-5.346414
C	-0.683693	0.247716	3.159813	H	-4.324241	-0.064251	-3.700878
H	-0.817772	0.948095	3.993842	H	-3.785118	1.529532	-4.251826
H	-1.629345	-0.284872	3.025348				

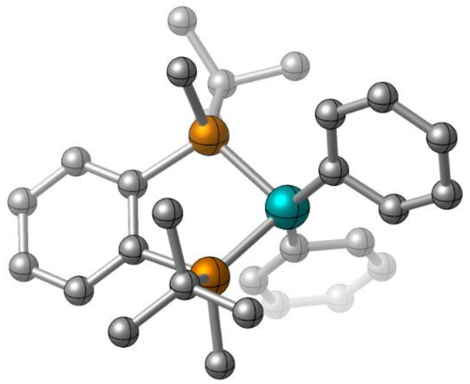

Zero-point correction= 0.586988 (Hartree/Particle)
Thermal correction to Energy= 0.621224
Thermal correction to Enthalpy= 0.622168
Thermal correction to Gibbs Free Energy= 0.524432
Sum of electronic and zero-point Energies= -3035.749582
Sum of electronic and thermal Energies= -3035.715346
Sum of electronic and thermal Enthalpies= -3035.714402
Sum of electronic and thermal Free Energies= -3035.812138

^{1}int-P
E(scf) = -3036.33649838 a.u.
ν_{min} = 24.7 cm^{-1}

C	-1.889344	-0.375902	-0.094822	H	-4.401583	-2.580947	-0.788646
C	-2.674198	-1.537029	-0.034629	C	-4.161060	-0.649376	-1.720412
C	-2.197535	0.617024	-1.047094	H	-5.055059	-0.739130	-2.331306
C	-3.358523	0.488066	-1.823914	H	-2.420068	-2.333413	0.656873
C	-3.799335	-1.678621	-0.848629	H	-3.638796	1.269138	-2.522752

P	-0.509369	0.029751	1.080031
P	-0.933335	1.960965	-1.244691
Fe	0.563139	1.855137	0.389401
C	0.333110	-1.601836	1.280307
C	-1.441357	0.258899	2.737680
C	-0.226163	1.609142	-2.988691
C	-1.978244	3.464041	-1.495505
C	2.483050	2.292980	1.126415
C	2.682542	1.379406	0.020900
C	2.746624	1.812294	2.442869
C	3.299319	0.120660	0.272650
H	2.736541	1.762130	-0.996180
C	3.299381	0.563305	2.653401
H	2.540366	2.466643	3.285535
C	3.587124	-0.290189	1.558086
H	3.533467	-0.524607	-0.570594
H	3.525450	0.239107	3.665539
H	4.034643	-1.264149	1.736043
C	1.786589	3.550265	0.859387
C	2.061565	4.356970	-0.281220
C	0.590173	3.844928	1.601345
C	1.281541	5.459723	-0.589087
H	2.925686	4.114639	-0.892930
C	-0.171870	4.994041	1.285079
H	0.410425	3.357911	2.555876
C	0.159013	5.789951	0.201105
H	1.542567	6.078103	-1.443610
H	-1.033463	5.239750	1.900455
H	-0.441959	6.660338	-0.046103
H	-2.497712	3.673189	-0.556285
H	-2.719044	3.360646	-2.293048
H	-1.331886	4.315812	-1.718138
H	0.815091	-1.848961	0.330584
H	1.114209	-1.509202	2.037337
H	-0.340591	-2.416776	1.558222
C	0.461746	0.233805	-2.926889
H	-0.260696	-0.567384	-2.738396
H	0.960032	0.020372	-3.880978
H	1.211886	0.199363	-2.131310
C	-1.259982	1.608507	-4.124671
H	-0.742962	1.463398	-5.082322
H	-1.983446	0.796315	-4.021600
H	-1.806929	2.554251	-4.192161
C	0.821648	2.701938	-3.267279
H	1.587351	2.730032	-2.489414
H	1.316672	2.506930	-4.227025
H	0.368485	3.697197	-3.323662
C	-2.347733	1.492233	2.576060
H	-1.772614	2.371692	2.271585
H	-3.122236	1.323920	1.820241
H	-2.847629	1.719064	3.526242
C	-0.372988	0.535899	3.811154
H	0.255910	1.387976	3.544430
H	-0.858519	0.757077	4.770118
H	0.284928	-0.326050	3.963769
C	-2.293359	-0.939754	3.181290
H	-2.724370	-0.729827	4.169059
H	-3.125319	-1.127423	2.498405
H	-1.706063	-1.858906	3.273013

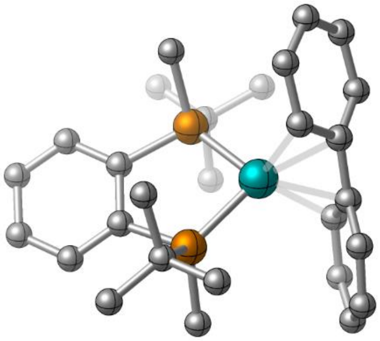

Zero-point correction= 0.587789 (Hartree/Particle)
Thermal correction to Energy= 0.621660
Thermal correction to Enthalpy= 0.622604
Thermal correction to Gibbs Free Energy= 0.525923

Sum of electronic and zero-point Energies= -3035.748709
Sum of electronic and thermal Energies= -3035.714838
Sum of electronic and thermal Enthalpies= -3035.713894
Sum of electronic and thermal Free Energies= -3035.810576

[1]TS2'
E(scf) = -5915.18985347 a.u.
ν_{min} = -496.8 cm^{-1}

C	-1.855132	-0.798570	0.340639
C	-2.612278	-1.842993	0.897143
C	-2.496870	0.433611	0.080144
C	-3.793364	0.653999	0.568473
C	-3.920157	-1.629297	1.331191
H	-4.482273	-2.447892	1.770878
C	-4.491784	-0.360837	1.220213
H	-5.493525	-0.175112	1.596409
H	-2.183076	-2.830722	1.001019
H	-4.279715	1.609610	0.404311
P	-0.029235	-0.907530	0.036617
P	-1.731270	1.551246	-1.164946
Fe	0.684449	1.275492	-0.973028
C	0.756583	-1.502657	1.690303
C	-2.619811	0.995022	-2.762687
C	2.531052	0.638140	-0.729322
C	3.404473	1.064775	0.288796
C	3.027702	-0.334690	-1.618678
C	4.705672	0.568067	0.397719
H	3.064149	1.811031	0.998021
C	4.328183	-0.841049	-1.517665
H	2.388923	-0.724736	-2.409182
C	5.177893	-0.388411	-0.506230
H	5.354121	0.929281	1.193113
H	4.674090	-1.590897	-2.225738
H	6.189491	-0.776150	-0.421475
Br	0.463383	2.749899	0.968167
C	3.295557	4.249609	-0.880690
H	2.937745	5.231913	-1.199096
H	2.642400	3.876631	-0.090449
H	4.302196	4.379986	-0.457328
C	3.703318	3.877131	-3.340894
O	3.591993	5.052521	-3.669211
O	4.181135	2.926804	-4.195560
C	4.427081	3.376345	-5.532910
H	4.767649	2.499311	-6.086265
H	3.513591	3.773371	-5.985871
H	5.197381	4.153517	-5.554796
C	3.341064	3.302975	-2.042986
H	3.770768	2.328690	-1.862568
C	1.337124	2.599967	-2.361960
C	0.661672	3.865053	-2.415755
C	1.426776	1.898401	-3.606674
C	0.275567	4.441098	-3.614823
H	0.515977	4.408600	-1.488238
C	1.025355	2.472420	-4.811182
H	1.927093	0.935948	-3.632956
C	0.477801	3.759812	-4.828031
H	-0.184185	5.426297	-3.619524
H	1.156572	1.925510	-5.741570
H	0.186410	4.218920	-5.768232
C	0.169327	-2.379110	-1.056244
H	1.230443	-2.542584	-1.251782
H	-0.251419	-3.276256	-0.593409
H	-0.333196	-2.202314	-2.007786
C	2.166320	-2.053598	1.391579
H	2.127357	-2.991465	0.829510
H	2.792121	-1.347453	0.852385
H	2.657589	-2.268870	2.348240
C	0.853986	-0.298994	2.638408

H	1.525553	0.465832	2.251432	H	-4.564566	0.521258	-3.561327
H	-0.124226	0.162179	2.805136	H	-4.323167	-0.118837	-1.931399
H	1.238349	-0.639470	3.608138	H	-4.650106	1.605699	-2.171697
C	-0.029310	-2.620453	2.398313	C	-2.422621	2.106569	-3.803596
H	0.574738	-2.972566	3.243456	H	-1.370407	2.341032	-3.945529
H	-0.979577	-2.269690	2.804931	H	-2.823776	1.773629	-4.768814
H	-0.214280	-3.485183	1.753239	H	-2.947454	3.026372	-3.528644
C	-2.458176	3.203847	-0.810986	C	-1.944242	-0.296239	-3.247604
H	-3.518892	3.253286	-1.068544	H	-2.083208	-1.108059	-2.528433
H	-2.323495	3.422743	0.248335	H	-2.39303	-0.614904	-4.196115
H	-1.912776	3.952497	-1.387649	H	-0.871307	-0.156063	-3.41829
C	-4.123918	0.737278	-2.580035				

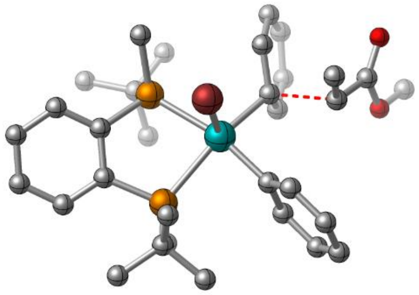

Zero-point correction= 0.696361 (Hartree/Particle)
Thermal correction to Energy= 0.740198
Thermal correction to Enthalpy= 0.741143
Thermal correction to Gibbs Free Energy= 0.622835
Sum of electronic and zero-point Energies= -5914.493493
Sum of electronic and thermal Energies= -5914.449655
Sum of electronic and thermal Enthalpies= -5914.448711
Sum of electronic and thermal Free Energies= -5914.567019

^{1}TS-a

E(scf) = -5915.19780852 a.u.

ν_{min} = -1022.1 cm^{-1}

Fe	-0.064279	-0.406835	0.082227	C	-4.410973	1.046385	-0.748806
C	3.856186	-0.741467	-0.022012	C	-5.076679	2.216318	-0.382711
C	4.267809	-2.067668	0.546393	C	-4.367080	3.272502	0.189897
P	-0.485558	1.776573	0.181346	C	-2.989648	3.162091	0.378504
C	0.301676	3.006752	-1.036934	P	-2.057857	-0.605357	-0.966340
C	-0.339216	2.823252	-2.419690	C	-3.150798	-2.158313	-0.694611
C	-2.308799	1.994948	0.003720	C	-3.899857	-2.106585	0.647007
C	-3.030418	0.914066	-0.547544	C	-0.061160	2.534178	1.810395

C	-2.025097	-0.508653	-2.807983
C	-2.177503	-3.354360	-0.688088
C	-4.167579	-2.384946	-1.833591
C	0.115836	4.473083	-0.605249
C	1.805357	2.682274	-1.091372
C	-0.974753	-0.810623	1.833817
C	-0.576955	-2.081002	2.318476
C	-1.119054	-2.659478	3.468173
C	-2.078225	-1.969106	4.216199
C	-2.479179	-0.703663	3.785652
C	-1.939876	-0.148618	2.619274
C	4.700017	0.449536	0.171649
O	5.442649	0.378852	1.303985
O	4.721963	1.416212	-0.578056
H	-2.453282	3.988626	0.830913
H	-4.883034	4.179801	0.490010
H	-6.148664	2.297377	-0.537089
H	-4.980264	0.233175	-1.181766
H	1.005289	2.767686	1.818647
H	-0.623624	3.450638	2.000402
H	-0.259676	1.815534	2.604258
H	-1.603255	-1.429230	-3.215683
H	-3.026399	-0.346891	-3.213244
H	-1.386252	0.317737	-3.111832
H	0.598274	5.118505	-1.349810
H	-0.937603	4.762273	-0.561643
H	0.579412	4.690963	0.360214
H	2.293271	3.346261	-1.815169
H	2.296447	2.833842	-0.126645
H	1.998453	1.654148	-1.395721
H	-0.231593	1.802321	-2.782714
H	-1.405174	3.071237	-2.411211
H	0.154305	3.483748	-3.142394
H	-2.751988	-4.286692	-0.626675
H	-1.570489	-3.393980	-1.597613
H	-1.507090	-3.320945	0.173193
H	-4.759434	-3.276224	-1.592792
H	-4.868992	-1.556758	-1.961017
H	-3.682400	-2.570538	-2.794752
H	-3.217382	-2.074477	1.493601
H	-4.570922	-1.246123	0.717391
H	-4.510612	-3.012897	0.742873
H	0.184008	-2.649825	1.783790
H	-2.319588	0.820907	2.310885
H	-0.785254	-3.644718	3.787451
H	-3.225951	-0.148841	4.350299
H	-2.499826	-2.408905	5.116211
H	4.534764	-1.993346	1.602694
H	3.460553	-2.794453	0.432748
H	5.144564	-2.450267	0.004407
H	3.385683	-0.754576	-0.999184
Br	1.971492	-0.182512	1.227860
C	6.237762	1.540737	1.590487
H	6.757321	1.322872	2.524437
H	6.961044	1.727959	0.791515
H	5.603604	2.424437	1.708842
C	0.826522	-1.110802	-1.571112
C	1.127213	-2.482797	-1.380648
C	1.350253	-0.545000	-2.747888
C	1.898877	-3.225139	-2.278234
H	0.762100	-3.001890	-0.494210
C	2.132590	-1.270113	-3.656232
H	1.1656	0.498992	-2.974336
C	2.415442	-2.617082	-3.426354
H	2.104691	-4.274534	-2.077669
H	2.523329	-0.776569	-4.54382
H	3.023259	-3.18369	-4.126827

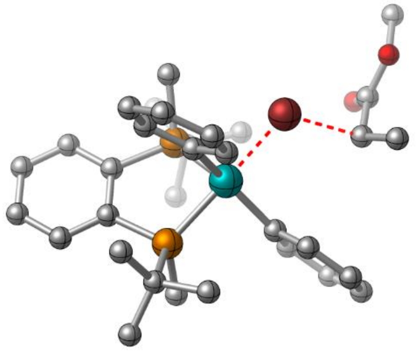

Zero-point correction= 0.697311 (Hartree/Particle)
Thermal correction to Energy= 0.740930
Thermal correction to Enthalpy= 0.741874
Thermal correction to Gibbs Free Energy= 0.623154
Sum of electronic and zero-point Energies= -5914.500498
Sum of electronic and thermal Energies= -5914.456879
Sum of electronic and thermal Enthalpies= -5914.455935
Sum of electronic and thermal Free Energies= -5914.574654

^{1}TSP

E(scf) = -3036.29386497 a.u.

ν_{min} = -369.3 cm^{-1}

C	-2.379803	-0.726939	-0.265175	C	2.669745	1.124498	0.133558
C	-3.170532	-1.884631	-0.211935	C	2.281749	1.832204	2.422245
C	-2.691087	0.280022	-1.206150	C	3.774281	0.381819	0.555495
C	-3.853410	0.158265	-1.982235	H	2.480934	1.209054	-0.936339
C	-4.295282	-2.017531	-1.027578	C	3.394112	1.113121	2.824770
H	-4.897686	-2.920228	-0.976896	H	1.733133	2.422619	3.151438
C	-4.654950	-0.980784	-1.891393	C	4.141939	0.353984	1.902515
H	-5.547215	-1.065759	-2.505329	H	4.371129	-0.152869	-0.180806
H	-2.916334	-2.688086	0.471676	H	3.693760	1.134648	3.870616
H	-4.134011	0.947771	-2.671768	H	5.016717	-0.201613	2.227107
P	-0.982642	-0.359486	0.905473	C	0.995680	3.236564	0.492781
P	-1.431684	1.631398	-1.407962	C	1.718445	4.091673	-0.384719
Fe	0.147832	1.445921	0.167052	C	0.166757	3.891220	1.454752
C	-0.099085	-1.972910	1.043857	C	1.550759	5.471386	-0.371741
C	-1.868813	-0.138334	2.577091	H	2.419577	3.648262	-1.087380
C	-0.724240	1.322755	-3.149838	C	-0.010935	5.270187	1.456506
C	-2.453405	3.157665	-1.576406	H	-0.347812	3.293312	2.205027
C	1.817943	1.800912	1.071090	C	0.672608	6.078328	0.538267

H	2.109673	6.086146	-1.074353	C	0.367677	2.388139	-3.364874
H	-0.678402	5.723658	2.186233	H	1.139079	2.325917	-2.592284
H	0.547265	7.157054	0.550548	H	0.848103	2.237809	-4.339715
H	-2.922538	3.354295	-0.607619	H	-0.039387	3.404323	-3.346046
H	-3.233950	3.095768	-2.339972	C	-2.810782	1.069466	2.426452
H	-1.790687	3.997201	-1.803684	H	-2.268425	1.962622	2.096963
H	0.368435	-2.184659	0.077457	H	-3.607481	0.873552	1.701576
H	0.695462	-1.873259	1.788393	H	-3.281213	1.298758	3.390441
H	-0.742728	-2.814392	1.314113	C	-0.769331	0.192299	3.602699
C	-0.075881	-0.073191	-3.129789	H	-0.199240	1.075460	3.304431
H	-0.817940	-0.862834	-2.974389	H	-1.222074	0.391318	4.582081
H	0.428332	-0.265830	-4.084692	H	-0.060987	-0.633908	3.725305
H	0.672044	-0.154153	-2.331910	C	-2.668224	-1.357843	3.057186
C	-1.748385	1.401096	-4.290258	H	-3.084460	-1.149658	4.051378
H	-1.232891	1.271008	-5.250622	H	-3.507696	-1.584734	2.395294
H	-2.503003	0.613963	-4.218048	H	-2.044633	-2.253128	3.146972
H	-2.258051	2.369361	-4.322818				

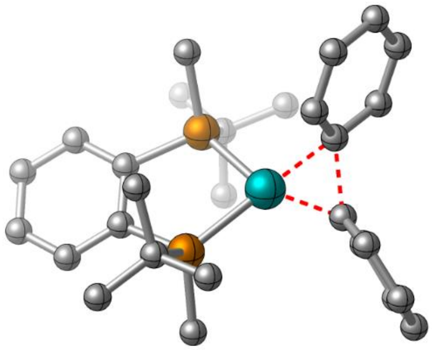

Zero-point correction= 0.583434 (Hartree/Particle)
Thermal correction to Energy= 0.617847
Thermal correction to Enthalpy= 0.618792
Thermal correction to Gibbs Free Energy= 0.518973
Sum of electronic and zero-point Energies= -3035.710431
Sum of electronic and thermal Energies= -3035.676018
Sum of electronic and thermal Enthalpies= -3035.675073
Sum of electronic and thermal Free Energies= -3035.774892

^{2}int2
E(scf) = -5144.78939961 a.u.
ν_{min} =22.0 cm^{-1}

Br	2.222953	8.658571	13.327053	C	7.343985	10.208657	13.418421
Fe	4.459620	8.347489	13.861081	H	7.834497	11.133831	13.092875
P	5.390913	7.809254	15.778618	H	6.336897	10.469183	13.768686
P	6.343421	7.691166	12.911689	H	7.903643	9.809397	14.270711
C	7.099986	7.143215	15.517142	C	8.708155	8.905385	11.754073
C	7.986165	6.776562	16.540227	H	9.149255	9.825235	11.349232
H	7.726624	6.955075	17.578445	H	9.360276	8.554531	12.558317
C	9.208034	6.174171	16.236117	H	8.716405	8.159655	10.952599
H	9.884900	5.889903	17.036840	C	6.439185	9.778835	11.108029
C	9.552755	5.925685	14.905296	H	6.398462	9.101222	10.248968
H	10.493180	5.436396	14.667930	H	5.411502	9.975986	11.433918
C	8.697242	6.325315	13.877439	H	6.872984	10.725886	10.764528
H	8.986935	6.151029	12.846167	C	3.030665	6.938057	16.901709
C	7.482179	6.959678	14.172474	H	2.589272	7.313836	15.973323
C	7.288776	9.199955	12.255555	H	2.395265	6.130804	17.285988
C	4.456649	6.408622	16.657211	H	3.018993	7.749294	17.637901
C	5.648173	9.105390	17.063126	C	5.073639	5.946976	17.984101
H	4.678822	9.501058	17.378637	H	4.415958	5.199966	18.446711
H	6.190712	8.747831	17.942682	H	6.049444	5.476113	17.841168
H	6.218066	9.920304	16.606573	H	5.184873	6.769516	18.698033
C	6.376906	6.457840	11.543686	C	4.406163	5.232550	15.664969
H	7.376968	6.275313	11.139730	H	3.822398	4.408091	16.091859
H	5.975424	5.518845	11.936327	H	3.933283	5.530213	14.722081
H	5.722381	6.799502	10.737624	H	5.407965	4.854890	15.434378

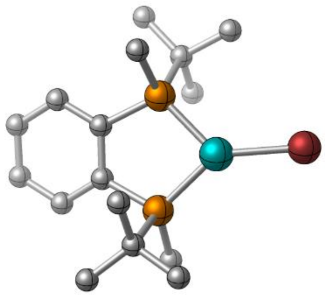

Zero-point correction= 0.406455 (Hartree/Particle)
Thermal correction to Energy= 0.432423
Thermal correction to Enthalpy= 0.433367
Thermal correction to Gibbs Free Energy= 0.350438
Sum of electronic and zero-point Energies= -5144.382944
Sum of electronic and thermal Energies= -5144.356977
Sum of electronic and thermal Enthalpies= -5144.356033
Sum of electronic and thermal Free Energies= -5144.438961

^{2}int7
E(scf) = -3343.46406037 a.u.
ν_{min} = 21.5 cm^{-1}

C	-1.772136	-0.932242	0.626613
C	-2.474963	-1.787279	1.490638
C	-2.431018	0.211825	0.113652
C	-3.730395	0.507072	0.553019
C	-3.774547	-1.492461	1.899830
H	-4.291522	-2.163529	2.579417
C	-4.395372	-0.327502	1.449374
H	-5.398007	-0.075203	1.781990
H	-2.005008	-2.686412	1.867317
H	-4.238735	1.391431	0.185827
P	0.026708	-1.160179	0.228850
P	-1.578647	1.223443	-1.182063
Fe	0.797826	0.866766	-0.989709
C	0.917304	-1.834420	1.803612
C	-2.435667	0.632846	-2.797405
C	2.673715	0.268826	-1.128343
C	3.833860	0.870165	-0.607803
C	2.876328	-0.911901	-1.876624
C	5.107597	0.321674	-0.799321
H	3.746733	1.793331	-0.046149
C	4.141845	-1.468576	-2.082460
H	2.025506	-1.426377	-2.319613
C	5.271018	-0.855088	-1.533307
H	5.977391	0.819397	-0.374651
H	4.244899	-2.383392	-2.662698
H	6.259060	-1.283965	-1.679487
C	1.017692	1.976635	-2.636909
C	0.745363	3.351780	-2.772279
C	1.409289	1.309012	-3.817584
C	0.854320	4.019730	-3.996836
H	0.419140	3.923787	-1.912466
C	1.500421	1.958495	-5.052746
H	1.649915	0.250821	-3.786495
C	1.226501	3.324618	-5.149283
H	0.633530	5.083840	-4.050525
H	1.794049	1.397533	-5.937458
H	1.303709	3.837581	-6.104399
C	0.967557	2.107577	0.702442
C	-0.150260	2.082142	1.749313
H	-0.977679	2.736153	1.478448
H	-0.560423	1.088038	1.904619
H	0.222134	2.434599	2.722572
C	1.262838	3.512956	0.338843
O	0.454211	4.436298	0.331268
O	2.563042	3.721574	0.000348
C	2.899616	5.045185	-0.438433
H	3.975163	5.029770	-0.621205
H	2.371560	5.296670	-1.362163
H	2.657699	5.787785	0.327319
H	1.874121	1.658333	1.118382
C	0.075771	-2.653235	-0.862198
H	-0.198551	-2.384569	-1.880817
H	1.093878	-3.046323	-0.875723
H	-0.606564	-3.426885	-0.502309
C	0.682109	-3.343206	2.030233
H	1.238555	-3.643737	2.926072
H	-0.364129	-3.602594	2.203539
H	1.052049	-3.953700	1.203746
C	0.504546	-1.065326	3.069561
H	0.815228	-0.020655	3.027810
H	-0.571979	-1.100465	3.256510
H	1.005882	-1.518825	3.932930
C	2.426326	-1.623481	1.573317
H	2.974337	-2.038373	2.428131
H	2.785893	-2.124087	0.670374
H	2.679718	-0.566058	1.487208
C	-2.245361	2.929744	-1.003407
H	-1.859596	3.397196	-0.099359
H	-1.880867	3.514968	-1.848559
H	-3.337650	2.956629	-1.006545
C	-2.183669	1.653211	-3.924384
H	-2.590883	1.245958	-4.858074

H	-2.692186	2.603138	-3.737306	H	-4.385314	0.192518	-3.607357
H	-1.125254	1.852473	-4.082406	C	-1.821897	-0.720211	-3.180031
C	-3.956227	0.462522	-2.634428	H	-0.74245	-0.642023	-3.345753
H	-4.213983	-0.330737	-1.928901	H	-2.010175	-1.469448	-2.406284
H	-4.445870	1.385480	-2.308638	H	-2.27423	-1.084558	-4.110212

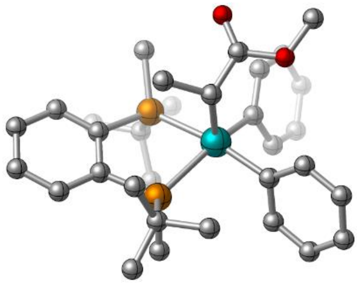

Zero-point correction= 0.697643 (Hartree/Particle)
Thermal correction to Energy= 0.739568
Thermal correction to Enthalpy= 0.740513
Thermal correction to Gibbs Free Energy= 0.625967
Sum of electronic and zero-point Energies= -3342.766417
Sum of electronic and thermal Energies= -3342.724492
Sum of electronic and thermal Enthalpies= -3342.723548
Sum of electronic and thermal Free Energies= -3342.838094

^{2}int8

E(scf) = -2804.65225535 a.u.

ν_{min} = 20.3 cm^{-1}

Fe	4.302426	8.251517	14.196434	C	1.069414	7.626433	11.397199
P	5.445001	7.818099	16.030722	H	3.019266	6.758906	11.543459
P	6.057951	7.725107	13.098350	C	0.187018	8.612376	11.853566
C	7.117969	7.150179	15.592275	H	-0.059010	10.278422	13.202375
C	8.099801	6.760298	16.513880	H	0.745974	6.929763	10.625607
H	7.948860	6.921946	17.576302	H	-0.815307	8.689184	11.439524
C	9.279169	6.153365	16.077051	C	6.940321	9.224757	12.319722
H	10.031137	5.847433	16.799107	C	4.604024	6.401117	16.988336
C	9.483129	5.928281	14.713894	C	5.976193	6.488030	11.728866
H	10.389784	5.436764	14.371928	H	5.287290	6.846698	10.960494
C	8.529275	6.352462	13.786179	H	6.943101	6.278801	11.262418
H	8.713128	6.192540	12.728666	H	5.575710	5.557244	12.141681
C	7.354759	6.988800	14.211808	C	5.843522	9.072154	17.330969
C	2.841211	8.411944	12.931555	H	4.915218	9.445872	17.773231
C	1.910295	9.397443	13.360874	H	6.486498	8.693391	18.130647
C	2.359576	7.536564	11.927035	H	6.350308	9.909820	16.842184
C	0.614440	9.502301	12.842788	C	8.305131	8.913728	11.688864
H	2.207737	10.123722	14.122688	H	8.706772	9.823705	11.224175

H	9.032947	8.576763	12.431510	H	2.668719	7.185206	16.343730
H	8.234869	8.152840	10.904725	H	2.581649	6.097405	17.743405
C	7.116687	10.254962	13.449618	H	3.163714	7.757203	17.948622
H	7.586917	11.167345	13.062233	C	5.281982	5.990844	18.301686
H	6.149891	10.531963	13.886912	H	4.682578	5.215155	18.796210
H	7.748736	9.863631	14.254139	H	6.277637	5.572145	18.133839
C	5.998816	9.796808	11.243512	H	5.371190	6.828384	19.000972
H	5.873893	9.106837	10.402371	C	4.544354	5.196038	16.032403
H	5.006799	10.009466	11.653086	H	4.056194	5.462069	15.086516
H	6.414406	10.731329	10.845766	H	5.543949	4.815980	15.798352
C	3.171923	6.893929	17.273855	H	3.970397	4.380702	16.489799

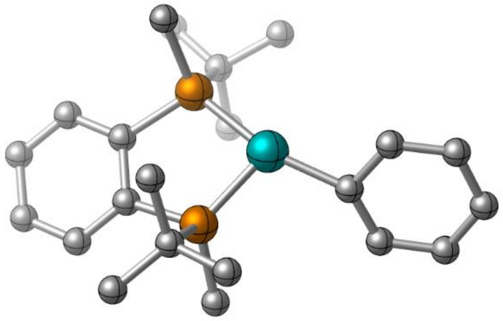

Zero-point correction= 0.494606 (Hartree/Particle)
Thermal correction to Energy= 0.524077
Thermal correction to Enthalpy= 0.525021
Thermal correction to Gibbs Free Energy= 0.434428
Sum of electronic and zero-point Energies= -2804.157650
Sum of electronic and thermal Energies= -2804.128179
Sum of electronic and thermal Enthalpies= -2804.127235
Sum of electronic and thermal Free Energies= -2804.217827

^{2}int-a

E(scf) = -5608.13660507 a.u.

ν_{min} = 30.6 cm^{-1}

C	-2.740359	0.049884	0.431126	H	-5.679591	-2.357099	-0.442928
C	-2.476557	-1.232248	-0.093351	H	-6.141642	-0.163334	0.644276
C	-3.545807	-2.081262	-0.421369	H	-4.288982	1.362928	1.173034
C	-4.861564	-1.692388	-0.181608	P	-0.728693	-1.794633	-0.163777
C	-5.120783	-0.459259	0.421282	P	-1.331950	1.241418	0.591802
C	-4.068359	0.403336	0.721087	C	-0.644358	-2.864255	-1.651145
H	-3.350294	-3.058933	-0.848672	H	-1.216205	-3.787020	-1.522011

H	-1.033010	-2.301626	-2.501607
H	0.400806	-3.111159	-1.845341
C	-1.375632	1.687865	2.379186
H	-0.713139	2.535977	2.562330
H	-2.388922	1.941625	2.699239
H	-1.023542	0.839504	2.970060
C	-1.899469	2.836356	-0.306303
C	-0.537134	-2.964927	1.318836
C	-1.757084	-3.870806	1.544246
H	-1.536820	-4.564017	2.365386
H	-2.649365	-3.302589	1.818731
H	-1.991204	-4.472762	0.660340
C	0.703504	-3.844754	1.073369
H	0.912653	-4.423977	1.980857
H	0.536260	-4.556994	0.259622
H	1.592156	-3.257578	0.839441
C	-0.324025	-2.070606	2.552825
H	0.585441	-1.467056	2.463052
H	-1.175348	-1.399576	2.708891
H	-0.220304	-2.691193	3.450710
C	-0.625463	3.635518	-0.636995
H	-0.912290	4.582259	-1.111361
H	-0.045341	3.870523	0.259998
H	0.016302	3.084282	-1.324036
C	-2.814455	3.722351	0.559408
H	-3.082596	4.611685	-0.023960
H	-3.748851	3.232084	0.841670
H	-2.316783	4.068990	1.469290

C	-2.624612	2.461646	-1.611452
H	-2.006266	1.811643	-2.233953
H	-3.581171	1.966250	-1.425104
H	-2.827382	3.379438	-2.176910
Fe	0.734217	0.107065	-0.122342
C	2.363694	-0.989149	-0.242894
C	3.137313	-1.184138	0.920721
C	2.759862	-1.700464	-1.390478
C	4.222090	-2.064998	0.948581
H	2.895959	-0.637522	1.828939
C	3.845460	-2.582668	-1.372964
H	2.206067	-1.560622	-2.313360
C	4.578640	-2.776196	-0.200293
H	4.791679	-2.192040	1.866557
H	4.117365	-3.120068	-2.279001
H	5.421913	-3.461634	-0.183948
Br	0.370002	0.535641	-2.488431
C	1.781633	1.637156	0.523489
C	2.524025	2.455445	-0.347838
C	1.940486	1.863022	1.902070
C	3.368415	3.458626	0.132276
H	2.433848	2.301593	-1.419214
C	2.781631	2.868735	2.394052
H	1.401698	1.246811	2.619696
C	3.497777	3.675564	1.508140
H	3.928330	4.073751	-0.568986
H	2.874620	3.020650	3.467231
H	4.150643	4.459576	1.882630

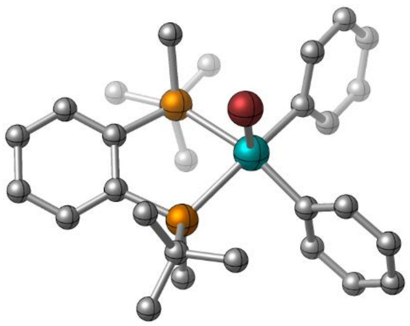

Zero-point correction= 0.589048 (Hartree/Particle)
Thermal correction to Energy= 0.625339
Thermal correction to Enthalpy= 0.626283
Thermal correction to Gibbs Free Energy= 0.522915

Sum of electronic and zero-point Energies= -5607.547558
Sum of electronic and thermal Energies= -5607.511267
Sum of electronic and thermal Enthalpies= -5607.510322
Sum of electronic and thermal Free Energies= -5607.613691

^{2}int-b
E(scf) = -5683.59867271 a.u.
ν_{min} = 30.0 cm^{-1}

C	2.953186	2.685322	-1.189611	H	2.921820	-0.613818	0.311542
H	3.365358	2.828334	-0.189887	C	2.723257	2.061022	3.101000
H	3.270467	1.705054	-1.553833	H	1.005467	2.809418	2.069928
H	3.392448	3.449911	-1.847060	C	3.745876	1.108789	3.127779
C	-2.110013	-1.167773	-0.140753	H	4.610260	-0.590484	2.116777
C	-2.715055	-2.431009	-0.050846	H	2.671616	2.826658	3.871640
C	-2.805585	-0.106604	-0.753798	H	4.488716	1.123042	3.920920
C	-4.148566	-0.294542	-1.118233	Br	1.235220	-0.414210	-2.098148
C	-4.023751	-2.623163	-0.488509	C	-2.219690	-1.521013	2.939762
H	-4.480258	-3.605853	-0.415738	H	-2.438220	-1.382468	4.005794
C	-4.756827	-1.540970	-0.981748	H	-3.150464	-1.370822	2.388067
H	-5.793036	-1.671936	-1.279330	H	-1.903098	-2.559881	2.803333
H	-2.176250	-3.262324	0.390565	C	-1.688920	0.906965	2.636003
H	-4.721295	0.527431	-1.531365	H	-0.936368	1.660486	2.388346
P	-0.541679	-0.754471	0.725405	H	-2.549934	1.041166	1.973591
P	-1.844938	1.401730	-1.221650	H	-2.026554	1.097938	3.661608
Fe	0.475775	1.126342	-0.374029	C	0.077006	-0.707134	3.473760
C	-1.126200	-0.519643	2.529068	H	0.427958	-1.743586	3.473782
C	-2.127760	1.539365	-3.119522	H	0.916258	-0.059693	3.225545
C	0.958827	4.021297	-0.534509	H	-0.238896	-0.468325	4.496650
O	1.492517	4.615776	0.392081	C	-3.462657	2.223576	-3.465851
O	-0.206455	4.497890	-1.087831	H	-3.509797	3.252542	-3.098235
C	-0.726785	5.691489	-0.485727	H	-3.557148	2.263090	-4.557932
H	-1.606203	5.966232	-1.070648	H	-4.332877	1.682074	-3.089315
H	-1.011108	5.518366	0.556915	C	-2.077918	0.136149	-3.751585
H	0.008075	6.500697	-0.518630	H	-1.162194	-0.391014	-3.472804
C	1.427316	2.779538	-1.181267	H	-2.936734	-0.478542	-3.472080
H	1.049672	2.759953	-2.202748	H	-2.085334	0.244923	-4.842988
C	1.800028	1.061416	1.069979	C	-0.982419	2.385459	-3.703867
C	2.850586	0.129206	1.100031	H	-1.176401	2.560969	-4.769005
C	1.768552	2.037442	2.080199	H	-0.894255	3.357534	-3.210754
C	3.810695	0.147037	2.116325	H	-0.029308	1.858671	-3.619742

C	0.397461	-2.329741	0.749343	C	-2.811680	2.794749	-0.501004
H	1.396825	-2.139625	1.142252	H	-3.887701	2.639685	-0.611506
H	-0.095143	-3.079728	1.373078	H	-2.570787	2.882355	0.560928
H	0.487736	-2.697124	-0.273576	H	-2.522977	3.720016	-0.997172

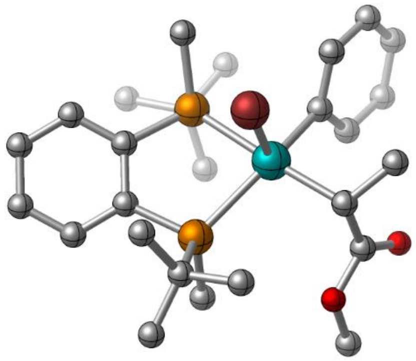

Zero-point correction= 0.608566 (Hartree/Particle)
Thermal correction to Energy= 0.646852
Thermal correction to Enthalpy= 0.647796
Thermal correction to Gibbs Free Energy= 0.541217
Sum of electronic and zero-point Energies= -5682.990107
Sum of electronic and thermal Energies= -5682.951820
Sum of electronic and thermal Enthalpies= -5682.950876
Sum of electronic and thermal Free Energies= -5683.057456

^{2}int-b-E
E(scf) = -5683.59798257 a.u.
ν_{min} = 33.5 cm^{-1}

C	-2.421533	-0.406225	0.469665	C	-0.796630	-2.222753	-0.982516
C	-3.316335	-1.180834	1.221154	C	0.063749	-1.740400	1.744422
C	-2.891666	0.754257	-0.181713	C	-2.148380	1.567597	-2.983099
C	-4.242051	1.114545	-0.053661	C	-2.182161	3.504224	-0.784355
C	-4.655707	-0.812555	1.339713	C	1.522125	2.927878	-1.081134
H	-5.331985	-1.420364	1.933435	C	0.306062	1.815183	1.285608
C	-5.119553	0.337469	0.700904	C	1.571183	1.810771	1.906951
H	-6.159975	0.634439	0.792891	C	-0.779272	2.233667	2.071107
H	-2.971782	-2.073761	1.727250	C	1.745144	2.193083	3.238094
H	-4.619812	2.010781	-0.531941	H	2.452414	1.498253	1.349513
P	-0.644435	-0.828194	0.217873	C	-0.615469	2.639913	3.399383
P	-1.685494	1.771168	-1.150150	H	-1.786170	2.230106	1.669851
Fe	0.405094	1.214657	-0.582894	C	0.647998	2.620857	3.990911

H	2.737184	2.167926	3.682382	C	-1.074183	2.302593	-3.804416
H	-1.481599	2.959808	3.973916	H	-0.951298	3.347351	-3.506233
H	0.777219	2.930397	5.024459	H	-1.356483	2.282278	-4.863717
C	0.890678	4.228445	-0.738627	H	-0.107389	1.804930	-3.703641
O	0.802825	4.744362	0.365725	C	-3.539438	2.154016	-3.281932
O	0.394834	4.873848	-1.844813	H	-3.740244	2.038467	-4.353756
C	-0.130563	6.186544	-1.607744	H	-3.608120	3.220472	-3.051207
C	-0.377647	-3.217895	1.824827	H	-4.331911	1.626366	-2.744535
H	-1.459904	-3.345612	1.903842	H	-3.255237	3.589344	-0.609614
H	0.066458	-3.656006	2.726680	H	-1.910785	4.130390	-1.631613
H	-0.026709	-3.807457	0.975140	H	-1.644756	3.858540	0.094688
C	1.594082	-1.715044	1.550731	H	-1.119787	-1.843007	-1.946993
H	2.070800	-2.251237	2.380182	H	0.172938	-2.704149	-1.114157
H	1.984033	-0.696562	1.539975	H	-1.528907	-2.949525	-0.623169
H	1.895562	-2.203164	0.618635	H	-0.475345	6.549053	-2.577853
C	-0.311997	-1.039005	3.060046	H	0.644122	6.851683	-1.214441
H	-1.394354	-0.999549	3.211544	H	-0.963026	6.165928	-0.898616
H	0.077249	-0.025494	3.113446	Br	1.595973	-0.191109	-2.132995
H	0.119306	-1.605904	3.894171	C	2.225113	2.991977	-2.446302
C	-2.133132	0.080041	-3.358652	H	1.525671	2.981671	-3.281981
H	-2.846121	-0.500280	-2.764721	H	2.814406	3.915904	-2.532657
H	-1.132870	-0.342511	-3.238802	H	2.898871	2.144694	-2.567848
H	-2.412153	-0.026376	-4.413498	H	2.290752	2.776819	-0.315970

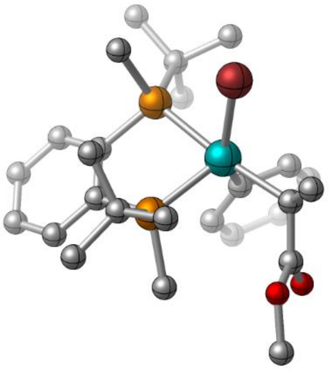

Zero-point correction= 0.610264 (Hartree/Particle)
Thermal correction to Energy= 0.648065
Thermal correction to Enthalpy= 0.649009
Thermal correction to Gibbs Free Energy= 0.544334
Sum of electronic and zero-point Energies= -5682.987719
Sum of electronic and thermal Energies= -5682.949918
Sum of electronic and thermal Enthalpies= -5682.948974
Sum of electronic and thermal Free Energies= -5683.053649

^{2}TS2
E(scf) = -5608.11525572 a.u.
ν_{min} = -297.8 cm^{-1}

C	-2.126316	-0.983413	-0.381117	C	1.683535	5.649892	-1.071593
C	-2.778334	-2.225045	-0.458244	H	3.332704	4.760243	-2.141753
C	-2.671880	0.127254	-1.067771	H	-0.008079	6.218131	0.147436
C	-3.904305	-0.020755	-1.724769	H	1.846387	6.647342	-1.469015
C	-3.978441	-2.364404	-1.152320	H	-3.778093	2.879857	-0.626394
H	-4.466770	-3.333276	-1.201674	H	-2.341613	3.916697	-0.426862
C	-4.557005	-1.251334	-1.765086	H	-2.722916	2.687440	0.791211
H	-5.505576	-1.343542	-2.286041	Br	1.504043	-0.008367	-2.017370
H	-2.352152	-3.090706	0.037788	C	-0.498076	3.215498	-3.122121
H	-4.357385	0.828041	-2.223103	H	-0.471656	3.626021	-4.139068
P	-0.634816	-0.744266	0.683801	H	-0.544994	4.056386	-2.423515
P	-1.683902	1.692324	-1.167630	H	0.437067	2.680751	-2.944386
Fe	0.429302	1.210710	-0.180214	C	-2.984258	3.059247	-3.367977
C	0.307194	-2.313525	0.505546	H	-2.899444	3.370693	-4.416558
C	-1.307925	-0.781552	2.453550	H	-3.897729	2.465341	-3.282220
C	-1.715869	2.277832	-2.983007	H	-3.108774	3.966194	-2.769212
C	-2.742828	2.911667	-0.277547	C	-1.534020	1.070033	-3.918564
C	1.673593	1.796398	1.301648	H	-1.397535	1.432443	-4.945069
C	2.886011	1.069313	1.182091	H	-0.649088	0.490076	-3.644507
C	1.340710	2.268046	2.591740	H	-2.403275	0.406803	-3.911288
C	3.669147	0.778124	2.296862	H	1.278523	-2.180317	0.988951
H	3.202250	0.710628	0.208356	H	-0.199125	-3.177881	0.942957
C	2.136260	1.990321	3.701196	H	0.481788	-2.482881	-0.558286
H	0.451916	2.874125	2.730836	C	-0.084906	-0.930011	3.378928
C	3.303850	1.233157	3.568127	H	-0.390992	-0.759177	4.418254
H	4.578870	0.196650	2.167613	H	0.338814	-1.937592	3.321554
H	1.838919	2.366944	4.676791	H	0.703208	-0.212839	3.142580
H	3.924308	1.016435	4.432593	C	-2.005530	0.568940	2.685826
C	1.246610	3.032538	-0.046267	H	-1.317487	1.406156	2.539662
C	2.314364	3.311374	-0.929821	H	-2.854217	0.696314	2.005558
C	0.441509	4.129172	0.347830	H	-2.387483	0.622497	3.712381
C	2.514933	4.590466	-1.445634	C	-2.297871	-1.921178	2.735539
H	2.974301	2.507349	-1.233818	H	-2.567273	-1.904888	3.799211
C	0.646687	5.406919	-0.161863	H	-3.220300	-1.817400	2.158257
H	-0.372359	3.968777	1.048001	H	-1.868076	-2.905422	2.523428

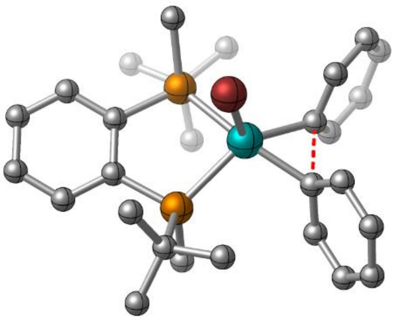

Zero-point correction= 0.587522 (Hartree/Particle)
Thermal correction to Energy= 0.623454
Thermal correction to Enthalpy= 0.624398
Thermal correction to Gibbs Free Energy= 0.522118
Sum of electronic and zero-point Energies= -5607.527734
Sum of electronic and thermal Energies= -5607.491802
Sum of electronic and thermal Enthalpies= -5607.490857
Sum of electronic and thermal Free Energies= -5607.593137

^{2}TS7

E(scf) = -3343.44137104 a.u.

ν_{min} = -155.7 cm^{-1}

C	0.878270	3.667551	-3.426461
H	0.374530	4.083192	-2.547394
H	1.851560	4.169380	-3.494416
H	0.288152	3.937305	-4.305954
C	-2.203561	-1.137257	-0.036483
C	-2.979966	-2.271564	0.249983
C	-2.787352	0.141801	0.100100
C	-4.054791	0.262382	0.689819
C	-4.260551	-2.141285	0.786975
H	-4.843730	-3.028937	1.014101
C	-4.778367	-0.872033	1.055272
H	-5.758607	-0.765902	1.510850
H	-2.581722	-3.263214	0.069492
H	-4.497263	1.243163	0.830109
P	-0.403592	-1.198899	-0.463351
P	-1.964954	1.531194	-0.792792
Fe	0.373880	1.065667	-0.862548
C	-0.284813	-2.440472	-1.817163
H	0.754212	-2.510415	-2.145630
H	-0.629635	-3.428679	-1.499410
H	-0.893983	-2.101545	-2.657654
C	0.406260	-2.051035	1.049897
C	-2.971595	1.533150	-2.416221
C	-2.553626	3.052868	0.062549
H	-1.992288	3.904141	-0.327824
H	-3.621572	3.229450	-0.084384
H	-2.346645	2.977480	1.130519
C	0.440014	1.371063	-4.390865
O	-0.323193	1.779665	-5.261338
O	0.802759	0.051134	-4.332485
C	0.265449	-0.791337	-5.361006
H	0.650355	-1.790946	-5.155100
H	-0.827614	-0.797035	-5.339471
H	0.595867	-0.459100	-6.349776
C	1.058265	2.178056	-3.348295
H	1.983113	1.794862	-2.941538
C	0.742126	2.676490	0.229119
C	1.603916	3.740725	-0.102223
C	0.292233	2.646837	1.567092
C	1.963876	4.728083	0.820733

H	2.037142	3.792090	-1.095219	H	0.949166	-1.423232	3.054170
C	0.659929	3.616238	2.507235	C	1.812917	-2.535974	0.649717
H	-0.366631	1.845507	1.896390	H	2.310474	-2.933913	1.542526
C	1.493712	4.673083	2.135564	H	1.767177	-3.345517	-0.085878
H	2.629287	5.533189	0.515205	H	2.434623	-1.738857	0.250134
H	0.289280	3.546754	3.528067	C	-0.369937	-3.262531	1.591775
H	1.777508	5.434698	2.857192	H	0.232251	-3.734596	2.377788
C	2.292212	0.527264	-0.800460	H	-1.325362	-2.981512	2.039059
C	3.169811	0.808291	0.269795	H	-0.550964	-4.020646	0.822765
C	2.832346	-0.288474	-1.823875	C	-2.558865	0.279038	-3.203566
C	4.471924	0.298161	0.333088	H	-2.988185	0.313964	-4.210900
H	2.828657	1.434826	1.088525	H	-1.476591	0.197639	-3.301004
C	4.135490	-0.793410	-1.781919	H	-2.922681	-0.629354	-2.713611
H	2.214650	-0.540610	-2.680519	C	-4.496015	1.514406	-2.218567
C	4.965222	-0.509349	-0.693704	H	-4.974583	1.583569	-3.203531
H	5.103104	0.536101	1.187222	H	-4.844836	0.592819	-1.747663
H	4.500534	-1.416832	-2.596170	H	-4.854071	2.361894	-1.626227
H	5.976919	-0.904803	-0.649924	C	-2.591914	2.816867	-3.171827
C	0.506024	-0.983291	2.152490	H	-3.063991	2.812659	-4.16074
H	1.134556	-0.144940	1.844930	H	-2.93769	3.710384	-2.642011
H	-0.482398	-0.595340	2.423416	H	-1.520409	2.896595	-3.32816

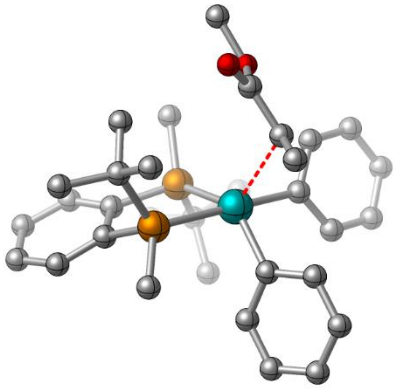

Zero-point correction= 0.695122 (Hartree/Particle)
Thermal correction to Energy= 0.736954
Thermal correction to Enthalpy= 0.737898
Thermal correction to Gibbs Free Energy= 0.623865
Sum of electronic and zero-point Energies= -3342.746249
Sum of electronic and thermal Energies= -3342.704417
Sum of electronic and thermal Enthalpies= -3342.703473
Sum of electronic and thermal Free Energies= -3342.817506

^{2}TS8
E(scf) = -3343.43678331 a.u.
ν_{min} = -316.4 cm^{-1}

C	3.006047	2.579766	-1.681628	C	1.715629	-0.274543	-1.304778
H	3.676614	3.034661	-0.949996	C	2.700192	-1.030147	-0.639308
H	3.270836	1.530423	-1.797023	C	1.659838	-0.442995	-2.707560
H	3.186002	3.081174	-2.642120	C	3.568682	-1.893702	-1.314380
C	-2.549307	-0.864088	-0.139413	H	2.792578	-0.948711	0.439412
C	-3.405716	-1.959194	0.059199	C	2.520001	-1.305809	-3.395773
C	-3.099503	0.383216	-0.499469	H	0.940464	0.122419	-3.296046
C	-4.495719	0.532152	-0.522897	C	3.483436	-2.041067	-2.701293
C	-4.789464	-1.808210	-0.011919	H	4.312789	-2.455960	-0.753424
H	-5.436425	-2.665552	0.149187	H	2.440539	-1.396496	-4.477365
C	-5.336547	-0.550238	-0.269116	H	4.155888	-2.711430	-3.229992
H	-6.413851	-0.415063	-0.294701	C	-0.244833	-2.566567	-0.515391
H	-2.998727	-2.939197	0.279324	H	-0.945423	-3.360303	-0.250408
H	-4.943052	1.491779	-0.755489	H	-0.199562	-2.474174	-1.602725
P	-0.729658	-0.937306	0.188898	H	0.752124	-2.831472	-0.161026
P	-1.933704	1.680188	-1.119090	C	-2.747365	3.258548	-0.624326
Fe	0.354285	0.936590	-0.559977	H	-2.399409	4.049733	-1.285349
C	-0.671149	-1.200075	2.082945	H	-3.835483	3.202891	-0.685429
C	-2.249689	1.592663	-3.005976	H	-2.464832	3.509327	0.400395
C	1.271928	4.155153	-0.913764	C	-1.470676	-0.062732	2.740575
O	2.105418	4.983346	-0.576799	H	-2.538363	-0.131485	2.510429
O	-0.055207	4.475355	-0.968993	H	-1.358026	-0.122825	3.829884
C	-0.368878	5.803021	-0.521303	H	-1.108408	0.919054	2.426919
H	-1.450146	5.909827	-0.612294	C	0.804110	-1.120909	2.510701
H	-0.067030	5.941791	0.520696	H	1.250978	-0.160150	2.254980
H	0.132616	6.554478	-1.137356	H	0.872656	-1.247405	3.598024
C	1.557070	2.752747	-1.260152	H	1.400481	-1.913369	2.047307
H	0.852320	2.382499	-2.008713	C	-1.243710	-2.553155	2.535416
C	1.429438	1.945734	0.680765	H	-2.310636	-2.648787	2.322931
C	2.715660	1.682607	1.215764	H	-0.718044	-3.400831	2.085783
C	0.536559	2.674432	1.516966	H	-1.123459	-2.635113	3.622955
C	3.040971	2.017597	2.527187	C	-1.192760	2.473166	-3.699059
H	3.454306	1.182113	0.602935	H	-1.431493	2.556106	-4.765890
C	0.858245	2.976045	2.841589	H	-1.153135	3.484055	-3.281561
H	-0.409025	3.028377	1.119408	H	-0.192024	2.040486	-3.624654
C	2.109968	2.645120	3.365934	C	-2.086439	0.129247	-3.454242
H	4.031732	1.773377	2.904859	H	-2.907319	-0.497984	-3.095486
H	0.130525	3.501080	3.456749	H	-2.081049	0.082118	-4.549773
H	2.368111	2.899077	4.389796	H	-1.149125	-0.309493	-3.099342

C	-3.648715	2.091277	-3.403153	H	-4.445369	1.502483	-2.942731
H	-3.762182	1.995846	-4.490152	H	-3.80154	3.144731	-3.150851

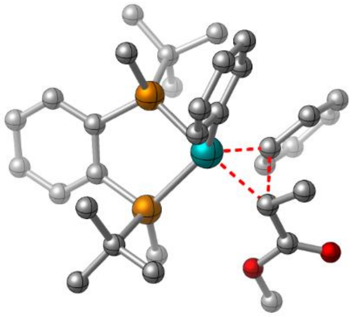

Zero-point correction= 0.694820 (Hartree/Particle)
Thermal correction to Energy= 0.736598
Thermal correction to Enthalpy= 0.737542
Thermal correction to Gibbs Free Energy= 0.622572
Sum of electronic and zero-point Energies= -3342.741963
Sum of electronic and thermal Energies= -3342.700185
Sum of electronic and thermal Enthalpies= -3342.699241
Sum of electronic and thermal Free Energies= -3342.814211

^{2}TS-b

E(scf) = -5683.56175317 a.u.

ν_{min} = -214.8 cm^{-1}

C	-2.553960	-0.510942	0.315629	C	-2.004595	1.124604	-3.238153
C	-3.531422	-1.216280	1.030791	C	-2.067871	3.288353	-1.268698
C	-2.931362	0.624012	-0.440423	C	1.524793	3.399792	-1.863441
C	-4.272537	1.034585	-0.430022	H	0.539503	3.424193	-2.307074
C	-4.862103	-0.798772	1.026475	C	0.242584	1.858190	1.008959
H	-5.604417	-1.352927	1.593409	C	1.534369	1.949496	1.575591
C	-5.231960	0.333037	0.299869	C	-0.807769	2.391945	1.782935
H	-6.264093	0.670846	0.299268	C	1.760241	2.493517	2.843150
H	-3.258717	-2.095901	1.601027	H	2.400676	1.586702	1.019771
H	-4.579895	1.910575	-0.990189	C	-0.595680	2.951990	3.045845
P	-0.765784	-0.967531	0.295110	H	-1.830588	2.336728	1.420450
P	-1.618217	1.505978	-1.411747	C	0.690995	2.996107	3.590330
Fe	0.315931	0.825384	-0.625817	H	2.771258	2.533662	3.242003
C	-0.745641	-2.473489	-0.769135	H	-1.438919	3.340388	3.613143
C	-0.208075	-1.668900	1.975044	H	0.859336	3.421109	4.576252

C	1.746075	4.208211	-0.680377
O	2.832987	4.372141	-0.135530
O	0.606200	4.807585	-0.245572
C	0.739085	5.591765	0.948680
C	2.699970	2.993366	-2.681104
H	2.422281	2.295212	-3.470186
H	3.478556	2.538869	-2.062548
H	3.149674	3.884008	-3.151187
C	-0.676300	-3.114179	2.236330
H	-1.763844	-3.209065	2.288898
H	-0.277758	-3.435954	3.206240
H	-0.306681	-3.817576	1.486299
C	1.334368	-1.662940	1.897384
H	1.744747	-2.083746	2.823202
H	1.725933	-0.649329	1.785241
H	1.706993	-2.266993	1.063452
C	-0.670117	-0.767566	3.129931
H	-1.760819	-0.695372	3.183127
H	-0.265254	0.239551	3.046051
H	-0.317729	-1.193986	4.077112
C	-1.923767	-0.392896	-3.458932
H	-2.629526	-0.933763	-2.820451
H	-0.913061	-0.762014	-3.268624
H	-2.171776	-0.623840	-4.501791
C	-0.923131	1.812614	-4.094236
H	-0.973678	2.904233	-4.032878
H	-1.071897	1.539924	-5.146022
H	0.080751	1.490865	-3.804094
C	-3.395669	1.626501	-3.660336
H	-3.530262	1.438515	-4.732723
H	-3.521208	2.701197	-3.499184
H	-4.196576	1.099754	-3.134759
H	-3.146478	3.443777	-1.206449
H	-1.693666	3.831712	-2.138287
H	-1.584792	3.704181	-0.386687
H	-0.941811	-2.196432	-1.801765
H	0.244655	-2.931728	-0.726288
H	-1.500521	-3.193480	-0.443369
H	-0.272319	5.899411	1.216969
H	1.361237	6.473993	0.764813
H	1.179823	5.002454	1.754043
Br	1.761876	-0.276884	-2.126247

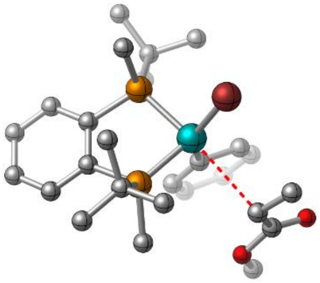

Zero-point correction= 0.605393 (Hartree/Particle)
Thermal correction to Energy= 0.644219
Thermal correction to Enthalpy= 0.645163
Thermal correction to Gibbs Free Energy= 0.536135
Sum of electronic and zero-point Energies= -5682.956360
Sum of electronic and thermal Energies= -5682.917534
Sum of electronic and thermal Enthalpies= -5682.916590
Sum of electronic and thermal Free Energies= -5683.025618

[2]TS-b-E

E(scf) = -5683.55900274 a.u.
ν_{min} = -296.1 cm^{-1}

C	-2.516878	-0.517422	0.438555	H	-0.275606	-3.946486	1.149301
C	-3.467388	-1.282460	1.128704	C	1.423754	-1.877022	1.697605
C	-2.915608	0.688416	-0.183858	H	1.866500	-2.387235	2.561430
C	-4.250986	1.103520	-0.072539	H	1.832935	-0.865484	1.657066
C	-4.792536	-0.858972	1.225394	H	1.742906	-2.410794	0.796382
H	-5.513527	-1.460583	1.771022	C	-0.501628	-1.074397	3.109738
C	-5.183840	0.339087	0.627734	H	-1.587009	-0.993939	3.223839
H	-6.211623	0.680974	0.706600	H	-0.083351	-0.068789	3.105979
H	-3.178276	-2.212606	1.602418	H	-0.111592	-1.598302	3.990869
H	-4.574229	2.033199	-0.526988	C	-2.047797	0.000791	-3.340933
P	-0.738714	-0.984746	0.280536	H	-2.752707	-0.595597	-2.752809
P	-1.636355	1.662361	-1.112022	H	-1.041541	-0.406616	-3.214655
Fe	0.324801	0.876833	-0.513958	H	-2.318652	-0.107558	-4.397914
C	-0.792862	-2.368910	-0.936802	C	-1.015372	2.244812	-3.750062
C	-0.112592	-1.861888	1.849036	H	-0.988075	3.311141	-3.507109
C	-2.091255	1.485003	-2.951400	H	-1.230498	2.154546	-4.821770
C	-2.073208	3.416429	-0.769075	H	-0.023347	1.824629	-3.568336
C	1.696809	3.492707	-1.272693	C	-3.482641	2.055754	-3.271424
C	0.368700	1.722539	1.219201	H	-3.663144	1.964007	-4.349558
C	1.690245	1.737347	1.721865	H	-3.570800	3.115555	-3.015805
C	-0.624955	2.223751	2.080723	H	-4.277903	1.506272	-2.760329
C	2.001579	2.217997	2.995842	H	-3.150194	3.571417	-0.685304
H	2.512410	1.357067	1.111521	H	-1.695539	4.027864	-1.587683
C	-0.327605	2.714347	3.355483	H	-1.587777	3.738912	0.150954
H	-1.667753	2.203053	1.776489	H	-1.040313	-1.982047	-1.922118
C	0.989013	2.714720	3.821866	H	0.192063	-2.836785	-0.995772
H	3.032480	2.207032	3.342613	H	-1.539455	-3.113442	-0.649503
H	-1.128460	3.087708	3.990198	H	-0.720326	6.946771	-2.414388
H	1.223168	3.092383	4.813589	H	0.267408	7.202872	-0.942411
C	0.894164	4.611941	-0.819709	H	-1.228475	6.247249	-0.846321
O	0.711759	4.939870	0.348002	Br	1.752875	-0.074738	-2.132394
O	0.358300	5.324862	-1.859574	C	2.229576	3.429000	-2.664746
C	-0.374225	6.499397	-1.481416	H	1.457864	3.629652	-3.410477
C	-0.600506	-3.318276	1.982252	H	3.006476	4.199815	-2.801688
H	-1.686688	-3.400302	2.068978	H	2.674877	2.455311	-2.868531
H	-0.170278	-3.744221	2.896813	H	2.232902	2.988428	-0.479380

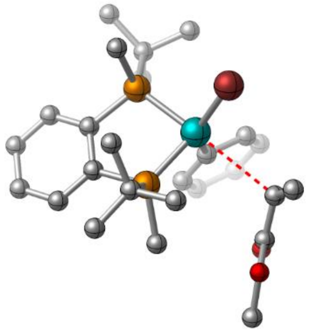

Zero-point correction= 0.606097 (Hartree/Particle)
Thermal correction to Energy= 0.644565
Thermal correction to Enthalpy= 0.645509
Thermal correction to Gibbs Free Energy= 0.537835
Sum of electronic and zero-point Energies= -5682.952905
Sum of electronic and thermal Energies= -5682.914438
Sum of electronic and thermal Enthalpies= -5682.913494
Sum of electronic and thermal Free Energies= -5683.021167

^{2}TS-c

E(scf) = -5683.57051468 a.u.

ν_{min} = -313.9 cm^{-1}

C	3.379801	2.512147	-0.746661	H	-0.534737	-2.462042	-1.594987
H	3.695855	1.469620	-0.726501	C	-0.410209	-1.528723	2.203830
H	3.857088	2.985549	-1.615168	C	-1.750442	1.238769	-2.958822
H	3.733625	3.022579	0.151079	C	-2.267370	3.271433	-0.834713
C	-2.497248	-0.736179	0.187644	H	-1.527368	4.011444	-1.126310
C	-3.494681	-1.655797	0.550325	H	-3.181394	3.439228	-1.407891
C	-2.873694	0.516139	-0.342223	H	-2.495642	3.394321	0.226893
C	-4.235407	0.858194	-0.387607	C	1.464877	4.045733	-0.847344
C	-4.843381	-1.313471	0.475284	O	1.906548	4.892365	-0.090098
H	-5.601556	-2.033993	0.767579	O	0.572736	4.368124	-1.827703
C	-5.212998	-0.042230	0.032148	C	0.220901	5.761026	-1.903621
H	-6.260948	0.239885	-0.009796	H	-0.503907	5.841476	-2.714920
H	-3.221958	-2.648005	0.889085	H	-0.220187	6.109863	-0.965710
H	-4.544250	1.824962	-0.769220	H	1.102039	6.369138	-2.125974
P	-0.693923	-1.068302	0.382322	C	1.862569	2.611545	-0.878287
P	-1.563949	1.589599	-1.087966	H	1.536484	2.177359	-1.825205
Fe	0.523109	0.855648	-0.299469	C	1.243257	1.991481	1.047240
C	-0.461802	-2.652096	-0.523883	C	2.306815	1.483402	1.829982
H	0.533531	-3.047322	-0.319867	C	0.352526	2.894068	1.671065
H	-1.219525	-3.385463	-0.236298	C	2.401539	1.777805	3.188987

H	3.045940	0.834000	1.374373	H	-0.378193	-0.467584	-2.860096
C	0.449907	3.182809	3.033372	H	-2.091931	-0.910791	-2.686789
H	-0.411742	3.388384	1.085055	H	-1.446717	-0.455935	-4.270149
C	1.468771	2.619835	3.805302	C	1.102533	-1.785082	2.345801
H	3.209951	1.342635	3.771400	H	1.340295	-1.949388	3.403717
H	-0.266666	3.863642	3.486254	H	1.423721	-2.671683	1.790705
H	1.549506	2.849129	4.863795	H	1.689434	-0.934274	1.999650
C	-0.747103	2.130175	-3.711025	C	-1.172528	-2.776118	2.679977
H	-0.857265	1.958634	-4.788665	H	-0.831335	-3.026051	3.692272
H	-0.907196	3.193223	-3.517516	H	-2.249567	-2.605625	2.738980
H	0.285916	1.891304	-3.445288	H	-0.987280	-3.650834	2.049458
C	-3.168593	1.514442	-3.481106	C	-0.822763	-0.319642	3.057555
H	-3.198456	1.290705	-4.554482	H	-0.261624	0.574476	2.786255
H	-3.917921	0.884751	-2.994773	H	-1.891220	-0.101178	2.954099
H	-3.461815	2.561604	-3.360640	H	-0.624812	-0.532034	4.114844
C	-1.394466	-0.239118	-3.196504	Br	2.185358	-0.634312	-1.352742

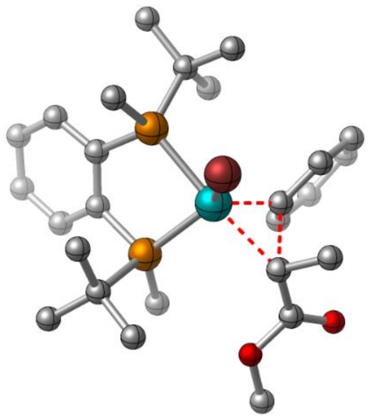

Zero-point correction= 0.607386 (Hartree/Particle)
Thermal correction to Energy= 0.645446
Thermal correction to Enthalpy= 0.646390
Thermal correction to Gibbs Free Energy= 0.539507
Sum of electronic and zero-point Energies= -5682.963129
Sum of electronic and thermal Energies= -5682.925069
Sum of electronic and thermal Enthalpies= -5682.924125
Sum of electronic and thermal Free Energies= -5683.031007

^{2}TS-c-E

E(scf) = -5683.56357846 a.u.

ν_{min} = -317.0 cm^{-1}

C	-2.485235	-0.897631	0.041462	C	-2.996616	0.256544	-0.589183
C	-3.367744	-1.928659	0.402621	C	-4.386264	0.396553	-0.738708

C	-4.742851	-1.787618	0.226012
H	-5.411187	-2.591859	0.519282
C	-5.255172	-0.609553	-0.320284
H	-6.326859	-0.482978	-0.443060
H	-2.984186	-2.851012	0.821868
H	-4.800433	1.285834	-1.199862
P	-0.669068	-0.951988	0.364264
P	-1.798476	1.463344	-1.318704
Fe	0.308386	1.071407	-0.359196
C	-0.153635	-2.541904	-0.402492
H	0.858234	-2.789405	-0.081079
H	-0.837826	-3.350299	-0.131888
H	-0.142526	-2.426534	-1.486602
C	-0.455718	-1.245568	2.230418
C	-1.786479	0.981826	-3.168968
C	-2.768245	3.033402	-1.232154
H	-2.151686	3.888841	-1.502580
H	-3.642414	3.021538	-1.886622
H	-3.109600	3.166039	-0.202259
C	2.743213	3.425009	-0.491504
O	3.778303	2.796628	-0.617566
O	2.706501	4.624581	0.152191
C	3.946097	5.042118	0.738871
H	3.746087	6.010188	1.200995
H	4.275016	4.326319	1.498253
H	4.730184	5.141389	-0.017610
C	1.390880	3.026925	-0.983357
C	0.777784	2.394791	0.921394
C	1.851717	2.086875	1.798177
C	-0.269589	3.192324	1.433805
C	1.818246	2.473474	3.133685
H	2.707958	1.544272	1.413342
C	-0.295445	3.575664	2.778620
H	-1.071036	3.513817	0.781416
C	0.740616	3.213801	3.640099
H	2.641493	2.195607	3.787496
H	-1.130829	4.166581	3.146639
H	0.722483	3.516773	4.682851
C	-0.908463	2.005910	-3.909117
H	-0.842615	1.725894	-4.967319
H	-1.327080	3.016382	-3.862717
H	0.110441	2.028864	-3.511505
C	-3.186243	0.951862	-3.800225
H	-3.090317	0.678266	-4.858161
H	-3.834934	0.210242	-3.327087
H	-3.685742	1.924527	-3.762594
C	-1.146268	-0.412854	-3.282611
H	-0.130397	-0.427273	-2.874690
H	-1.740855	-1.171477	-2.764347
H	-1.087296	-0.700132	-4.339176
C	1.062532	-1.268027	2.491420
H	1.238503	-1.325192	3.572486
H	1.547415	-2.132662	2.028350
H	1.548553	-0.366031	2.119826
C	-1.068140	-2.557100	2.748607
H	-0.774711	-2.686499	3.797843
H	-2.159819	-2.543233	2.721972
H	-0.712249	-3.435260	2.201463
C	-1.096630	-0.055899	2.962053
H	-0.645479	0.889508	2.660660
H	-2.174194	-0.000282	2.772886
H	-0.951017	-0.167588	4.043021
Br	2.265574	-0.206508	-1.093690
C	0.557619	4.222028	-1.419060
H	0.160228	4.788103	-0.573555
H	1.171257	4.917576	-2.005187
H	-0.267148	3.912488	-2.054705
H	1.552340	2.328616	-1.804941

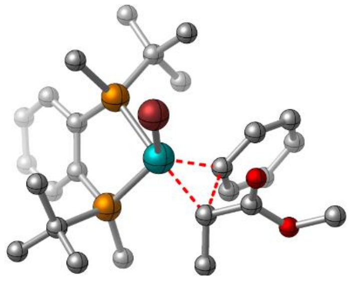

Zero-point correction= 0.606920 (Hartree/Particle)
Thermal correction to Energy= 0.645142
Thermal correction to Enthalpy= 0.646087
Thermal correction to Gibbs Free Energy= 0.539035
Sum of electronic and zero-point Energies= -5682.956659
Sum of electronic and thermal Energies= -5682.918436
Sum of electronic and thermal Enthalpies= -5682.917492
Sum of electronic and thermal Free Energies= -5683.024543

^{3}1

E(scf) = -7716.61792528 a.u.

ν_{min} = 21.6 cm^{-1}

Br	9.674140	6.304259	14.466647	H	3.275713	7.055757	12.616790
Br	8.882657	9.834523	13.245240	H	3.908836	6.402531	11.103598
Fe	7.958496	7.971937	14.427677	H	3.929997	8.147564	11.376023
P	5.844297	8.326318	13.781180	C	5.365839	9.943804	13.072654
P	7.029625	7.374880	16.376930	H	5.594398	10.713803	13.813926
C	4.653286	8.026581	15.145024	H	4.303543	9.985257	12.821768
C	3.274933	8.266612	15.063779	H	5.967294	10.137436	12.183574
H	2.850258	8.735290	14.182079	C	7.561218	8.678090	17.644824
C	2.438362	7.896635	16.117509	C	6.971837	10.030808	17.201869
H	1.370505	8.081788	16.049145	H	7.300679	10.308532	16.194301
C	2.970005	7.277067	17.251524	H	7.311464	10.814609	17.888541
H	2.314883	6.971755	18.061996	H	5.877623	10.022190	17.213419
C	4.344976	7.062159	17.353795	C	7.090907	8.342209	19.067349
H	4.748295	6.593734	18.245439	H	6.000493	8.312305	19.141593
C	5.196418	7.458597	16.313606	H	7.444871	9.122308	19.752307
C	5.463145	7.015089	12.467962	H	7.492724	7.387996	19.421225
C	5.591619	5.632166	13.135039	C	9.101623	8.743568	17.602769
H	6.592161	5.472375	13.551770	H	9.561046	7.780872	17.847421
H	5.416929	4.851900	12.385469	H	9.451908	9.480285	18.334964
H	4.861313	5.498438	13.939076	H	9.473272	9.051157	16.619393
C	6.530941	7.161461	11.364873	C	7.408927	5.738323	17.102512
H	6.517610	8.156313	10.909138	H	7.134837	4.976416	16.368472
H	6.337915	6.425858	10.575418	H	6.859912	5.562021	18.030532
H	7.541914	6.981416	11.745928	H	8.482265	5.666519	17.283417
C	4.060390	7.177388	11.864919				

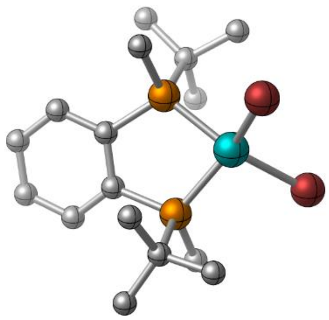

Zero-point correction= 0.409253 (Hartree/Particle)
Thermal correction to Energy= 0.436928
Thermal correction to Enthalpy= 0.437872
Thermal correction to Gibbs Free Energy= 0.350228
Sum of electronic and zero-point Energies= -7716.208672
Sum of electronic and thermal Energies= -7716.180997
Sum of electronic and thermal Enthalpies= -7716.180053
Sum of electronic and thermal Free Energies= -7716.267697

^{3}**2**

E(scf) = -5376.49528776 a.u.

ν_{min} = 25.2 cm^{-1}

C	-2.037907	1.377475	0.413023	C	0.017257	2.608328	-1.314622
C	-2.602008	0.138311	0.042033	C	-1.634206	-2.396392	1.233963
C	-3.995837	0.001750	-0.024420	C	-3.085057	-2.827549	1.483812
C	-4.826052	1.065692	0.328655	H	-3.109988	-3.541081	2.316959
C	-4.268740	2.270576	0.763356	H	-3.721745	-1.981526	1.755725
C	-2.882737	2.428573	0.799323	H	-3.522444	-3.325432	0.612249
H	-4.435710	-0.938197	-0.341426	C	-0.756134	-3.634232	0.965877
H	-5.904762	0.950435	0.277868	H	-0.766533	-4.283235	1.849742
H	-4.912858	3.092859	1.060959	H	-1.123279	-4.220920	0.118169
H	-2.467283	3.378448	1.118799	H	0.282149	-3.356873	0.757606
P	-1.418413	-1.238473	-0.250261	C	-1.089347	-1.638064	2.459855
P	-0.204706	1.536795	0.240807	H	-0.033559	-1.372739	2.320457
C	-2.054740	-2.136606	-1.713803	H	-1.650345	-0.717524	2.652158
H	-1.379759	-2.968209	-1.923729	H	-1.157936	-2.270471	3.352524
H	-3.075831	-2.504109	-1.582375	C	1.530222	2.718341	-1.578702
H	-2.020443	-1.453644	-2.567140	H	1.698742	3.337301	-2.468032
C	0.299123	2.584114	1.660525	H	2.062385	3.181489	-0.741762
H	1.372770	2.770897	1.597791	H	1.977775	1.737993	-1.759590
H	-0.235599	3.535882	1.694841	C	-0.586447	4.012486	-1.164701
H	0.102503	2.034449	2.584843	H	-0.407752	4.575674	-2.088834

H	-1.667495	3.981274	-1.005610	C	3.865335	0.518585	2.651210
H	-0.127742	4.575274	-0.346040	H	1.770491	0.088612	2.662129
C	-0.657636	1.875489	-2.490040	C	4.927407	0.581221	0.490816
H	-0.229854	0.879668	-2.648301	H	3.678556	0.129470	-1.192953
H	-1.735479	1.764316	-2.336467	C	5.013823	0.713423	1.880401
H	-0.506408	2.452688	-3.409773	H	3.913732	0.613108	3.734386
Fe	0.765211	-0.460482	-0.093497	H	5.815427	0.723816	-0.122306
C	2.525503	0.065117	0.628811	H	5.960577	0.961680	2.353583
C	2.650120	0.211295	2.028832	Br	1.589645	-1.973945	-1.779954
C	3.709959	0.254467	-0.113426				

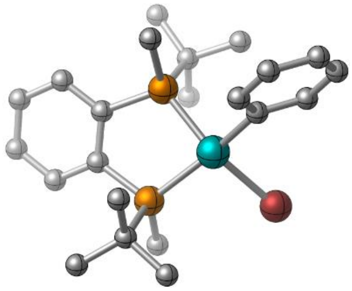

Zero-point correction= 0.497941 (Hartree/Particle)
Thermal correction to Energy= 0.529006
Thermal correction to Enthalpy= 0.529950
Thermal correction to Gibbs Free Energy= 0.436268
Sum of electronic and zero-point Energies= -5375.997347
Sum of electronic and thermal Energies= -5375.966282
Sum of electronic and thermal Enthalpies= -5375.965338
Sum of electronic and thermal Free Energies= -5376.059020

^{3}3

E(scf) = -3036.34130268 a.u.
ν_{min} = 29.8 cm^{-1}

C	-1.456196	-1.032574	0.166425	P	0.116312	-0.092010	0.437702
C	-1.852730	-2.071185	1.023986	P	-1.694128	0.606353	-2.130589
C	-2.289806	-0.680770	-0.924313	Fe	0.473789	0.838743	-1.738826
C	-3.521362	-1.336323	-1.072991	C	-0.229091	1.013241	1.947267
C	-3.065617	-2.734137	0.840939	C	-2.231726	0.090871	-3.896653
H	-3.351537	-3.536939	1.514410	C	1.111187	-0.862899	-2.536800
C	-3.910239	-2.353740	-0.202129	C	0.902339	-2.238580	-2.300089
H	-4.864702	-2.852233	-0.345153	C	2.134457	-0.548091	-3.460329
H	-1.212086	-2.367531	1.847390	C	1.661865	-3.230705	-2.926327
H	-4.186489	-1.060491	-1.882459	H	0.110121	-2.546671	-1.620140

C	2.901681	-1.529024	-4.101319	C	1.091382	1.735055	2.278089
H	2.340906	0.496968	-3.706845	H	1.474827	2.291362	1.417482
C	2.668342	-2.879193	-3.832620	H	0.919419	2.453171	3.089357
H	1.466816	-4.280222	-2.714882	H	1.866648	1.039749	2.616125
H	3.676101	-1.241029	-4.809336	C	-1.289573	2.047094	1.539563
H	3.257607	-3.648174	-4.325504	H	-2.232972	1.564540	1.264737
C	0.706715	2.810801	-1.534072	H	-1.491541	2.718910	2.382820
C	-0.010569	3.990735	-1.822738	H	-0.948147	2.654364	0.697128
C	2.051638	3.002456	-1.130594	C	-0.720125	0.240414	3.181202
C	0.558353	5.263748	-1.709123	H	-0.855564	0.942400	4.013826
H	-1.046557	3.923965	-2.142772	H	-1.683365	-0.245049	3.001522
C	2.640381	4.267226	-1.013166	H	-0.006249	-0.520205	3.511790
H	2.669097	2.135635	-0.874815	C	-1.982059	-1.405991	-4.138185
C	1.887710	5.407988	-1.300198	H	-2.185400	-1.638494	-5.191089
H	-0.034210	6.146940	-1.939867	H	-0.948570	-1.683711	-3.928530
H	3.675854	4.363537	-0.693271	H	-2.634442	-2.036787	-3.527896
H	2.330111	6.396596	-1.207256	C	-1.312617	0.914866	-4.823728
C	1.293050	-1.357044	1.088971	H	-1.578025	0.728430	-5.871717
H	2.255361	-0.867056	1.261810	H	-1.411125	1.991011	-4.639980
H	0.971973	-1.836632	2.016931	H	-0.262229	0.641059	-4.685170
H	1.439044	-2.120216	0.322262	C	-3.695590	0.423876	-4.244130
C	-2.870395	1.987360	-1.774704	H	-3.894893	0.098273	-5.272749
H	-2.814051	2.736552	-2.567422	H	-4.416364	-0.086868	-3.600339
H	-3.899278	1.624180	-1.704949	H	-3.902780	1.496146	-4.199894
H	-2.598606	2.460772	-0.832342				

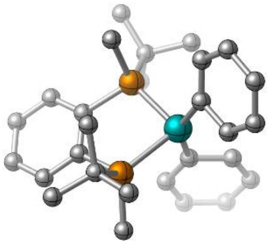

Zero-point correction= 0.585990 (Hartree/Particle)
Thermal correction to Energy= 0.620830
Thermal correction to Enthalpy= 0.621774
Thermal correction to Gibbs Free Energy= 0.520647
Sum of electronic and zero-point Energies= -3035.755313
Sum of electronic and thermal Energies= -3035.720473
Sum of electronic and thermal Enthalpies= -3035.719528
Sum of electronic and thermal Free Energies= -3035.820655

^{3}int-P

E(scf) = -3036.36887712 a.u.

ν_{min} = 22.5 cm^{-1}

C	-2.175967	-0.102727	0.029418	C	-0.417259	5.645496	0.875636
C	-3.175221	-1.083054	0.137720	H	0.555554	6.432011	-0.903270
C	-2.235171	0.817791	-1.041337	H	-1.024832	4.731438	2.714580
C	-3.347419	0.806792	-1.897908	H	-1.190616	6.407821	0.893052
C	-4.260708	-1.102285	-0.737650	H	-2.161574	3.876922	-1.213434
H	-5.027849	-1.864056	-0.631332	H	-2.056179	3.337292	-2.909761
C	-4.362208	-0.136335	-1.740586	H	-0.690776	4.184743	-2.139585
H	-5.215895	-0.131395	-2.412297	H	0.518374	-1.755448	0.390818
H	-3.111493	-1.841380	0.909451	H	0.606905	-1.693280	2.156717
H	-3.417557	1.525746	-2.707504	H	-0.824558	-2.396684	1.363276
P	-0.796886	0.086302	1.259390	C	0.744166	-0.194212	-2.314721
P	-0.766761	1.907879	-1.326968	H	-0.010252	-0.888778	-1.930197
Fe	0.579591	1.737407	0.590130	H	1.274509	-0.692228	-3.135429
C	-0.068719	-1.609138	1.301622	H	1.469587	-0.001125	-1.516609
C	-1.646691	0.282880	2.950225	C	-0.833213	0.801273	-4.000318
C	0.099282	1.109292	-2.820654	H	-0.239854	0.408275	-4.835677
C	-1.497288	3.467283	-1.979046	H	-1.582719	0.047838	-3.744700
C	2.527375	2.500904	0.681193	H	-1.352487	1.694856	-4.361809
C	3.483436	2.331160	-0.383725	C	1.194685	2.092187	-3.273517
C	2.419181	1.421848	1.667355	H	1.864744	2.355072	-2.451064
C	4.187945	1.164128	-0.541291	H	1.800218	1.626823	-4.061087
H	3.653795	3.149075	-1.077126	H	0.771001	3.014222	-3.684395
C	3.202038	0.238206	1.465252	C	-2.545147	1.527397	2.856131
H	2.155474	1.642059	2.700420	H	-1.979258	2.401300	2.518406
C	4.022135	0.078820	0.374200	H	-3.376107	1.371479	2.160191
H	4.895711	1.070172	-1.361099	H	-2.970101	1.755328	3.841355
H	3.143468	-0.546028	2.217465	C	-0.509361	0.538017	3.958225
H	4.587010	-0.838769	0.234548	H	0.092889	1.401348	3.668094
C	1.610227	3.622433	0.772174	H	-0.933535	0.735706	4.950130
C	1.551246	4.687782	-0.196535	H	0.157317	-0.326453	4.049322
C	0.658595	3.683179	1.866671	C	-2.472350	-0.922367	3.427117
C	0.572007	5.650279	-0.147105	H	-2.803347	-0.743756	4.458157
H	2.286912	4.721648	-0.994087	H	-3.370961	-1.072653	2.825131
C	-0.333304	4.696548	1.875084	H	-1.892773	-1.850986	3.427853
H	0.865945	3.192123	2.811782				

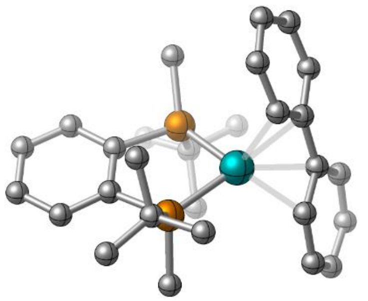

Zero-point correction= 0.586555 (Hartree/Particle)
Thermal correction to Energy= 0.620670
Thermal correction to Enthalpy= 0.621614
Thermal correction to Gibbs Free Energy= 0.522972
Sum of electronic and zero-point Energies= -3035.782322
Sum of electronic and thermal Energies= -3035.748208
Sum of electronic and thermal Enthalpies= -3035.747263
Sum of electronic and thermal Free Energies= -3035.845905

^{3}TS2'

E(scf) = -5915.21698730 a.u.

ν_{min} = -436.1 cm^{-1}

C	-1.855806	-0.766158	0.400463	C	4.249935	-0.971492	-1.673118
C	-2.601924	-1.806489	0.980099	H	2.278138	-0.803721	-2.497667
C	-2.523524	0.435422	0.070087	C	5.143044	-0.540545	-0.690542
C	-3.841854	0.636175	0.506886	H	5.410113	0.766827	1.006472
C	-3.927673	-1.613156	1.365924	H	4.556925	-1.721774	-2.398051
H	-4.478994	-2.429059	1.823830	H	6.149458	-0.947391	-0.645421
C	-4.532597	-0.369578	1.179871	Br	0.396675	2.806108	1.085375
H	-5.551495	-0.198166	1.514301	C	3.227156	4.207221	-0.817043
H	-2.152384	-2.777525	1.136938	H	2.813286	5.181008	-1.090154
H	-4.349611	1.568723	0.285721	H	2.622436	3.787738	-0.012821
P	-0.022861	-0.869184	0.120029	H	4.243038	4.373510	-0.430379
P	-1.753015	1.543524	-1.180301	C	3.655321	3.932256	-3.276331
Fe	0.708641	1.329042	-0.860274	O	3.534824	5.118902	-3.556028
C	0.784116	-1.456755	1.767550	O	4.173861	3.028584	-4.156603
C	-2.588941	0.967624	-2.797330	C	4.447188	3.541084	-5.465606
C	2.518482	0.524976	-0.808840	H	4.826934	2.697680	-6.045169
C	3.430202	0.939585	0.179996	H	3.536885	3.934059	-5.928524
C	2.955772	-0.440595	-1.728601	H	5.196796	4.337841	-5.431026
C	4.725881	0.420764	0.235254	C	3.266527	3.297604	-2.011808
H	3.132145	1.689846	0.904849	H	3.721750	2.328748	-1.863989

C	1.306193	2.600992	-2.371661
C	0.611243	3.864476	-2.430512
C	1.431928	1.919113	-3.631825
C	0.270009	4.460246	-3.630818
H	0.427097	4.392196	-1.499883
C	1.072840	2.517086	-4.835513
H	1.923703	0.953055	-3.656379
C	0.516857	3.803084	-4.852071
H	-0.197444	5.442268	-3.632572
H	1.233641	1.984965	-5.770368
H	0.253203	4.276715	-5.793172
C	0.164400	-2.349133	-0.962878
H	1.222306	-2.529183	-1.156987
H	-0.269333	-3.233215	-0.486779
H	-0.337488	-2.176016	-1.914803
C	2.160700	-2.072080	1.437196
H	2.063480	-3.036059	0.929520
H	2.786291	-1.420302	0.832522
H	2.686967	-2.254031	2.381899
C	0.959655	-0.240411	2.687170
H	1.647481	0.489964	2.263682
H	0.007523	0.262618	2.877926
H	1.365116	-0.578034	3.649132
C	-0.022891	-2.527170	2.521546
H	0.584520	-2.869891	3.368140
H	-0.957106	-2.136819	2.929711
H	-0.239160	-3.405844	1.905805
C	-2.492840	3.196781	-0.859044
H	-3.551052	3.232398	-1.129106
H	-2.371919	3.432187	0.198645
H	-1.947534	3.940382	-1.441760
C	-4.098069	0.713476	-2.657446
H	-4.505295	0.472582	-3.647289
H	-4.319063	-0.126539	-1.994881
H	-4.636894	1.592283	-2.289974
C	-2.355934	2.066660	-3.844212
H	-1.298671	2.292203	-3.960416
H	-2.733388	1.723600	-4.815380
H	-2.883097	2.99221	-3.594404
C	-1.89715	-0.329309	-3.242156
H	-2.063277	-1.132685	-2.518979
H	-2.311259	-0.65835	-4.202782
H	-0.818791	-0.190156	-3.375008

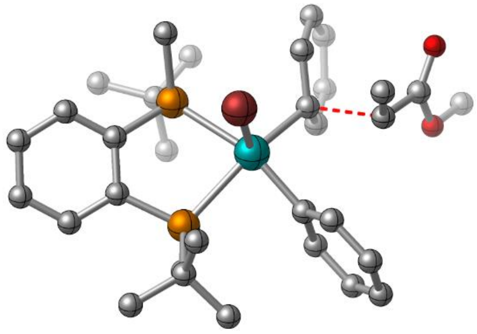

Zero-point correction= 0.695938 (Hartree/Particle)
Thermal correction to Energy= 0.739973
Thermal correction to Enthalpy= 0.740917
Thermal correction to Gibbs Free Energy= 0.620931
Sum of electronic and zero-point Energies= -5914.521049
Sum of electronic and thermal Energies= -5914.477014
Sum of electronic and thermal Enthalpies= -5914.476070
Sum of electronic and thermal Free Energies= -5914.596056

^{3}TS-a

E(scf) = -5915.22185151 a.u.

ν_{min} = -355.2 cm^{-1}

C	1.680413	-2.094268	0.206429
C	2.003206	-1.103985	1.159983
C	3.295799	-1.072984	1.707095
C	4.247059	-2.028079	1.352709
C	3.903269	-3.053565	0.470351
C	2.630261	-3.082866	-0.095886
H	3.562429	-0.308309	2.428163
H	5.244495	-1.983430	1.779964
H	4.627154	-3.821229	0.213315
H	2.384263	-3.877298	-0.790498
P	0.636016	-0.033863	1.786064
P	0.054703	-1.960305	-0.670953
C	1.454022	1.555548	2.218743
H	1.726836	2.054964	1.286787
H	0.742624	2.193812	2.746043
H	2.341827	1.428588	2.841536
C	-0.797774	-3.549169	-0.300315
H	-1.687269	-3.623768	-0.929695
H	-0.146515	-4.408393	-0.476445
H	-1.120825	-3.553500	0.740835
C	0.476878	-2.099882	-2.539330
C	0.210912	-0.863015	3.454088
C	1.401062	-0.905688	4.425075
H	1.074830	-1.360759	5.368511
H	2.225991	-1.509653	4.039592
H	1.780440	0.093849	4.659144
C	-0.945734	-0.085736	4.105580
H	-1.237640	-0.591060	5.034095
H	-0.657745	0.937061	4.368087
H	-1.826369	-0.044622	3.458271
C	-0.263200	-2.294298	3.153113
H	-1.137075	-2.289391	2.495570
H	0.518087	-2.899643	2.684815
H	-0.553713	-2.786900	4.088495
C	-0.684086	-1.439057	-3.303676
H	-0.489500	-1.503103	-4.381387
H	-1.635351	-1.937973	-3.104105
H	-0.785822	-0.383524	-3.041211
C	0.605271	-3.553843	-3.029161
H	0.852233	-3.538141	-4.097973
H	1.399262	-4.109611	-2.524749
H	-0.328293	-4.112438	-2.919220
C	1.777819	-1.336846	-2.832727
H	1.756427	-0.327823	-2.425737
H	2.657428	-1.839651	-2.425887
H	1.908680	-1.259126	-3.919139
Fe	-1.109276	0.015660	0.096071
C	-2.143949	1.674259	0.571972
C	-2.983235	2.294798	-0.382363
C	-2.036503	2.355827	1.800118
C	-3.655595	3.492881	-0.137846
H	-3.110494	1.834391	-1.357228
C	-2.717796	3.549439	2.072743
H	-1.403507	1.958638	2.581397
C	-3.531703	4.129793	1.101185
H	-4.281232	3.929991	-0.913602
H	-2.601181	4.026724	3.043893
H	-4.058202	5.059667	1.301286
C	2.034611	2.497431	-2.318110
C	3.172839	2.016009	-1.493353
O	3.829758	1.017061	-1.735597
O	3.397748	2.803944	-0.416367
C	1.792046	3.982882	-2.403486
H	1.699041	4.429093	-1.411529
H	0.881838	4.193649	-2.970743
H	2.631007	4.470360	-2.918651
H	2.006848	1.965674	-3.265731
Br	0.252067	1.695733	-1.290997
C	4.468936	2.378605	0.446613
H	5.431585	2.467100	-0.065254
H	4.438539	3.047001	1.307404
H	4.325484	1.342525	0.759553
C	-2.820576	-0.834692	-0.404135
C	-3.494886	-0.728881	-1.636222
C	-3.461473	-1.602612	0.589703
C	-4.717856	-1.363273	-1.873275
H	-3.056417	-0.140339	-2.437740
C	-4.686476	-2.241700	0.367363
H	-2.999421	-1.708958	1.570221
C	-5.321284	-2.129253	-0.871575

H	-5.200557	-1.259295	-2.842938	H	-6.271103	-2.625543	-1.052796
H	-5.144785	-2.825642	1.162971				

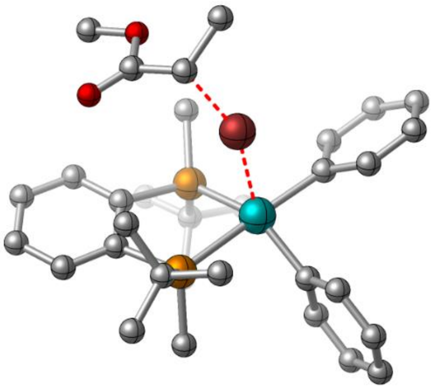

Zero-point correction= 0.696791 (Hartree/Particle)
Thermal correction to Energy= 0.741058
Thermal correction to Enthalpy= 0.742002
Thermal correction to Gibbs Free Energy= 0.618898
Sum of electronic and zero-point Energies= -5914.525060
Sum of electronic and thermal Energies= -5914.480793
Sum of electronic and thermal Enthalpies= -5914.479849
Sum of electronic and thermal Free Energies= -5914.602953

^{3}TSP

E(scf) = -3036.31676441 a.u.

ν_{min} = -360.6 cm^{-1}

C	-2.461004	-0.659435	-0.262694	C	-2.368293	3.213723	-1.654441
C	-3.311093	-1.774253	-0.202835	C	1.842589	1.802535	1.082951
C	-2.724639	0.355826	-1.210296	C	2.741783	1.190511	0.135017
C	-3.891314	0.286836	-1.986586	C	2.321307	1.849763	2.435205
C	-4.445995	-1.852581	-1.011573	C	3.893083	0.520900	0.543992
H	-5.095387	-2.721446	-0.950613	H	2.531602	1.263165	-0.931231
C	-4.752903	-0.806215	-1.883726	C	3.481529	1.203660	2.822686
H	-5.649868	-0.848954	-2.495192	H	1.743577	2.397002	3.175728
H	-3.094789	-2.588237	0.480910	C	4.274223	0.503319	1.889844
H	-4.127108	1.082045	-2.686546	H	4.518543	0.033375	-0.201675
P	-1.033086	-0.379074	0.899436	H	3.785508	1.237023	3.867222
P	-1.414252	1.652502	-1.424078	H	5.187429	0.006103	2.202773
Fe	0.126047	1.445974	0.233839	C	1.024566	3.233318	0.535057
C	-0.204019	-2.027872	0.938126	C	1.703375	4.100113	-0.361751
C	-1.866953	-0.208125	2.602993	C	0.151397	3.855911	1.473806
C	-0.673860	1.266698	-3.134003	C	1.447537	5.465211	-0.392760

H	2.436078	3.677659	-1.044562	H	-2.430797	0.491207	-4.190322
C	-0.114539	5.221948	1.431924	H	-2.201620	2.241991	-4.374150
H	-0.327923	3.249928	2.241214	C	0.414948	2.327320	-3.384071
C	0.523177	6.040650	0.493032	H	1.162380	2.327331	-2.585616
H	1.970803	6.093945	-1.110020	H	0.927826	2.116389	-4.330691
H	-0.813569	5.654601	2.144177	H	-0.002613	3.337230	-3.449726
H	0.329320	7.109029	0.470403	C	-2.807315	1.007373	2.520011
H	-2.882702	3.433239	-0.713969	H	-2.273414	1.905104	2.188637
H	-3.106567	3.177057	-2.460601	H	-3.633007	0.832070	1.822720
H	-1.662287	4.025928	-1.847228	H	-3.237839	1.215782	3.507157
H	0.265167	-2.190685	-0.037006	C	-0.729534	0.087671	3.598116
H	0.585504	-2.008430	1.694414	H	-0.161410	0.973492	3.302816
H	-0.879332	-2.862603	1.144920	H	-1.145199	0.263946	4.598060
C	-0.020499	-0.123485	-3.032901	H	-0.024073	-0.746814	3.672855
H	-0.760094	-0.903940	-2.826528	C	-2.654540	-1.437526	3.077667
H	0.479946	-0.372078	-3.976765	H	-3.024614	-1.259709	4.095755
H	0.730552	-0.153695	-2.235060	H	-3.524991	-1.636196	2.447685
C	-1.682634	1.281633	-4.290573	H	-2.035338	-2.339741	3.109814
H	-1.152927	1.113104	-5.237054				

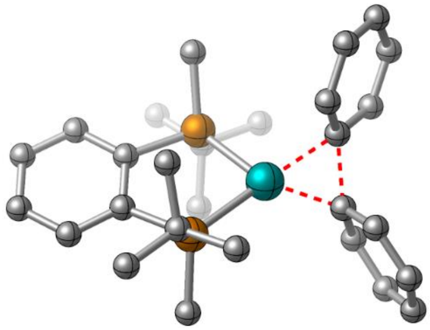

Zero-point correction= 0.583121 (Hartree/Particle)
Thermal correction to Energy= 0.617645
Thermal correction to Enthalpy= 0.618589
Thermal correction to Gibbs Free Energy= 0.517291
Sum of electronic and zero-point Energies= -3035.733644
Sum of electronic and thermal Energies= -3035.699120
Sum of electronic and thermal Enthalpies= -3035.698175
Sum of electronic and thermal Free Energies= -3035.799474

^{4}int2

E(scf) = -5144.81705631 a.u.

ν_{min} = 27.1 cm^{-1}

Br	2.059574	8.179714	13.268287	C	7.227575	10.186683	13.465513
Fe	4.353434	8.270190	13.759255	H	7.722404	11.130247	13.205481
P	5.392429	7.808625	15.787678	H	6.194433	10.421912	13.751476
P	6.345535	7.648121	12.804708	H	7.734085	9.769422	14.341750
C	7.077377	7.099982	15.455759	C	8.734625	8.979731	11.854968
C	7.968212	6.712592	16.468548	H	9.179670	9.928483	11.528787
H	7.722711	6.898957	17.508482	H	9.337680	8.600270	12.684392
C	9.174240	6.082982	16.158868	H	8.816821	8.276918	11.019649
H	9.848714	5.783083	16.955876	C	6.488525	9.813806	11.088021
C	9.504931	5.831386	14.826010	H	5.435964	9.975018	11.347645
H	10.433480	5.324383	14.579189	H	6.920209	10.782304	10.806869
C	8.650668	6.253463	13.806810	H	6.523754	9.167255	10.205251
H	8.937049	6.081845	12.774156	C	3.048113	7.019349	16.994471
C	7.446481	6.909899	14.103848	H	2.578090	7.356908	16.065525
C	4.454775	6.448613	16.728058	H	2.414150	6.242019	17.438460
C	7.276423	9.216841	12.269594	H	3.075147	7.862562	17.693316
C	5.722216	9.121319	17.038684	C	5.094904	6.009401	18.051828
H	4.769333	9.509757	17.407978	H	4.427176	5.298901	18.555613
H	6.319673	8.775375	17.887000	H	6.050164	5.501699	17.897378
H	6.255406	9.939905	16.546389	H	5.252744	6.849408	18.736118
C	6.512810	6.487478	11.382545	C	4.347575	5.245065	15.774006
H	7.535777	6.369348	11.014337	H	3.747469	4.453269	16.238530
H	6.135233	5.510332	11.698571	H	3.865436	5.528799	14.831843
H	5.879941	6.848048	10.567669	H	5.332785	4.829320	15.537395

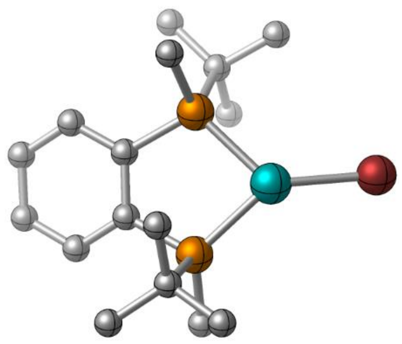

Zero-point correction= 0.406174 (Hartree/Particle)
Thermal correction to Energy= 0.432312
Thermal correction to Enthalpy= 0.433256
Thermal correction to Gibbs Free Energy= 0.349634
Sum of electronic and zero-point Energies= -5144.410882
Sum of electronic and thermal Energies= -5144.384745
Sum of electronic and thermal Enthalpies= -5144.383801
Sum of electronic and thermal Free Energies= -5144.467422

^{4}int7

E(scf) = -3343.48095079 a.u.

ν_{min} = 29.3 cm^{-1}

C	-1.728236	-0.918392	0.638478	H	1.332515	3.826230	-6.105313
C	-2.404281	-1.739069	1.555800	C	0.889550	2.167295	0.911146
C	-2.416066	0.193943	0.088794	C	-0.284243	2.127658	1.898148
C	-3.713992	0.486233	0.535191	H	-1.091503	2.787413	1.577189
C	-3.702154	-1.447480	1.972698	H	-0.713825	1.134744	2.016474
H	-4.195083	-2.094174	2.692558	H	0.018596	2.469405	2.899753
C	-4.352124	-0.317651	1.477508	C	1.196094	3.543013	0.527640
H	-5.354102	-0.067333	1.813258	O	0.392902	4.468546	0.397966
H	-1.913219	-2.610406	1.968531	O	2.531670	3.751990	0.281281
H	-4.239674	1.348253	0.140374	C	2.881773	5.039387	-0.239464
P	0.067822	-1.164143	0.223859	H	3.969201	5.034347	-0.335807
P	-1.616286	1.196655	-1.244237	H	2.428128	5.207068	-1.220942
Fe	0.774892	0.923648	-0.945035	H	2.569580	5.840045	0.437492
C	0.990051	-1.825525	1.774641	H	1.782181	1.695905	1.336402
C	-2.482249	0.604330	-2.843254	C	-2.232369	2.917764	-1.056117
C	2.628607	0.281523	-1.277052	H	-1.791236	3.378252	-0.172696
C	3.749387	0.953503	-0.759424	H	-1.879831	3.483094	-1.920766
C	2.869711	-0.924193	-1.961459	H	-3.322840	2.976495	-1.016930
C	5.038969	0.421790	-0.877064	C	0.076958	-2.650363	-0.872176
H	3.614130	1.904426	-0.255930	H	1.090875	-3.053056	-0.913815
C	4.154936	-1.457993	-2.092587	H	-0.602407	-3.420940	-0.499387
H	2.041401	-1.469694	-2.406270	H	-0.220249	-2.371623	-1.881821
C	5.250217	-0.790296	-1.538168	C	2.492358	-1.636732	1.479943
H	5.883256	0.963415	-0.455157	H	3.069279	-2.032625	2.324553
H	4.299111	-2.397728	-2.621681	H	2.811451	-2.167947	0.579414
H	6.251767	-1.202120	-1.630533	H	2.752848	-0.584730	1.353258
C	1.044980	1.831335	-2.727711	C	0.634552	-1.013868	3.030464
C	0.855114	3.226076	-2.779067	H	0.933142	0.030691	2.931777
C	1.352886	1.189940	-3.940438	H	-0.431466	-1.048811	3.270217
C	0.965221	3.939716	-3.977595	H	1.181113	-1.431307	3.884614
H	0.587279	3.771092	-1.880607	C	0.744305	-3.324602	2.043440
C	1.445998	1.891964	-5.146533	H	1.334638	-3.616772	2.920315
H	1.525735	0.118166	-3.955396	H	-0.297407	-3.564374	2.265970
C	1.255248	3.275509	-5.171629	H	1.068423	-3.955747	1.212771
H	0.808473	5.016336	-3.977269	C	-2.218590	1.620530	-3.971449
H	1.673740	1.356557	-6.065707	H	-2.606580	1.205236	-4.909507

H	-2.738381	2.566691	-3.796490	H	-4.443454	0.183925	-3.633770
H	-1.158721	1.827762	-4.112577	C	-1.882202	-0.758038	-3.214294
C	-4.003132	0.456373	-2.666657	H	-0.803031	-0.690091	-3.382194
H	-4.264699	-0.327518	-1.951852	H	-2.074988	-1.497481	-2.432403
H	-4.475799	1.389560	-2.344579	H	-2.341908	-1.126503	-4.139095

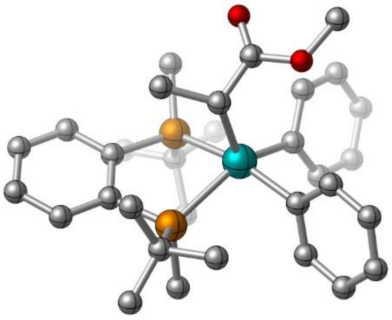

Zero-point correction= 0.698035 (Hartree/Particle)
Thermal correction to Energy= 0.739783
Thermal correction to Enthalpy= 0.740728
Thermal correction to Gibbs Free Energy= 0.626754
Sum of electronic and zero-point Energies= -3342.782915
Sum of electronic and thermal Energies= -3342.741167
Sum of electronic and thermal Enthalpies= -3342.740223
Sum of electronic and thermal Free Energies= -3342.854196

^{4}int8

E(scf) = -2804.67805269 a.u.

ν_{min} = 18.1 cm^{-1}

Fe	4.304418	8.211345	13.974191	C	2.501682	8.678865	13.169091
P	5.405238	7.818404	15.958065	C	1.474465	9.355364	13.879690
P	6.288123	7.478427	13.025318	C	2.111231	8.218858	11.883228
C	7.158106	7.264395	15.667850	C	0.188261	9.557862	13.367416
C	8.098612	7.058159	16.688650	H	1.684811	9.741904	14.877380
H	7.844761	7.286608	17.718721	C	0.829940	8.406842	11.352861
C	9.365101	6.547173	16.399969	H	2.841457	7.695065	11.265836
H	10.081514	6.390194	17.201566	C	-0.143317	9.080374	12.096118
C	9.702170	6.224720	15.083825	H	-0.557892	10.085901	13.958922
H	10.678832	5.806532	14.856498	H	0.588953	8.030298	10.360058
C	8.787136	6.456249	14.055578	H	-1.141182	9.232229	11.691589
H	9.068024	6.218464	13.034562	C	4.596056	6.327205	16.825735
C	7.524423	7.003493	14.329144	C	7.090434	8.965934	12.147495

C	5.599842	9.080114	17.294220	C	6.164742	9.326624	10.969574
H	4.607644	9.408302	17.615626	H	6.509160	10.255167	10.497459
H	6.156384	8.730126	18.168266	H	6.160383	8.546987	10.200582
H	6.121895	9.943943	16.871019	H	5.133323	9.477065	11.306191
C	6.425481	6.095032	11.808832	C	3.148845	6.745056	17.148731
H	5.761620	6.301394	10.965109	H	2.597028	5.890146	17.558863
H	7.438225	5.928183	11.430504	H	3.111346	7.550133	17.890355
H	6.078824	5.178805	12.296732	H	2.625670	7.088489	16.249484
C	7.096028	10.119049	13.167640	C	5.300850	5.867416	18.108805
H	6.082673	10.342314	13.524289	H	5.366282	6.666418	18.854352
H	7.713887	9.880488	14.039865	H	4.732895	5.044395	18.561573
H	7.496510	11.030355	12.706900	H	6.309881	5.497002	17.909682
C	8.514978	8.726439	11.630946	C	4.578536	5.177463	15.802209
H	8.861556	9.617934	11.092165	H	4.049209	5.468605	14.886787
H	9.219302	8.542195	12.446401	H	5.592112	4.872684	15.521164
H	8.566018	7.884278	10.933182	H	4.066478	4.304394	16.224852

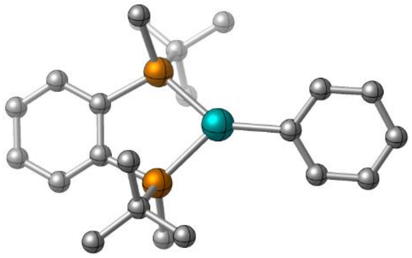

Zero-point correction= 0.493785 (Hartree/Particle)
Thermal correction to Energy= 0.523794
Thermal correction to Enthalpy= 0.524738
Thermal correction to Gibbs Free Energy= 0.431586
Sum of electronic and zero-point Energies= -2804.184268
Sum of electronic and thermal Energies= -2804.154259
Sum of electronic and thermal Enthalpies= -2804.153315
Sum of electronic and thermal Free Energies= -2804.246466

^{4}int-a

E(scf) = -5608.16513140 a.u.

ν_{min} = 32.6 cm^{-1}

C	-1.736083	-2.036463	-0.083544	C	-3.688736	-3.471169	0.132191
C	-2.309227	-3.314244	0.015636	H	-4.115813	-4.466932	0.205888
C	-2.576774	-0.901500	-0.126477	C	-4.513200	-2.345276	0.181881
C	-3.959263	-1.073545	0.048529	H	-5.586153	-2.457010	0.307047

H	-1.673948	-4.193625	0.024209
H	-4.614319	-0.210562	0.064359
P	0.086446	-1.784315	0.015299
P	-1.839578	0.737928	-0.564762
Fe	0.550567	0.383770	-0.960939
C	0.472287	-2.002045	1.863540
C	-2.890079	1.437497	-2.004162
C	0.986467	2.289223	-1.269795
C	1.489696	2.731291	-2.504735
C	0.741337	3.263743	-0.286868
C	1.701622	4.089058	-2.760041
H	1.699054	2.002377	-3.282334
C	0.955955	4.624449	-0.531147
H	0.375045	2.964309	0.693116
C	1.431156	5.044565	-1.775963
H	2.082845	4.402004	-3.729713
H	0.748568	5.354516	0.248268
H	1.596036	6.100329	-1.974055
C	2.458944	0.293381	-0.374498
C	2.987031	0.957370	0.745508
C	3.329319	-0.542850	-1.098645
C	4.309997	0.765501	1.155359
H	2.360467	1.638860	1.315072
C	4.657064	-0.730821	-0.701308
H	2.959433	-1.059049	-1.980137
C	5.152116	-0.085103	0.434525
H	4.684139	1.285304	2.034507
H	5.303504	-1.388355	-1.278505
H	6.181789	-0.234936	0.747948
Br	0.340282	-0.609632	-3.209656
C	-3.257308	0.304791	-2.979279
H	-2.368276	-0.237671	-3.309173
H	-3.961103	-0.407555	-2.541082
H	-3.732947	0.744249	-3.864533
C	-4.168302	2.149841	-1.524827
H	-4.849318	1.491913	-0.979221
H	-3.948309	3.016206	-0.894771
H	-4.710105	2.516449	-2.405161
C	-1.998054	2.463245	-2.730827
H	-1.646471	3.252508	-2.059860
H	-1.124945	1.982804	-3.174498
H	-2.578846	2.935624	-3.532494
C	-0.287925	-3.171958	2.508625
H	-1.367982	-3.006481	2.522212
H	-0.091977	-4.122073	2.001390
H	0.046155	-3.280094	3.547901
C	1.985688	-2.251067	2.011046
H	2.242078	-2.248609	3.077421
H	2.273715	-3.226350	1.606912
H	2.586133	-1.484310	1.521770
C	0.070088	-0.688698	2.555001
H	0.631518	0.163727	2.160313
H	-0.999442	-0.490720	2.431355
H	0.275285	-0.755003	3.630023
C	-2.244192	1.824447	0.865998
H	-2.035849	2.862371	0.597475
H	-3.293559	1.728774	1.155686
H	-1.614210	1.552697	1.715284
C	0.830799	-3.246787	-0.803978
H	1.901564	-3.063335	-0.917571
H	0.679869	-4.166286	-0.232861
H	0.391389	-3.348506	-1.797334

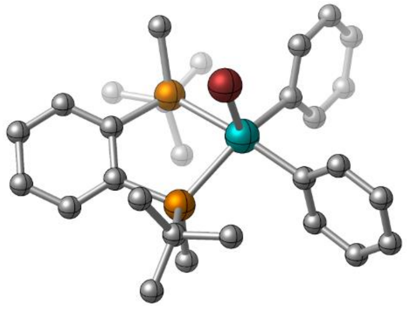

Zero-point correction= 0.588766 (Hartree/Particle)

Thermal correction to Energy= 0.625136
Thermal correction to Enthalpy= 0.626080
Thermal correction to Gibbs Free Energy= 0.522110
Sum of electronic and zero-point Energies= -5607.576365
Sum of electronic and thermal Energies= -5607.539996
Sum of electronic and thermal Enthalpies= -5607.539052
Sum of electronic and thermal Free Energies= -5607.643022

^{4}int-b

E(scf) = -5683.62635471 a.u.

ν_{min} = 34.6 cm^{-1}

C	2.949672	2.669089	-1.322241	C	3.757627	0.425982	2.205225
H	3.427843	2.810380	-0.351839	H	2.960549	-0.371617	0.375174
H	3.232970	1.685745	-1.706680	C	2.564810	2.297330	3.153338
H	3.350729	3.429141	-2.008083	H	0.854939	2.983692	2.059132
C	-2.026668	-1.252993	-0.153891	C	3.619372	1.383413	3.214429
C	-2.584280	-2.537702	-0.057714	H	4.581596	-0.283154	2.235995
C	-2.748162	-0.227888	-0.802286	H	2.456674	3.056685	3.924172
C	-4.069267	-0.483873	-1.204244	H	4.331315	1.422170	4.034494
C	-3.871482	-2.792328	-0.525647	Br	1.179459	-0.456128	-2.247918
H	-4.288985	-3.791662	-0.445814	C	-2.210092	1.560061	-3.132719
C	-4.631177	-1.751168	-1.063027	C	-1.065054	-0.591056	2.532949
H	-5.651170	-1.929328	-1.390585	C	0.530978	-2.333275	0.749260
H	-2.023131	-3.339010	0.410478	H	1.520984	-2.091272	1.140068
H	-4.661323	0.304723	-1.652870	H	0.081945	-3.112367	1.370373
P	-0.479203	-0.801623	0.730045	H	0.639638	-2.688024	-0.276515
P	-1.869894	1.340059	-1.253217	C	-2.877739	2.658697	-0.449571
Fe	0.492316	1.088952	-0.431690	H	-2.649547	3.614250	-0.919638
C	0.998326	4.017697	-0.549120	H	-3.947976	2.454431	-0.535777
O	1.643475	4.687595	0.243155	H	-2.608305	2.723659	0.606419
O	-0.280133	4.383220	-0.898885	C	-1.719242	0.795341	2.636224
C	-0.774599	5.568506	-0.260379	H	-1.017690	1.594510	2.381976
H	-1.749076	5.770392	-0.707796	H	-2.588311	0.869334	1.975034
H	-0.884590	5.418491	0.818325	H	-2.065420	0.967858	3.662092
H	-0.105322	6.416256	-0.430818	C	-2.086428	-1.660677	2.956751
C	1.433528	2.775144	-1.215239	H	-2.311248	-1.526603	4.021972
H	0.975003	2.728792	-2.202774	H	-3.026585	-1.578892	2.406598
C	1.756473	1.273221	1.091771	H	-1.700747	-2.677037	2.829683
C	2.836405	0.375488	1.155339	C	0.157207	-0.689221	3.466314
C	1.648945	2.244164	2.097457	H	-0.165639	-0.469324	4.491146

H	0.582029	-1.697600	3.466574	H	-3.681949	2.321998	-4.508413
H	0.944993	0.016545	3.207793	H	-4.417433	1.679332	-3.043926
C	-2.148551	0.195566	-3.843592	H	-3.609387	3.255369	-3.013575
H	-1.219873	-0.330995	-3.609947	C	-1.086645	2.455754	-3.688423
H	-2.991582	-0.448970	-3.584260	H	-0.126190	1.935524	-3.658244
H	-2.178027	0.364644	-4.927020	H	-1.304341	2.696033	-4.736165
C	-3.560521	2.241822	-3.421252	H	-0.997299	3.396290	-3.136799

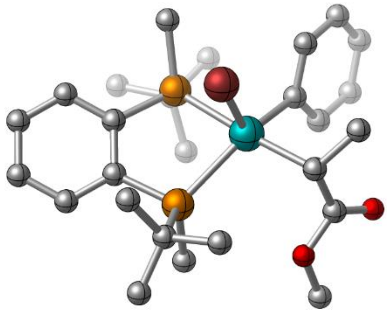

Zero-point correction= 0.608946 (Hartree/Particle)
Thermal correction to Energy= 0.647074
Thermal correction to Enthalpy= 0.648019
Thermal correction to Gibbs Free Energy= 0.541693
Sum of electronic and zero-point Energies= -5683.017409
Sum of electronic and thermal Energies= -5682.979280
Sum of electronic and thermal Enthalpies= -5682.978336
Sum of electronic and thermal Free Energies= -5683.084661

^{4}int-b-E
E(scf) = -5683.61738371 a.u.
ν_{min} = 30.2 cm^{-1}

C	-2.509154	-0.403487	0.385480	P	-1.869270	1.525423	-1.608633
C	-3.388291	-1.036468	1.278682	Fe	0.463353	1.052704	-0.723477
C	-2.997520	0.660262	-0.416012	C	-0.829773	-2.346865	-0.908716
C	-4.333312	1.068958	-0.263473	C	-0.099202	-1.730773	1.825884
C	-4.713623	-0.624706	1.407237	C	-2.460933	1.042423	-3.356907
H	-5.368647	-1.129017	2.111562	C	-2.392123	3.287259	-1.444135
C	-5.186873	0.438049	0.639138	C	1.586603	2.729211	-1.284684
H	-6.213905	0.776341	0.740047	C	0.625059	1.777035	1.135486
H	-3.041736	-1.857463	1.891889	C	1.842137	1.707009	1.832106
H	-4.716335	1.895812	-0.850218	C	-0.466890	2.377261	1.780969
P	-0.730156	-0.896948	0.227773	C	1.957119	2.181398	3.140099

H	2.717745	1.285537	1.344410	H	-1.141069	-0.692147	-3.464709
C	-0.357209	2.867111	3.087402	H	-2.515283	-0.740369	-4.578060
H	-1.426946	2.445769	1.279228	C	-1.608257	1.838608	-4.361751
C	0.855461	2.764711	3.773148	H	-1.739629	2.920030	-4.257579
H	2.908308	2.107083	3.661548	H	-1.914112	1.567535	-5.379489
H	-1.220932	3.318217	3.570152	H	-0.546179	1.604547	-4.260117
H	0.943192	3.140049	4.789158	C	-3.950595	1.338150	-3.592033
C	1.455910	3.937461	-0.423460	H	-4.204152	1.068998	-4.624798
O	2.298544	4.369158	0.345064	H	-4.196269	2.396320	-3.463938
O	0.262425	4.571884	-0.598071	H	-4.593723	0.748362	-2.933489
C	0.024407	5.690587	0.263957	H	-3.440641	3.444878	-1.705234
C	-0.626351	-3.166434	2.031824	H	-1.762093	3.906902	-2.082052
H	-1.711528	-3.221020	2.143207	H	-2.224601	3.606032	-0.414892
H	-0.186382	-3.563363	2.954736	H	-1.033441	-2.011249	-1.919826
H	-0.335161	-3.838824	1.221671	H	0.130660	-2.864476	-0.917513
C	1.430244	-1.812803	1.627178	H	-1.618498	-3.032441	-0.588854
H	1.884356	-2.260703	2.519178	H	-0.971119	6.058248	0.008588
H	1.873206	-0.827119	1.480375	H	0.765623	6.479471	0.104491
H	1.699742	-2.436994	0.769496	H	0.053461	5.382262	1.313111
C	-0.414957	-0.889554	3.074164	Br	1.628355	-0.373407	-2.297970
H	-1.490008	-0.772459	3.235084	C	1.334749	3.075622	-2.756017
H	0.031627	0.100968	3.024438	H	0.312524	3.418920	-2.918579
H	-0.001986	-1.397800	3.954053	H	2.007411	3.881653	-3.086928
C	-2.204367	-0.456176	-3.565492	H	1.516497	2.208178	-3.391391
H	-2.778792	-1.065063	-2.860468	H	2.612106	2.371171	-1.147980

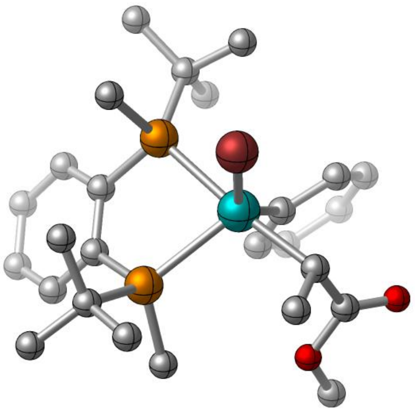

Zero-point correction= 0.608152 (Hartree/Particle)
Thermal correction to Energy= 0.646920
Thermal correction to Enthalpy= 0.647864
Thermal correction to Gibbs Free Energy= 0.539316
Sum of electronic and zero-point Energies= -5683.009231

Sum of electronic and thermal Energies= -5682.970464
Sum of electronic and thermal Enthalpies= -5682.969520
Sum of electronic and thermal Free Energies= -5683.078067

[4]TS2

E(scf) = -5608.15036713 a.u.

ν_{min} = -265.4 cm^{-1}

C	-2.133073	-0.986903	-0.387025
C	-2.789661	-2.225480	-0.477915
C	-2.680985	0.134925	-1.055602
C	-3.918121	-0.003869	-1.706448
C	-3.994321	-2.354044	-1.165835
H	-4.484550	-3.321435	-1.224363
C	-4.574500	-1.231859	-1.759522
H	-5.526753	-1.314448	-2.275315
H	-2.362765	-3.098340	0.004422
H	-4.372647	0.852540	-2.190074
P	-0.639971	-0.782078	0.682701
P	-1.699683	1.703969	-1.158119
Fe	0.464142	1.212408	-0.150155
C	0.276308	-2.365616	0.500211
C	-1.320562	-0.807412	2.449185
C	-1.723805	2.275382	-2.977715
C	-2.761819	2.925273	-0.274874
C	1.711955	1.816942	1.311421
C	2.933845	1.103634	1.203257
C	1.354601	2.281451	2.598184
C	3.706570	0.820345	2.326744
H	3.261539	0.750286	0.231315
C	2.138143	2.006096	3.716702
H	0.455894	2.875015	2.727398
C	3.317819	1.265515	3.595101
H	4.625657	0.251467	2.208211
H	1.822710	2.372602	4.690477
H	3.929579	1.053334	4.466838
C	1.286415	3.056108	-0.046766
C	2.342023	3.327676	-0.943750
C	0.474794	4.148280	0.341514
C	2.530240	4.602721	-1.475625
H	3.001773	2.522377	-1.245341
C	0.667561	5.421771	-0.184136
H	-0.335473	3.989134	1.046660
C	1.695725	5.660474	-1.104776
H	3.339933	4.770380	-2.181608
H	0.009690	6.232255	0.120308
H	1.848617	6.654502	-1.514601
H	-3.795474	2.899973	-0.629158
H	-2.355138	3.929064	-0.419902
H	-2.748672	2.699181	0.793816
Br	1.554019	-0.022963	-2.002744
C	-0.512586	3.222786	-3.111072
H	-0.472548	3.618204	-4.133557
H	-0.579085	4.074448	-2.427118
H	0.425190	2.700400	-2.910299
C	-2.994632	3.040573	-3.384762
H	-2.896430	3.353475	-4.431811
H	-3.901372	2.434365	-3.314999
H	-3.141381	3.945620	-2.787923
C	-1.517376	1.059590	-3.897924
H	-1.377294	1.411661	-4.927592
H	-0.626598	0.494981	-3.609677
H	-2.377754	0.384675	-3.889607
H	1.243927	-2.252001	0.996058
H	-0.246926	-3.227658	0.921884
H	0.461817	-2.525274	-0.563520
C	-0.101319	-0.945895	3.381206
H	-0.415819	-0.784621	4.419571
H	0.336698	-1.947226	3.321223
H	0.678304	-0.215876	3.153289
C	-2.017786	0.545459	2.670025
H	-1.325931	1.380480	2.527255
H	-2.860791	0.673270	1.982966
H	-2.407133	0.603677	3.693524

C	-2.310878	-1.946139	2.732635	H	-3.228370	-1.848329	2.146391
H	-2.589790	-1.922319	3.793688	H	-1.877588	-2.931164	2.531465

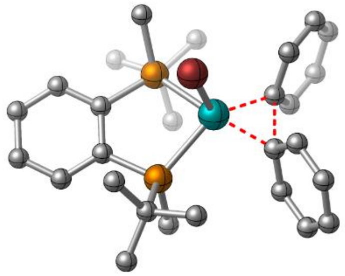

Zero-point correction= 0.587293 (Hartree/Particle)
Thermal correction to Energy= 0.623303
Thermal correction to Enthalpy= 0.624247
Thermal correction to Gibbs Free Energy= 0.520970
Sum of electronic and zero-point Energies= -5607.563074
Sum of electronic and thermal Energies= -5607.527064
Sum of electronic and thermal Enthalpies= -5607.526120
Sum of electronic and thermal Free Energies= -5607.629397

^{4}TS8

E(scf) = -3343.44536814 a.u.

ν_{min} = -336.5 cm^{-1}

C	-1.839800	-0.222768	0.501944	C	2.029915	-0.345226	-3.913377
C	-2.550713	-0.922691	1.492292	C	3.897711	-1.698873	-2.375817
C	-2.570678	0.516812	-0.458054	H	2.961930	-0.775950	-0.695568
C	-3.965147	0.631057	-0.322513	C	2.978649	-1.165890	-4.534638
C	-3.936239	-0.822291	1.599197	H	1.318120	0.170340	-4.556265
H	-4.458254	-1.366481	2.380946	C	3.923685	-1.849521	-3.764844
C	-4.644648	-0.015832	0.706875	H	4.625970	-2.223383	-1.759308
H	-5.722256	0.087327	0.795267	H	2.982909	-1.271675	-5.618269
H	-2.021519	-1.560552	2.188532	H	4.664994	-2.488875	-4.238278
H	-4.530911	1.215974	-1.039450	C	2.421421	3.512640	0.364254
P	0.018903	-0.197646	0.471875	H	2.318578	4.569747	0.113649
P	-1.694478	1.171562	-1.942301	H	1.564243	3.217141	0.970564
Fe	0.755750	1.232693	-1.524335	H	3.321507	3.404331	0.982695
C	0.597055	0.140476	2.256339	C	3.570850	3.188952	-1.818273
C	-2.199485	-0.020092	-3.334271	O	3.997942	4.333031	-1.836276
C	1.965241	-0.155574	-2.514749	O	3.995668	2.240616	-2.687197
C	2.936753	-0.874100	-1.781391	C	4.894335	2.687595	-3.710791

H	5.073956	1.816799	-4.342437
H	4.445375	3.494246	-4.297938
H	5.835994	3.043854	-3.281986
C	2.535733	2.671356	-0.891378
H	2.745669	1.623048	-0.668804
C	0.909604	3.178429	-2.046943
C	0.190701	4.249083	-1.459570
C	1.197067	3.287938	-3.436938
C	-0.247780	5.334747	-2.216716
H	-0.050845	4.217113	-0.401165
C	0.740968	4.366462	-4.189211
H	1.791549	2.514541	-3.912980
C	0.014873	5.405132	-3.588927
H	-0.812678	6.127915	-1.731320
H	0.963765	4.404790	-5.253479
H	-0.324841	6.253949	-4.175115
C	0.404708	-1.986247	0.217162
H	1.452605	-2.179077	0.455294
H	-0.230132	-2.625223	0.836876
H	0.246777	-2.234673	-0.833884
C	0.328342	-0.965601	3.292095
H	0.857817	-0.715398	4.220142
H	-0.728866	-1.055106	3.547588
H	0.693669	-1.943085	2.963542
C	2.126026	0.317953	2.144590
H	2.618036	-0.613559	1.844416
H	2.404594	1.092442	1.429975
H	2.533206	0.603716	3.121864
C	-0.066614	1.449066	2.717129
H	0.061821	2.256207	1.988988
H	-1.140969	1.314168	2.877532
H	0.376678	1.781122	3.663543
C	-2.584950	2.731312	-2.346898
H	-2.051195	3.234778	-3.154825
H	-3.621292	2.562282	-2.649385
H	-2.569590	3.387544	-1.474887
C	-1.669395	0.611282	-4.636380
H	-0.617919	0.898761	-4.552193
H	-1.757052	-0.110486	-5.457257
H	-2.240753	1.502311	-4.915578
C	-1.497349	-1.361375	-3.063200
H	-1.728239	-2.068179	-3.869570
H	-0.411543	-1.246674	-3.015716
H	-1.840044	-1.805896	-2.122651
C	-3.711822	-0.256538	-3.456976
H	-4.117872	-0.768299	-2.580439
H	-4.272216	0.671577	-3.605889
H	-3.902114	-0.894607	-4.329295

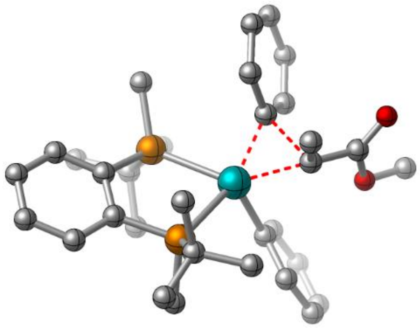

Zero-point correction= 0.693409 (Hartree/Particle)
Thermal correction to Energy= 0.735819
Thermal correction to Enthalpy= 0.736763
Thermal correction to Gibbs Free Energy= 0.619838
Sum of electronic and zero-point Energies= -3342.751959
Sum of electronic and thermal Energies= -3342.709549
Sum of electronic and thermal Enthalpies= -3342.708605
Sum of electronic and thermal Free Energies= -3342.825530

[4]TS8'

E(scf) = -3343.46727563 a.u.

ν_{min} = -274.0 cm^{-1}

C	-1.668182	-0.928779	0.541138	H	1.278091	3.378545	-6.227110
C	-2.317775	-1.787135	1.443627	C	0.999869	2.293891	0.860719
C	-2.371751	0.210072	0.063652	C	-0.122384	2.136970	1.898581
C	-3.655392	0.477849	0.565662	H	-0.963024	2.793294	1.665934
C	-3.601572	-1.516954	1.914558	H	-0.520850	1.124286	1.952095
H	-4.071828	-2.196363	2.619473	H	0.212417	2.405389	2.912810
C	-4.266246	-0.367492	1.489764	C	1.171305	3.691502	0.490376
H	-5.258136	-0.133677	1.865410	O	0.302459	4.559105	0.402863
H	-1.815288	-2.676593	1.799570	O	2.487392	4.018939	0.196726
H	-4.193587	1.355972	0.227055	C	2.707408	5.367057	-0.232030
P	0.117038	-1.171513	0.074921	H	3.773890	5.439946	-0.454768
P	-1.630042	1.282221	-1.254381	H	2.124639	5.607630	-1.127087
Fe	0.805388	0.997618	-0.928664	H	2.441502	6.080326	0.554374
C	1.060632	-1.922168	1.565904	H	1.948010	1.900924	1.245375
C	-2.587564	0.783311	-2.826062	C	-2.253883	2.985589	-0.936442
C	2.660819	0.513866	-1.613933	H	-1.765270	3.398768	-0.054844
C	3.710635	1.250617	-1.021491	H	-1.940624	3.602187	-1.782836
C	2.975412	-0.782162	-2.083833	H	-3.340334	3.049377	-0.838483
C	4.977249	0.691661	-0.844128	C	0.072818	-2.595758	-1.103618
H	3.524155	2.261232	-0.678305	H	1.083030	-2.996529	-1.214763
C	4.243317	-1.331528	-1.922249	H	-0.591199	-3.389578	-0.751727
H	2.209813	-1.377185	-2.570029	H	-0.268468	-2.256321	-2.081046
C	5.257380	-0.602497	-1.290396	C	2.553093	-1.754230	1.208852
H	5.753881	1.279632	-0.360276	H	3.164356	-2.121378	2.042321
H	4.439964	-2.338358	-2.283319	H	2.829818	-2.324536	0.319060
H	6.247512	-1.030225	-1.162304	H	2.816968	-0.708724	1.028988
C	1.501820	1.493314	-2.781986	C	0.775714	-1.125216	2.848467
C	1.736311	2.884945	-2.881982	H	1.092097	-0.084891	2.750851
C	1.231306	0.806022	-3.987890	H	-0.281092	-1.139806	3.128886
C	1.636423	3.555148	-4.100012	H	1.346039	-1.566068	3.675186
H	2.006005	3.439108	-1.992878	C	0.799060	-3.420707	1.812589
C	1.149764	1.473960	-5.207406	H	1.454931	-3.759904	2.624119
H	1.090425	-0.269357	-3.977232	H	-0.225553	-3.638375	2.121198
C	1.340888	2.858177	-5.276029	H	1.027422	-4.033346	0.936735
H	1.804976	4.629025	-4.131047	C	-2.226415	1.807823	-3.919930
H	0.928041	0.909404	-6.109890	H	-2.563300	1.431562	-4.893669

H	-2.723434	2.767743	-3.751777	H	-4.585329	0.553345	-3.608682
H	-1.151534	1.984973	-3.986657	C	-2.117986	-0.620023	-3.234881
C	-4.113344	0.764676	-2.640654	H	-1.046543	-0.638575	-3.446729
H	-4.434036	-0.011517	-1.940896	H	-2.335388	-1.355308	-2.45398
H	-4.503178	1.725964	-2.292265	H	-2.642979	-0.93632	-4.144444

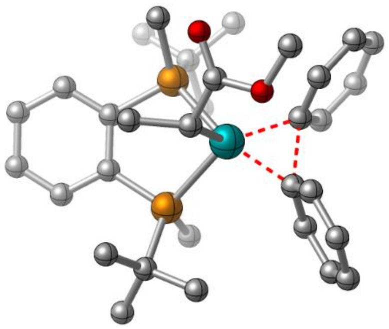

Zero-point correction= 0.694763 (Hartree/Particle)
Thermal correction to Energy= 0.736817
Thermal correction to Enthalpy= 0.737761
Thermal correction to Gibbs Free Energy= 0.621758
Sum of electronic and zero-point Energies= -3342.772513
Sum of electronic and thermal Energies= -3342.730459
Sum of electronic and thermal Enthalpies= -3342.729515
Sum of electronic and thermal Free Energies= -3342.845518

^{4}TS-b

E(scf) = -5683.60180613 a.u.

ν_{min} = -60.8 cm^{-1}

C	-2.709126	-0.735574	0.146846	C	-0.755731	-2.611167	-0.848995
C	-3.672936	-1.605268	0.682742	C	-0.434927	-1.729026	1.947020
C	-3.137127	0.476565	-0.450978	C	-2.232231	1.278635	-3.175691
C	-4.503182	0.805063	-0.422382	C	-2.515481	3.264707	-0.997141
C	-5.025941	-1.271973	0.687178	C	1.899995	3.350259	-1.575173
H	-5.750916	-1.959247	1.113382	H	1.174474	3.027488	-2.312926
C	-5.440568	-0.053309	0.148868	C	1.062503	1.897642	1.066415
H	-6.490770	0.223158	0.160559	C	2.370685	1.717543	1.559306
H	-3.368638	-2.557966	1.098300	C	0.204232	2.694398	1.850937
H	-4.846678	1.738080	-0.854983	C	2.796891	2.281811	2.765361
P	-0.899485	-1.114468	0.214198	H	3.077799	1.118366	0.986885
P	-1.913669	1.557226	-1.324341	C	0.613069	3.264789	3.062113
Fe	0.334505	0.912292	-0.561241	H	-0.816793	2.870910	1.516639

C	1.915917	3.059085	3.523340	H	-0.562903	-0.972730	3.972503
H	3.814281	2.118678	3.114526	C	-2.025175	-0.215931	-3.474158
H	-0.081094	3.866967	3.644803	H	-2.746780	-0.840205	-2.937701
H	2.242234	3.501153	4.461247	H	-1.013200	-0.535020	-3.211419
C	1.557728	4.476579	-0.730393	H	-2.161413	-0.393943	-4.547683
O	2.342070	5.092922	-0.018944	C	-1.166254	2.105063	-3.922074
O	0.239817	4.815056	-0.826858	H	-1.238815	3.173907	-3.695023
C	-0.189507	5.858026	0.058064	H	-1.306286	1.987011	-5.003404
C	3.316812	2.916712	-1.669389	H	-0.156667	1.760794	-3.678285
H	3.381220	1.871636	-1.979419	C	-3.637899	1.704948	-3.622902
H	3.830197	3.056747	-0.714719	H	-3.739971	1.521415	-4.699609
H	3.854745	3.519742	-2.419579	H	-3.824318	2.769977	-3.455413
C	-0.961474	-3.122989	2.326111	H	-4.419624	1.130325	-3.117917
H	-2.046461	-3.142552	2.452015	H	-3.546882	3.425309	-1.317981
H	-0.523668	-3.414228	3.289111	H	-1.855576	3.960691	-1.514618
H	-0.680715	-3.888610	1.597118	H	-2.444257	3.462590	0.074973
C	1.108919	-1.770163	1.924098	H	-0.938301	-2.330257	-1.886591
H	1.479275	-2.068221	2.912223	H	0.260031	-3.006261	-0.779538
H	1.532093	-0.792798	1.682734	H	-1.473505	-3.382753	-0.557132
H	1.487794	-2.494094	1.194583	H	-1.259929	5.977286	-0.115574
C	-0.915006	-0.689746	2.973063	H	0.330618	6.796279	-0.157334
H	-2.008156	-0.633123	3.006902	H	-0.005424	5.580048	1.099584
H	-0.523225	0.306972	2.753141	Br	1.644724	-0.236771	-2.242252

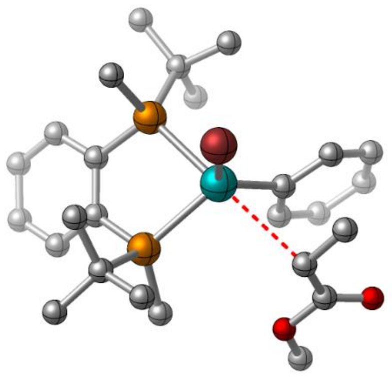

Zero-point correction= 0.604334 (Hartree/Particle)
Thermal correction to Energy= 0.643695
Thermal correction to Enthalpy= 0.644639
Thermal correction to Gibbs Free Energy= 0.533259
Sum of electronic and zero-point Energies= -5682.997472
Sum of electronic and thermal Energies= -5682.958111
Sum of electronic and thermal Enthalpies= -5682.957167
Sum of electronic and thermal Free Energies= -5683.068547

^{4}TS-b-E

E(scf) = -5683.59537576 a.u.

ν_{min} = -50.1 cm^{-1}

C	-2.689684	-0.695533	0.199126
C	-3.653915	-1.489581	0.841451
C	-3.098091	0.528149	-0.391324
C	-4.431281	0.946709	-0.246256
C	-4.977702	-1.069756	0.958485
H	-5.701764	-1.699967	1.466595
C	-5.363394	0.163524	0.431651
H	-6.388147	0.508383	0.534142
H	-3.372074	-2.448705	1.258341
H	-4.751146	1.890168	-0.675706
P	-0.892643	-1.144323	0.173447
P	-1.905160	1.505333	-1.414403
Fe	0.330695	0.867563	-0.634275
C	-0.805237	-2.646402	-0.890212
C	-0.377809	-1.791677	1.884119
C	-2.392322	1.096042	-3.206194
C	-2.414740	3.260266	-1.174438
C	2.055160	3.534599	-1.509617
C	0.927005	1.867276	1.035151
C	2.218363	1.741618	1.586276
C	-0.004300	2.599995	1.799274
C	2.558118	2.290723	2.825311
H	2.981222	1.191760	1.035567
C	0.318712	3.161151	3.039698
H	-1.021257	2.723545	1.430709
C	1.605983	3.004091	3.558775
H	3.564279	2.165971	3.220291
H	-0.431993	3.711640	3.603421
H	1.865353	3.434270	4.522955
C	1.630600	4.524620	-0.539136
O	2.299685	4.900572	0.414495
O	0.393001	5.034283	-0.812018
C	-0.115356	5.955731	0.162173
C	-0.872245	-3.206895	2.230109
H	-1.957279	-3.260448	2.345221
H	-0.434284	-3.506277	3.190540
H	-0.565355	-3.949495	1.488281
C	1.165590	-1.808786	1.826259
H	1.561382	-2.144564	2.792277
H	1.569150	-0.815427	1.619580
H	1.539764	-2.493844	1.057776
C	-0.846759	-0.796630	2.957803
H	-1.938583	-0.767001	3.032289
H	-0.487593	0.215635	2.757127
H	-0.452835	-1.103215	3.934447
C	-2.064166	-0.384942	-3.451407
H	-2.656791	-1.039846	-2.805180
H	-1.001941	-0.587684	-3.285132
H	-2.297245	-0.643135	-4.491422
C	-1.516742	1.971266	-4.119267
H	-1.692512	3.040106	-3.960070
H	-1.755615	1.750778	-5.166658
H	-0.454365	1.764595	-3.971027
C	-3.877109	1.350413	-3.505721
H	-4.069640	1.121160	-4.561046
H	-4.165835	2.393307	-3.343123
H	-4.531543	0.712354	-2.906057
H	-3.370535	3.502292	-1.645134
H	-1.631454	3.902127	-1.580805
H	-2.489219	3.462001	-0.103291
H	-0.942095	-2.367146	-1.934006
H	0.189985	-3.086283	-0.790696
H	-1.561885	-3.385530	-0.613391
H	-1.121670	6.216375	-0.169409
H	0.505766	6.855116	0.214567
H	-0.150527	5.489850	1.150767
Br	1.748999	-0.216549	-2.256335
C	1.525197	3.458554	-2.895930
H	0.466206	3.716748	-2.939447
H	2.058908	4.169307	-3.549518
H	1.675555	2.458409	-3.308526
H	2.969257	3.011231	-1.253999

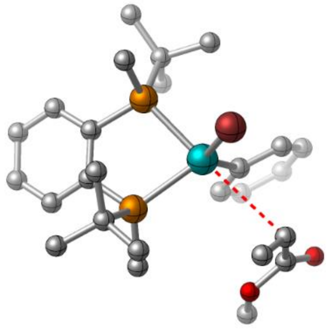

Zero-point correction= 0.603749 (Hartree/Particle)
Thermal correction to Energy= 0.643487
Thermal correction to Enthalpy= 0.644432
Thermal correction to Gibbs Free Energy= 0.531365
Sum of electronic and zero-point Energies= -5682.991626
Sum of electronic and thermal Energies= -5682.951888
Sum of electronic and thermal Enthalpies= -5682.950944
Sum of electronic and thermal Free Energies= -5683.064011

^{4}TS-c
E(scf) = -5683.60142581 a.u.
ν_{min} = -361.6 cm^{-1}

C	3.349060	2.242501	-1.474090
H	4.053325	2.563254	-0.703606
H	3.486164	1.178033	-1.671844
H	3.582317	2.786470	-2.397474
C	-2.228589	-1.220332	-0.106033
C	-2.980284	-2.397243	0.047392
C	-2.791702	-0.126808	-0.806800
C	-4.129045	-0.213251	-1.228569
C	-4.287938	-2.483103	-0.425849
H	-4.851597	-3.402563	-0.297457
C	-4.874818	-1.374935	-1.038939
H	-5.904503	-1.418423	-1.381687
H	-2.548777	-3.251607	0.557988
H	-4.596531	0.630713	-1.722068
P	-0.596386	-1.011423	0.728166
P	-1.724738	1.325774	-1.251134
Fe	0.546248	0.790600	-0.461136
C	0.200677	-2.666073	0.653623
H	1.211112	-2.568750	1.059821
H	-0.335354	-3.445066	1.201322
H	0.289690	-2.945824	-0.397958
C	-1.064966	-0.754770	2.546584
C	-1.973840	1.602777	-3.127937
C	-2.589273	2.741138	-0.446730
H	-2.153987	3.673193	-0.809502
H	-3.664318	2.736496	-0.644895
H	-2.428050	2.683167	0.632361
C	1.663454	3.946698	-0.764391
O	2.510219	4.774311	-0.481038
O	0.335344	4.259438	-0.816439
C	0.014064	5.618682	-0.473893
H	-1.070350	5.701130	-0.553186
H	0.334674	5.846800	0.546331
H	0.494461	6.318176	-1.163206
C	1.914813	2.516489	-1.067799
H	1.234856	2.227432	-1.871219
C	1.749074	1.739507	0.791102
C	2.901394	1.021602	1.200276

C	1.162912	2.623878	1.732498	C	-1.985401	0.240374	-3.844134
C	3.411337	1.159376	2.490294	H	-1.109300	-0.355901	-3.572052
H	3.385111	0.341734	0.506111	H	-2.885569	-0.337390	-3.618466
C	1.679280	2.752919	3.019853	H	-1.956330	0.407007	-4.928119
H	0.299174	3.213563	1.440175	C	0.258289	-0.706971	3.332132
C	2.810039	2.026377	3.410146	H	0.054865	-0.435464	4.375113
H	4.286367	0.582473	2.779888	H	0.760375	-1.680125	3.337001
H	1.198460	3.430092	3.721604	H	0.948439	0.032629	2.924105
H	3.218221	2.137358	4.410169	C	-1.968858	-1.851688	3.128042
C	-0.744432	2.404722	-3.605408	H	-2.135207	-1.648759	4.193574
H	-0.884217	2.693767	-4.654144	H	-2.947343	-1.878844	2.641448
H	-0.591865	3.318285	-3.021719	H	-1.517845	-2.846132	3.053098
H	0.164114	1.797892	-3.550129	C	-1.783174	0.602613	2.635255
C	-3.241926	2.400434	-3.476980	H	-1.146211	1.415480	2.277180
H	-3.294561	2.521578	-4.566049	H	-2.711710	0.605603	2.055676
H	-4.160713	1.898248	-3.165356	H	-2.039418	0.816413	3.679971
H	-3.232535	3.402425	-3.037586	Br	1.362602	-0.728798	-2.232982

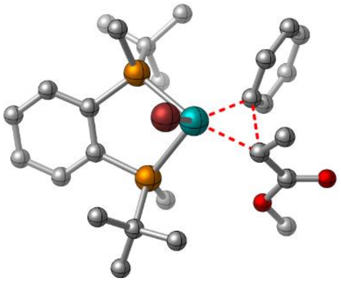

Zero-point correction= 0.605685 (Hartree/Particle)
Thermal correction to Energy= 0.644406
Thermal correction to Enthalpy= 0.645351
Thermal correction to Gibbs Free Energy= 0.535954
Sum of electronic and zero-point Energies= -5682.995741
Sum of electronic and thermal Energies= -5682.957019
Sum of electronic and thermal Enthalpies= -5682.956075
Sum of electronic and thermal Free Energies= -5683.065472

^{4}TS-c-E

E(scf) = -5683.59488112 a.u.

ν_{min} = -356.0 cm^{-1}

C	-2.300971	-1.176671	-0.029774	C	-2.817857	-0.106494	-0.798621
C	-3.107555	-2.301778	0.209209	C	-4.160891	-0.157442	-1.207689

C	-4.422261	-2.355978	-0.248598	C	3.245919	1.643149	3.104495
H	-5.028569	-3.235654	-0.053104	H	4.484089	0.061447	2.307299
C	-4.959875	-1.266008	-0.935157	H	1.840851	3.206116	3.596357
H	-5.992794	-1.283611	-1.270317	H	3.766205	1.689643	4.056456
H	-2.711933	-3.138520	0.775127	C	-0.700323	2.151574	-3.766420
H	-4.589392	0.671371	-1.758669	H	-0.817232	2.350843	-4.838592
P	-0.645083	-1.002917	0.762558	H	-0.526181	3.111480	-3.269233
P	-1.696504	1.266993	-1.349952	H	0.185931	1.524253	-3.639666
Fe	0.566063	0.651154	-0.560633	C	-3.195353	2.279628	-3.621181
C	0.063141	-2.699061	0.778550	H	-3.249592	2.344298	-4.715014
H	-0.491901	-3.406587	1.399599	H	-4.134541	1.838189	-3.279417
H	0.097850	-3.056044	-0.252409	H	-3.138007	3.302380	-3.236938
H	1.091826	-2.626743	1.141824	C	-2.053252	0.043541	-3.872414
C	-1.055634	-0.613085	2.570243	H	-1.202231	-0.578190	-3.580463
C	-1.963682	1.442227	-3.236146	H	-2.975817	-0.475222	-3.598835
C	-2.504523	2.759272	-0.622506	H	-2.037855	0.147305	-4.964521
H	-2.079712	3.665064	-1.062490	C	0.282377	-0.639939	3.332428
H	-3.585417	2.760685	-0.786407	H	0.129490	-0.269896	4.353536
H	-2.310572	2.773247	0.452964	H	0.681860	-1.656654	3.406860
C	3.403549	2.401366	-1.326136	H	1.036630	-0.009336	2.859696
O	4.070768	1.569339	-1.916706	C	-2.037829	-1.595532	3.224633
O	3.963599	3.445610	-0.668118	H	-2.160229	-1.327160	4.281682
C	5.399576	3.463208	-0.652210	H	-3.026191	-1.560651	2.758848
H	5.679364	4.356615	-0.092201	H	-1.676602	-2.628189	3.191060
H	5.789394	2.570351	-0.155105	C	-1.655974	0.803077	2.592714
H	5.804670	3.511863	-1.666951	H	-0.958245	1.539838	2.186245
C	1.917744	2.390497	-1.211410	H	-2.587341	0.852029	2.019377
C	1.889650	1.525025	0.611877	H	-1.882753	1.092975	3.625832
C	2.998969	0.678049	0.888695	Br	1.197039	-1.029416	-2.252825
C	1.505984	2.446901	1.615289	C	1.272930	3.762708	-1.099664
C	3.648988	0.731706	2.117724	H	0.195481	3.680500	-0.948830
H	3.349349	-0.003127	0.119601	H	1.690509	4.335623	-0.270869
C	2.169917	2.496274	2.841098	H	1.442952	4.336458	-2.018897
H	0.677332	3.124140	1.433949	H	1.563330	1.855367	-2.094052

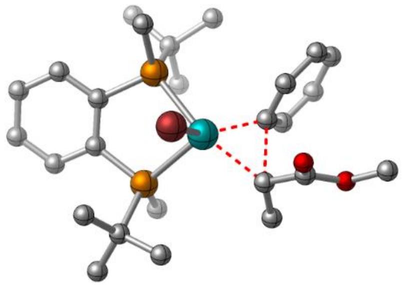

Zero-point correction= 0.605313 (Hartree/Particle)
Thermal correction to Energy= 0.644146
Thermal correction to Enthalpy= 0.645090
Thermal correction to Gibbs Free Energy= 0.534744
Sum of electronic and zero-point Energies= -5682.989568
Sum of electronic and thermal Energies= -5682.950735
Sum of electronic and thermal Enthalpies= -5682.949791
Sum of electronic and thermal Free Energies= -5683.060138

^{5}1

E(scf) = -7716.64464575 a.u.

ν_{min} = 23.0 cm^{-1}

Br	9.573791	6.627295	13.730814	H	3.279371	6.657742	12.807189
Br	8.718677	10.646265	14.307567	H	3.834591	5.916612	11.303180
Fe	8.087654	8.363300	14.331425	H	3.666119	7.671728	11.400814
P	5.815897	8.338529	13.516111	C	5.233335	9.828355	12.615563
P	7.067708	7.622206	16.405300	H	5.337674	10.690759	13.278967
C	4.700253	8.187218	14.978324	H	4.196364	9.756398	12.278713
C	3.318372	8.412120	14.869327	H	5.884924	9.983624	11.752100
H	2.898996	8.765033	13.933070	C	7.610019	8.616350	17.918899
C	2.466608	8.177554	15.947310	C	7.018958	10.031411	17.790709
H	1.400812	8.358696	15.843925	H	7.302032	10.498400	16.841987
C	2.985057	7.692243	17.149117	H	7.405598	10.660849	18.601139
H	2.324608	7.482069	17.985203	H	5.926708	10.022418	17.863756
C	4.357205	7.485026	17.280094	C	7.226781	7.997590	19.271472
H	4.743992	7.107586	18.219655	H	6.146134	7.988393	19.433030
C	5.234677	7.754543	16.217654	H	7.667678	8.600019	20.075231
C	5.428502	6.847774	12.421045	H	7.605433	6.976986	19.382587
C	5.784846	5.587031	13.230488	C	9.149002	8.682031	17.801769
H	6.838325	5.587246	13.526968	H	9.604757	7.686277	17.831642
H	5.613480	4.697473	12.612942	H	9.553389	9.260276	18.640753
H	5.166335	5.495168	14.129158	H	9.457521	9.169919	16.872299
C	6.361798	6.964664	11.199830	C	7.348898	5.855112	16.825238
H	6.137966	7.851741	10.598251	H	7.118529	5.246949	15.947436
H	6.229689	6.086053	10.557374	H	6.725103	5.529326	17.661881
H	7.413981	7.003946	11.500728	H	8.403381	5.708387	17.072051
C	3.964772	6.782209	11.964315				

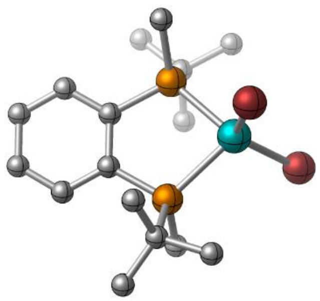

Zero-point correction= 0.408263 (Hartree/Particle)
Thermal correction to Energy= 0.436427
Thermal correction to Enthalpy= 0.437371
Thermal correction to Gibbs Free Energy= 0.348223
Sum of electronic and zero-point Energies= -7716.236383
Sum of electronic and thermal Energies= -7716.208219
Sum of electronic and thermal Enthalpies= -7716.207275
Sum of electronic and thermal Free Energies= -7716.296423

^{5}2

E(scf) = -5376.50814717 a.u.

ν_{min} = 17.5 cm^{-1}

C	-2.114930	-0.575587	-1.038370	C	-0.940907	-3.021582	0.172673
C	-2.419565	0.581391	-0.280640	C	-0.842825	3.122743	-0.044686
C	-3.716233	1.117307	-0.340534	C	-2.059642	4.052403	0.054014
C	-4.679435	0.570087	-1.186511	H	-1.779152	5.051193	-0.303321
C	-4.354656	-0.522270	-1.993322	H	-2.890008	3.707137	-0.567092
C	-3.086329	-1.094945	-1.909621	H	-2.414721	4.160651	1.083975
H	-3.976497	1.970999	0.275809	C	0.338716	3.729947	0.739786
H	-5.675508	1.001332	-1.223889	H	0.640335	4.677597	0.278577
H	-5.092055	-0.938664	-2.673396	H	0.069745	3.937077	1.781033
H	-2.858119	-1.960204	-2.522926	H	1.205150	3.059764	0.743789
P	-1.073579	1.395283	0.686310	C	-0.448384	2.923381	-1.519724
P	-0.500042	-1.428276	-0.749884	H	0.412400	2.253910	-1.624215
C	-1.785108	1.618988	2.364103	H	-1.275212	2.504720	-2.102758
H	-1.062202	2.151274	2.987288	H	-0.177701	3.888318	-1.964456
H	-2.734098	2.161906	2.354127	C	0.401934	-3.721795	0.463247
H	-1.942325	0.628109	2.798638	H	0.219703	-4.620459	1.064943
C	0.056256	-1.940825	-2.426159	H	0.904264	-4.038434	-0.457153
H	0.972600	-2.528136	-2.330582	H	1.079205	-3.072718	1.027035
H	-0.690000	-2.524600	-2.970780	C	-1.864917	-3.966227	-0.608424
H	0.291290	-1.038850	-2.997994	H	-2.017869	-4.882342	-0.024297

H	-2.848932	-3.523057	-0.783597	C	4.330972	1.621861	-2.018189
H	-1.438781	-4.260955	-1.572683	H	2.682852	2.529041	-1.000771
C	-1.612250	-2.606887	1.495737	C	4.496910	-0.779513	-1.863925
H	-0.958705	-1.962588	2.091631	H	2.977260	-1.759738	-0.717237
H	-2.558792	-2.084552	1.321657	C	4.996527	0.436500	-2.339586
H	-1.829486	-3.502974	2.089633	H	4.713783	2.573800	-2.380724
Fe	0.989228	0.104602	0.454486	H	5.009241	-1.708634	-2.105568
C	2.634344	0.379121	-0.733373	H	5.894599	0.459384	-2.951919
C	3.173787	1.584350	-1.230900	Br	1.484825	-0.594333	2.698821
C	3.339607	-0.797185	-1.079687				

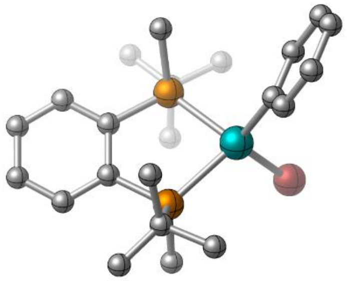

Zero-point correction= 0.496775 (Hartree/Particle)
Thermal correction to Energy= 0.528595
Thermal correction to Enthalpy= 0.529539
Thermal correction to Gibbs Free Energy= 0.432156
Sum of electronic and zero-point Energies= -5376.011372
Sum of electronic and thermal Energies= -5375.979552
Sum of electronic and thermal Enthalpies= -5375.978608
Sum of electronic and thermal Free Energies= -5376.075991

^{5}**3**
E(scf) = -3036.36987747 a.u.
ν_{min} = 17.3 cm^{-1}

C	-1.947852	-1.028129	0.280565	P	-0.215059	-0.407254	0.453857
C	-2.500010	-1.942085	1.193574	P	-1.996033	0.560490	-2.056737
C	-2.765786	-0.502161	-0.748664	Fe	0.446072	0.776711	-1.608026
C	-4.129946	-0.836817	-0.770482	C	-0.297810	0.796227	1.921062
C	-3.845593	-2.299497	1.128368	C	-2.368537	-0.253245	-3.732089
H	-4.253224	-3.007490	1.844144	C	1.692530	-0.472827	-2.686477
C	-4.668892	-1.726242	0.157421	C	1.751867	-1.878862	-2.563779
H	-5.725359	-1.975315	0.117946	C	2.565095	0.086095	-3.647629
H	-1.879147	-2.370324	1.973474	C	2.606216	-2.674508	-3.335719
H	-4.782544	-0.397833	-1.516509	H	1.101202	-2.378118	-1.846487

C	3.426905	-0.689935	-4.431716	C	1.142692	1.296573	2.143109
H	2.573285	1.166775	-3.788959	H	1.555877	1.746514	1.234566
C	3.449213	-2.078824	-4.277954	H	1.147018	2.061880	2.928683
H	2.614932	-3.755184	-3.206053	H	1.809793	0.489584	2.464351
H	4.079584	-0.215253	-5.162008	C	-1.194090	1.970246	1.488721
H	4.115173	-2.688986	-4.883360	H	-2.222356	1.644695	1.300135
C	1.075344	2.747378	-1.488284	H	-1.221862	2.724884	2.283955
C	0.260659	3.900365	-1.510284	H	-0.808282	2.451217	0.585378
C	2.466367	2.992433	-1.410537	C	-0.843017	0.177571	3.215299
C	0.781329	5.199017	-1.457479	H	-0.825970	0.934217	4.009781
H	-0.821157	3.786988	-1.567594	H	-1.877878	-0.157783	3.103663
C	3.008042	4.281198	-1.350361	H	-0.239653	-0.667965	3.560135
H	3.153623	2.146167	-1.399911	C	-1.890072	-1.713154	-3.659630
C	2.162368	5.393971	-1.373821	H	-1.990602	-2.180972	-4.646518
H	0.113143	6.057972	-1.479278	H	-0.837176	-1.777748	-3.368947
H	4.085702	4.420170	-1.287529	H	-2.483068	-2.298545	-2.949362
H	2.574459	6.399189	-1.329484	C	-1.505190	0.523459	-4.748126
C	0.732040	-1.859315	1.079569	H	-1.643441	0.097226	-5.749095
H	1.790976	-1.590786	1.110631	H	-1.784959	1.581845	-4.796196
H	0.418230	-2.192464	2.072170	H	-0.441694	0.462048	-4.496994
H	0.612154	-2.687951	0.377241	C	-3.839290	-0.204417	-4.172652
C	-3.041937	2.077976	-2.020335	H	-3.922250	-0.618742	-5.185296
H	-2.764325	2.728492	-2.853741	H	-4.485224	-0.804793	-3.527424
H	-4.109744	1.851248	-2.080966	H	-4.230630	0.816982	-4.206129
H	-2.850145	2.610534	-1.085447				

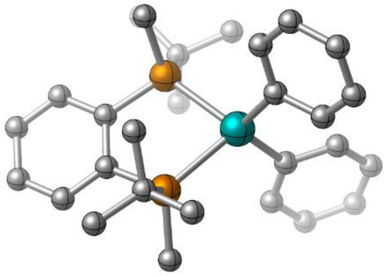

Zero-point correction= 0.585369 (Hartree/Particle)
Thermal correction to Energy= 0.620564
Thermal correction to Enthalpy= 0.621508
Thermal correction to Gibbs Free Energy= 0.518272
Sum of electronic and zero-point Energies= -3035.784509
Sum of electronic and thermal Energies= -3035.749313
Sum of electronic and thermal Enthalpies= -3035.748369
Sum of electronic and thermal Free Energies= -3035.851605

[5]int-P

E(scf) = -3036.35245248 a.u.

ν_{min} = 19.7 cm^{-1}

C	-1.996276	-0.605189	-0.123854
C	-2.916970	-1.659498	-0.022168
C	-2.224739	0.421856	-1.068320
C	-3.422950	0.425172	-1.799214
C	-4.079945	-1.671144	-0.794586
H	-4.783307	-2.493866	-0.701659
C	-4.347979	-0.611581	-1.662117
H	-5.267745	-0.596404	-2.239982
H	-2.739732	-2.470075	0.676955
H	-3.638250	1.236796	-2.485663
P	-0.545076	-0.422342	1.014508
P	-0.873064	1.661028	-1.330181
Fe	0.838975	1.258316	0.208467
C	0.036797	-2.157582	1.261193
C	-1.350893	0.046336	2.679351
C	-0.309300	1.395645	-3.129281
C	-1.765301	3.274536	-1.339848
C	2.277936	2.548450	1.144846
C	2.913162	1.741085	0.076018
C	2.564927	2.120813	2.498971
C	3.760378	0.651316	0.435258
H	3.063853	2.168070	-0.912662
C	3.363940	1.041607	2.788380
H	2.172435	2.711244	3.321595
C	3.972069	0.270983	1.745826
H	4.267398	0.120730	-0.368900
H	3.555523	0.786448	3.827699
H	4.611513	-0.572934	1.986048
C	1.595166	3.811882	0.867553
C	1.742274	4.494011	-0.379089
C	0.736551	4.438950	1.821939
C	1.058817	5.673842	-0.653086
H	2.412309	4.093463	-1.131628
C	0.057231	5.613988	1.535784
H	0.578261	3.968892	2.787748
C	0.197664	6.250495	0.289670
H	1.206075	6.155744	-1.617545
H	-0.598529	6.041979	2.290869
H	-0.339092	7.167972	0.068659
H	-2.160126	3.448055	-0.334868
H	-2.591565	3.311176	-2.055427
H	-1.055968	4.074638	-1.561255
H	0.318891	-2.563061	0.284856
H	0.931365	-2.132332	1.889081
H	-0.701618	-2.821400	1.719929
C	0.306267	-0.013345	-3.192404
H	-0.440013	-0.788986	-2.989732
H	0.722842	-0.196884	-4.190316
H	1.116025	-0.126997	-2.461005
C	-1.409589	1.529089	-4.191428
H	-0.959445	1.455030	-5.189702
H	-2.154191	0.733411	-4.111649
H	-1.924170	2.493908	-4.136868
C	0.786023	2.446331	-3.391346
H	1.584109	2.381929	-2.646358
H	1.229420	2.277692	-4.380262
H	0.389731	3.466652	-3.373805
C	-1.846566	1.496008	2.531802
H	-1.027523	2.180538	2.286155
H	-2.601853	1.581834	1.742930
H	-2.299454	1.836112	3.471261
C	-0.240710	-0.021872	3.743611
H	0.634731	0.569378	3.460071
H	-0.619961	0.371848	4.694958
H	0.088531	-1.050892	3.922206
C	-2.519660	-0.853959	3.104640
H	-2.863727	-0.550181	4.101738
H	-3.370344	-0.771323	2.423546
H	-2.230624	-1.907990	3.168562

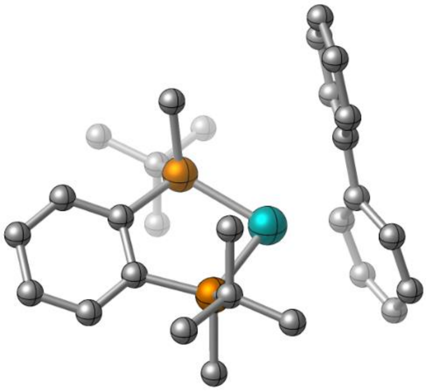

Zero-point correction= 0.584595 (Hartree/Particle)
Thermal correction to Energy= 0.619201
Thermal correction to Enthalpy= 0.620145
Thermal correction to Gibbs Free Energy= 0.519149
Sum of electronic and zero-point Energies= -3035.767857
Sum of electronic and thermal Energies= -3035.733252
Sum of electronic and thermal Enthalpies= -3035.732308
Sum of electronic and thermal Free Energies= -3035.833303

^{5}TS2'

E(scf) = -5915.22778248 a.u.

ν_{min} = -426.3 cm^{-1}

C	-1.688043	-0.711143	0.597724	C	4.436017	-0.748315	-1.661607
C	-2.294038	-1.690221	1.402235	H	3.691998	0.684120	-0.255731
C	-2.498944	0.289125	0.015707	C	2.942114	-1.360594	-3.453072
C	-3.865222	0.338272	0.339839	H	1.030767	-0.396082	-3.455957
C	-3.656711	-1.648967	1.689274	C	4.178217	-1.493720	-2.815629
H	-4.099734	-2.414235	2.319831	H	5.399947	-0.830211	-1.164689
C	-4.440615	-0.613691	1.178660	H	2.730259	-1.926021	-4.357731
H	-5.498626	-0.556386	1.416985	H	4.933320	-2.164978	-3.216219
H	-1.698187	-2.490986	1.822038	Br	0.496994	2.781581	1.037755
H	-4.495167	1.117939	-0.072832	C	2.758875	4.998246	-0.692814
P	0.148820	-0.664201	0.355599	H	2.459346	5.726990	-1.449439
P	-1.772106	1.380047	-1.279858	H	1.972058	4.888813	0.053367
Fe	0.728934	1.388837	-0.999967	H	3.649826	5.400235	-0.185826
C	0.927757	-0.924281	2.094897	C	3.981460	3.712240	-2.472464
C	-2.477235	0.693189	-2.904492	O	4.210526	4.697838	-3.162341
C	2.206049	0.250179	-1.759479	O	4.524691	2.495155	-2.732251
C	3.460595	0.110979	-1.147840	C	5.295362	2.398442	-3.934955
C	1.975813	-0.496779	-2.927084	H	5.585050	1.349883	-4.015194

H	4.700026	2.692093	-4.804986	H	0.020749	0.892731	2.903765
H	6.185576	3.033744	-3.887619	H	-0.876063	-0.584438	3.309584
C	3.090504	3.678259	-1.312852	H	0.655652	-0.229699	4.118050
H	3.202308	2.826877	-0.654889	C	1.041368	-2.408071	2.493512
C	1.190783	2.945115	-2.359029	H	1.515365	-2.464185	3.481298
C	0.324830	4.063696	-2.218451	H	0.074377	-2.910049	2.573840
C	1.596331	2.642091	-3.687455	H	1.666916	-2.977664	1.800882
C	-0.104033	4.811623	-3.311266	C	-2.611468	2.998299	-1.040947
H	-0.002023	4.349338	-1.225649	H	-3.698695	2.911796	-0.995110
C	1.179216	3.395821	-4.780468	H	-2.239680	3.431548	-0.109641
H	2.279627	1.819221	-3.852834	H	-2.341548	3.661734	-1.863743
C	0.317921	4.484850	-4.605504	C	-4.007769	0.783926	-2.999893
H	-0.769930	5.656835	-3.152250	H	-4.326897	0.397639	-3.975996
H	1.525287	3.130948	-5.776828	H	-4.504329	0.183683	-2.233082
H	-0.009445	5.073362	-5.457861	H	-4.367363	1.814489	-2.923882
C	0.469208	-2.253896	-0.519076	C	-1.838969	1.512390	-4.040276
H	1.546017	-2.423644	-0.584407	H	-0.748800	1.448600	-4.024556
H	-0.007004	-3.094583	-0.008721	H	-2.185779	1.126969	-5.006755
H	0.076236	-2.185783	-1.534024	H	-2.111152	2.570467	-3.989182
C	2.352125	-0.341860	2.023945	C	-2.04173	-0.776526	-3.021666
H	2.969437	-0.877990	1.298753	H	-2.472153	-1.396774	-2.229652
H	2.335918	0.717793	1.758963	H	-2.375724	-1.181505	-3.984316
H	2.829775	-0.441386	3.006475	H	-0.953991	-0.876769	-2.983054
C	0.121172	-0.162840	3.162271				

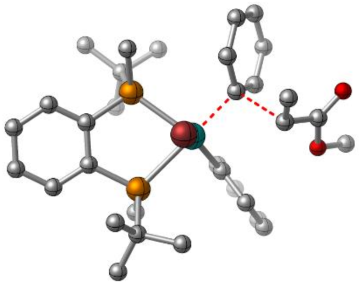

Zero-point correction= 0.695943 (Hartree/Particle)
Thermal correction to Energy= 0.740098
Thermal correction to Enthalpy= 0.741042
Thermal correction to Gibbs Free Energy= 0.620130
Sum of electronic and zero-point Energies= -5914.531840
Sum of electronic and thermal Energies= -5914.487685
Sum of electronic and thermal Enthalpies= -5914.486740
Sum of electronic and thermal Free Energies= -5914.607652

^{5}TS-a

E(scf) = -5915.21362643 a.u.
ν_{min} = -309.0 cm^{-1}

C	0.768967	-2.580976	0.045668
C	1.129915	-1.891064	1.228025
C	2.233498	-2.341251	1.970747
C	2.945433	-3.476503	1.589533
C	2.549359	-4.194664	0.460355
C	1.472389	-3.746264	-0.301783
H	2.540147	-1.801405	2.859995
H	3.798344	-3.803379	2.177125
H	3.083470	-5.093353	0.165747
H	1.186817	-4.306797	-1.184007
P	0.123679	-0.451479	1.804468
P	-0.550517	-1.879092	-1.044696
C	1.374209	0.664243	2.579961
H	1.628483	0.359537	3.598174
H	2.279825	0.664012	1.972628
H	0.977442	1.681350	2.600386
C	-1.937093	-3.081116	-0.857902
H	-2.695217	-2.882411	-1.618131
H	-1.587625	-4.112608	-0.950276
H	-2.399841	-2.943629	0.119454
C	-0.025612	-2.096107	-2.871328
C	-0.870719	-1.111873	3.279993
C	-0.037297	-1.885356	4.312724
H	-0.682605	-2.172157	5.152678
H	0.383813	-2.801944	3.890919
H	0.782777	-1.287993	4.723137
C	-1.503275	0.122938	3.950752
H	-2.210933	-0.201023	4.723294
H	-0.749733	0.751834	4.435750
H	-2.052669	0.743174	3.236494
C	-1.965421	-2.034071	2.720163
H	-2.603331	-1.514158	2.000027
H	-1.528509	-2.910026	2.229475
H	-2.602476	-2.394363	3.537116
C	-0.886908	-1.079063	-3.649364
H	-0.607110	-1.095918	-4.709445
H	-1.952604	-1.321433	-3.588641
H	-0.747687	-0.057946	-3.278971
C	-0.292047	-3.500362	-3.442754
H	0.001257	-3.510784	-4.499843

H	0.286146	-4.279839	-2.940050
H	-1.348866	-3.776172	-3.395729
C	1.457783	-1.735289	-3.051221
H	1.661547	-0.734203	-2.673603
H	2.121205	-2.438342	-2.541115
H	1.705170	-1.755229	-4.119580
Fe	-1.152880	0.375311	-0.227281
C	-1.204394	2.402603	-0.233946
C	-1.518790	3.113252	-1.408359
C	-1.180650	3.129861	0.969112
C	-1.801039	4.481194	-1.384646
H	-1.532082	2.590849	-2.363589
C	-1.467932	4.498453	1.005463
H	-0.941076	2.621498	1.900175
C	-1.777362	5.180946	-0.173681
H	-2.037218	5.003681	-2.309225
H	-1.445970	5.032979	1.952951
H	-1.994895	6.245624	-0.151069
C	3.610271	0.681915	-1.248502
C	4.243071	1.237256	-0.039368
O	4.419010	0.613694	1.000924
O	4.586889	2.538373	-0.181513
C	3.907652	1.284913	-2.592560
H	3.264122	0.850971	-3.362074
H	4.950670	1.085545	-2.877445
H	3.763948	2.367073	-2.584786
H	3.551925	-0.401438	-1.200380
Br	1.391148	1.198975	-0.864019
C	5.113158	3.168903	0.997921
H	6.026823	2.671132	1.334947
H	5.328717	4.199129	0.712412
H	4.377206	3.148260	1.807250
C	-3.191084	0.164440	-0.213092
C	-3.902571	-0.281510	-1.344585
C	-3.971947	0.567176	0.888922
C	-5.300254	-0.345177	-1.373769
H	-3.357354	-0.590279	-2.232633
C	-5.368868	0.499822	0.881837
H	-3.482477	0.95073	1.780709
C	-6.040761	0.039125	-0.253561

H	-5.810271	-0.697135	-2.268179	H	-7.126403	-0.013515	-0.266904
H	-5.932913	0.811575	1.758398				

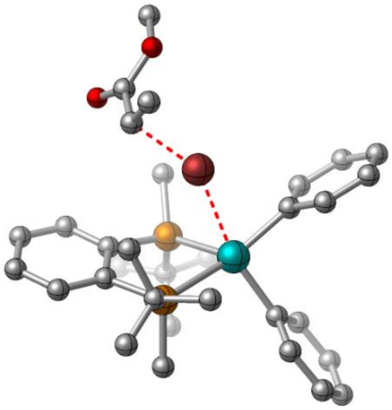

Zero-point correction= 0.694694 (Hartree/Particle)
Thermal correction to Energy= 0.739828
Thermal correction to Enthalpy= 0.740772
Thermal correction to Gibbs Free Energy= 0.613387
Sum of electronic and zero-point Energies= -5914.518933
Sum of electronic and thermal Energies= -5914.473799
Sum of electronic and thermal Enthalpies= -5914.472855
Sum of electronic and thermal Free Energies= -5914.600240

^{5}TSP

E(scf) = -3036.30128516 a.u.

ν_{min} = -459.6 cm^{-1}

C	-2.609961	-0.373537	-0.191111	Fe	0.660834	0.774604	-0.355356
C	-3.748198	-1.125349	0.140377	C	-0.768240	-2.494388	0.514849
C	-2.730724	0.680251	-1.126318	C	-1.213539	-0.283607	2.443600
C	-4.002564	1.024009	-1.611162	C	-0.996639	0.684232	-3.494990
C	-5.002392	-0.791785	-0.372643	C	-1.625183	3.211855	-2.063705
H	-5.873481	-1.379181	-0.096785	C	2.572887	1.115200	-0.869255
C	-5.134067	0.303833	-1.226877	C	3.036877	2.027263	-1.890825
H	-6.110582	0.584355	-1.611276	C	3.585489	0.193909	-0.394095
H	-3.663913	-1.973327	0.810865	C	4.344148	2.033885	-2.349777
H	-4.114552	1.847145	-2.308976	H	2.335226	2.748539	-2.302986
P	-0.954348	-0.660308	0.595089	C	4.891756	0.214838	-0.859980
P	-1.170718	1.450877	-1.755888	H	3.314274	-0.547313	0.355977

C	5.303062	1.134001	-1.843365	H	-0.586567	-1.302085	-4.270540
H	4.629771	2.741958	-3.126943	H	0.111842	-1.011090	-2.666016
H	5.604671	-0.513067	-0.473865	C	-2.213680	0.905329	-4.404420
H	6.325204	1.140944	-2.210187	H	-2.010210	0.467722	-5.390015
C	1.843687	2.173169	0.591022	H	-3.112938	0.423461	-4.012260
C	1.197740	3.409012	0.361183	H	-2.428917	1.967435	-4.559179
C	2.345295	1.931497	1.885360	C	0.251416	1.313040	-4.140684
C	0.987131	4.318779	1.402504	H	1.147482	1.142025	-3.538540
H	0.867709	3.667408	-0.640863	H	0.418616	0.861961	-5.126561
C	2.148537	2.843579	2.921361	H	0.138279	2.392270	-4.289585
H	2.905410	1.019075	2.076316	C	-1.408870	1.239094	2.560497
C	1.452912	4.036991	2.689250	H	-0.539738	1.790352	2.190794
H	0.468016	5.254110	1.204662	H	-2.289227	1.572065	1.999552
H	2.541471	2.628973	3.912719	H	-1.555414	1.515363	3.611696
H	1.296077	4.746186	3.497112	C	0.086764	-0.702096	3.155814
H	-2.493497	3.340049	-2.715262	H	0.209726	-1.789838	3.172987
H	-0.770347	3.720191	-2.517308	H	0.969044	-0.261227	2.683278
H	-1.832389	3.685986	-1.100178	H	0.061972	-0.355223	4.195951
H	-0.736181	-2.788044	-0.538215	C	-2.400623	-1.004870	3.097687
H	0.182124	-2.775474	0.975708	H	-2.390262	-0.804239	4.176567
H	-1.579712	-3.037025	1.008388	H	-3.358910	-0.651563	2.709486
C	-0.763350	-0.824618	-3.299228	H	-2.351425	-2.090888	2.968472
H	-1.627991	-1.311423	-2.836623				

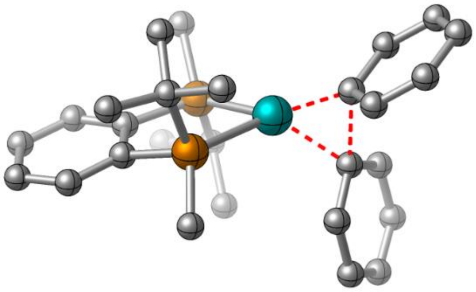

Zero-point correction= 0.583154 (Hartree/Particle)
Thermal correction to Energy= 0.617616
Thermal correction to Enthalpy= 0.618560
Thermal correction to Gibbs Free Energy= 0.517745
Sum of electronic and zero-point Energies= -3035.718131
Sum of electronic and thermal Energies= -3035.683669
Sum of electronic and thermal Enthalpies= -3035.682725
Sum of electronic and thermal Free Energies= -3035.783540

^{6}int2

E(scf) = -5144.76468422 a.u.

ν_{min} = 30.2 cm^{-1}

Br	2.566210	8.092260	12.954700	H	5.984289	5.322407	17.642578
Fe	4.523093	8.822388	14.075586	H	4.366251	4.677886	17.962434
P	5.365049	7.877791	16.144906	H	4.856443	6.214223	18.684497
P	6.477387	7.895630	13.024614	C	5.397776	8.869580	17.692198
C	7.059225	7.400818	15.761422	H	5.954428	9.791132	17.499864
C	7.983691	7.075887	16.766914	H	5.869742	8.341809	18.524830
H	7.713613	7.244891	17.807519	H	4.375396	9.132475	17.975102
C	9.225911	6.510327	16.486934	C	7.379989	9.269060	12.059994
H	9.918053	6.274072	17.289128	C	7.757397	10.339949	13.095525
C	9.548553	6.195267	15.124886	H	6.868772	10.717545	13.620970
H	10.481083	5.684865	14.894967	H	8.237846	11.194020	12.602833
C	8.687982	6.533616	14.107556	H	8.447063	9.945934	13.848935
H	8.963192	6.292344	13.082743	C	8.636696	8.772881	11.333180
C	7.441426	7.205622	14.347717	H	9.373737	8.372755	12.035898
C	4.372427	6.312589	16.545330	H	9.110217	9.602790	10.792258
C	4.467766	5.387634	15.317929	H	8.403296	7.994438	10.599208
H	4.042855	5.860435	14.426375	C	6.371537	9.856090	11.056800
H	3.903189	4.466969	15.508739	H	6.112661	9.145171	10.266010
H	5.504670	5.114440	15.101869	H	6.789665	10.748975	10.575605
C	2.908309	6.723869	16.778500	H	5.438678	10.153368	11.555425
H	2.798889	7.380589	17.648076	C	6.196714	6.568155	11.771121
H	2.299587	5.830026	16.961805	H	5.554937	5.808226	12.224238
H	2.492085	7.233953	15.902665	H	7.134868	6.098308	11.461538
C	4.934906	5.598729	17.782378	H	5.685691	6.968586	10.891729

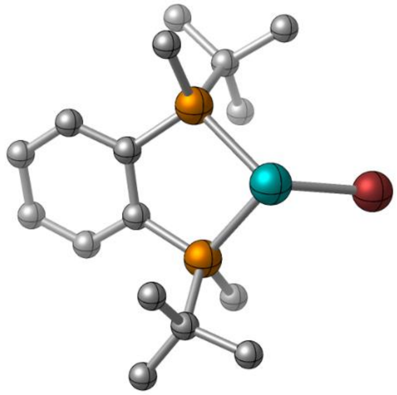

Zero-point correction= 0.402210 (Hartree/Particle)
Thermal correction to Energy= 0.428952
Thermal correction to Enthalpy= 0.429896
Thermal correction to Gibbs Free Energy= 0.344832
Sum of electronic and zero-point Energies= -5144.362474
Sum of electronic and thermal Energies= -5144.335732

Sum of electronic and thermal Enthalpies= -5144.334788
Sum of electronic and thermal Free Energies= -5144.419852

^{6}int7
E(scf) = -3343.46644975 a.u.
ν_{min} = 20.6 cm^{-1}

C	-1.970918	-1.229932	0.633482	H	2.098635	2.292640	-5.964463
C	-2.816367	-1.992589	1.458376	H	1.201739	4.611330	-6.078452
C	-2.440852	0.019246	0.152763	C	1.355002	2.684150	0.781934
C	-3.699938	0.480638	0.579894	C	0.088152	2.784342	1.628658
C	-4.065816	-1.527200	1.862061	H	-0.578494	3.546342	1.219033
H	-4.692546	-2.139453	2.504069	H	-0.461895	1.839944	1.670421
C	-4.500002	-0.273627	1.435311	H	0.310779	3.073177	2.666345
H	-5.466796	0.111100	1.746646	C	1.881037	3.992802	0.376209
H	-2.496839	-2.973465	1.789857	O	1.238236	5.031896	0.258684
H	-4.083003	1.432880	0.234882	O	3.219003	3.954337	0.087827
P	-0.283076	-1.839294	0.150521	C	3.758065	5.152347	-0.488198
P	-1.511752	1.012000	-1.115826	H	4.814767	4.946840	-0.667252
Fe	1.011472	1.484791	-0.976179	H	3.258970	5.388934	-1.432907
C	0.673966	-2.061326	1.792765	H	3.651604	6.002377	0.192496
C	-2.061059	0.311867	-2.815390	H	2.137776	2.094116	1.267469
C	2.639651	0.208127	-1.108510	C	-2.337856	2.660172	-1.044318
C	3.848105	0.537601	-0.465403	H	-2.312647	3.031332	-0.018691
C	2.641222	-0.958187	-1.900045	H	-1.772172	3.346907	-1.675527
C	4.987745	-0.268271	-0.576417	H	-3.372517	2.633273	-1.394284
H	3.911518	1.447748	0.126244	C	-0.671202	-3.584434	-0.357617
C	3.774962	-1.766479	-2.027250	H	0.257953	-4.083435	-0.647566
H	1.733082	-1.260578	-2.415793	H	-1.164084	-4.179027	0.416705
C	4.952881	-1.427202	-1.354919	H	-1.323336	-3.551347	-1.235721
H	5.902450	0.009574	-0.057029	C	0.854539	-0.661161	2.400717
H	3.738760	-2.663869	-2.641221	H	-0.104746	-0.218669	2.687294
H	5.834724	-2.056869	-1.441489	H	1.475130	-0.721742	3.303552
C	1.137970	2.637087	-2.684525	H	1.354833	0.014751	1.702331
C	0.661034	3.959965	-2.775644	C	0.032072	-2.987550	2.836036
C	1.667872	2.067160	-3.859836	H	0.723109	-3.111622	3.680038
C	0.679993	4.666733	-3.983132	H	-0.895305	-2.572544	3.238584
H	0.281644	4.456528	-1.886944	H	-0.176096	-3.986229	2.439036
C	1.689828	2.763204	-5.072979	C	2.055969	-2.622631	1.397417
H	2.069827	1.056695	-3.829740	H	2.566069	-1.980488	0.674723
C	1.188375	4.065933	-5.138261	H	2.688990	-2.690273	2.291196
H	0.300425	5.685408	-4.023225	H	1.984104	-3.629493	0.972027

C	-3.456420	-0.324728	-2.726059	H	-2.884921	2.183095	-3.635245
H	-3.768842	-0.643125	-3.728090	H	-1.138024	2.000652	-3.885628
H	-3.465104	-1.203654	-2.075339	C	-1.03459	-0.733336	-3.282058
H	-4.206244	0.380409	-2.352725	H	-0.044951	-0.286138	-3.413637
C	-2.087898	1.463757	-3.840425	H	-0.94374	-1.569134	-2.584597
H	-2.277838	1.039111	-4.833391	H	-1.346911	-1.138912	-4.25205

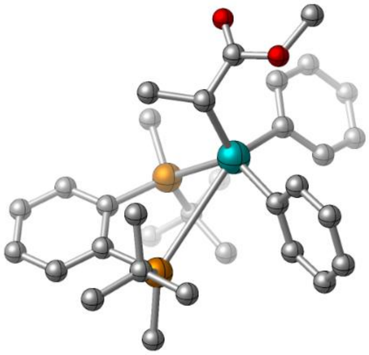

Zero-point correction= 0.694774 (Hartree/Particle)
Thermal correction to Energy= 0.737849
Thermal correction to Enthalpy= 0.738793
Thermal correction to Gibbs Free Energy= 0.617992
Sum of electronic and zero-point Energies= -3342.771675
Sum of electronic and thermal Energies= -3342.728601
Sum of electronic and thermal Enthalpies= -3342.727656
Sum of electronic and thermal Free Energies= -3342.848458

^{6}int8

E(scf) = -2804.64094125 a.u.

ν_{min} = 17.6 cm^{-1}

Fe	4.348860	8.392202	13.992784	C	1.395758	9.235164	14.083523
P	5.476189	7.834658	16.055066	C	2.066178	8.097763	12.086040
P	6.429860	7.545967	12.889309	C	0.102816	9.389233	13.572048
C	7.122709	7.266714	15.628350	H	1.603948	9.627783	15.079079
C	8.093134	6.989098	16.619131	C	0.778398	8.241752	11.558628
H	7.872693	7.247673	17.653400	H	2.816454	7.586697	11.481747
C	9.309933	6.391784	16.335257	C	-0.209148	8.891441	12.303840
H	10.031739	6.210070	17.126394	H	-0.661261	9.894843	14.158658
C	9.602133	6.008583	14.976313	H	0.543424	7.849939	10.571289
H	10.531926	5.495578	14.743268	H	-1.212239	9.007257	11.901262
C	8.701359	6.292300	13.974949	C	7.226497	9.049871	12.026879
H	8.952937	6.011858	12.953549	C	4.492081	6.414542	16.850761
C	7.450819	6.940464	14.217866	C	6.380114	6.242677	11.582129
C	2.423566	8.588247	13.362280	H	5.783599	6.582560	10.731279

H	7.379339	5.974037	11.227175	H	6.665643	10.445666	10.456313
H	5.908817	5.350884	12.004083	H	6.279602	8.766583	10.063926
C	5.670181	9.086769	17.394553	H	5.278905	9.658428	11.225819
H	4.686861	9.419164	17.739067	C	3.066872	6.921550	17.131269
H	6.233177	8.708090	18.252049	H	2.440583	6.099712	17.500623
H	6.203377	9.947694	16.981391	H	3.053809	7.711095	17.890232
C	7.286122	10.152764	13.097992	H	2.595916	7.315039	16.222696
H	6.281477	10.417897	13.457087	C	5.139107	5.899943	18.142915
H	7.881913	9.842357	13.962574	H	4.548626	5.067221	18.546564
H	7.735404	11.063997	12.684172	H	6.153676	5.532063	17.964058
C	8.637199	8.750641	11.504784	H	5.184272	6.674004	18.916224
H	8.632304	7.960982	10.745618	C	4.446301	5.285945	15.805939
H	9.065739	9.649093	11.041238	H	3.970145	5.619968	14.875822
H	9.305366	8.442314	12.314579	H	5.450354	4.927875	15.558318
C	6.307810	9.497651	10.878164	H	3.865200	4.439228	16.191223

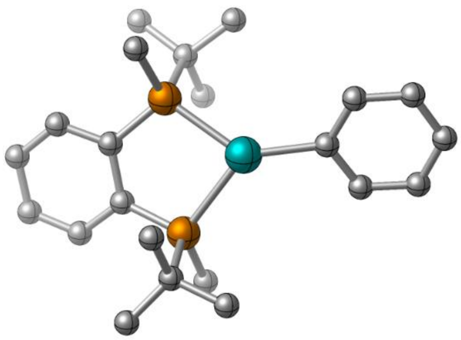

Zero-point correction= 0.490297 (Hartree/Particle)
Thermal correction to Energy= 0.520818
Thermal correction to Enthalpy= 0.521762
Thermal correction to Gibbs Free Energy= 0.427547
Sum of electronic and zero-point Energies= -2804.150644
Sum of electronic and thermal Energies= -2804.120123
Sum of electronic and thermal Enthalpies= -2804.119179
Sum of electronic and thermal Free Energies= -2804.213394

^{6}int-a

E(scf) = -5608.13432234 a.u.

ν_{min} =24.2 cm^{-1}

C	2.779619	-0.575785	-0.412836	C	4.167132	-0.573124	-0.637170
C	2.158499	-1.778046	-0.001390	H	2.498845	-3.828256	0.602807
C	2.956244	-2.904552	0.267017	H	4.933671	-3.748199	0.303849
C	4.336579	-2.867051	0.088065	H	6.013555	-1.675307	-0.566946
C	4.941848	-1.704372	-0.392901	H	4.652213	0.324850	-1.000997

P	0.317689	-1.903052	0.044412	H	4.665788	2.244504	-0.369840
P	1.760805	0.961975	-0.518342	H	3.575624	3.455877	-1.070059
C	-0.010854	-3.124471	1.379537	C	3.112002	1.632203	1.866548
H	0.338830	-4.128318	1.123739	H	2.280435	1.140707	2.377499
H	0.488082	-2.786884	2.291202	H	3.909606	0.899905	1.711334
H	-1.085822	-3.158815	1.562361	H	3.498475	2.413271	2.533074
C	2.046014	1.532364	-2.252613	Fe	-0.895775	0.342776	0.358835
H	1.614886	2.528547	-2.378097	C	-2.760984	-0.597455	0.358875
H	3.109441	1.562399	-2.505426	C	-3.621092	-0.472289	-0.750118
H	1.539511	0.855035	-2.943561	C	-3.205409	-1.414751	1.417015
C	2.658028	2.277521	0.545669	C	-4.841013	-1.153671	-0.821051
C	-0.152065	-2.786953	-1.575543	H	-3.333894	0.169287	-1.581512
C	0.874336	-3.848358	-1.998380	C	-4.421690	-2.102736	1.359133
H	0.508663	-4.357663	-2.898554	H	-2.585001	-1.517092	2.304568
H	1.847793	-3.410895	-2.234351	C	-5.240787	-1.979591	0.232976
H	1.019446	-4.610211	-1.225809	H	-5.479538	-1.040730	-1.694587
C	-1.524468	-3.459921	-1.383350	H	-4.732473	-2.732520	2.190104
H	-1.856977	-3.861984	-2.348046	H	-6.185814	-2.514316	0.181900
H	-1.471778	-4.296020	-0.679609	Br	-0.419229	0.615140	2.679631
H	-2.286131	-2.761508	-1.033074	C	-1.364682	2.076882	-0.650983
C	-0.249601	-1.697193	-2.657114	C	-1.877170	3.173998	0.067722
H	-1.027028	-0.963704	-2.418252	C	-1.314375	2.199262	-2.051686
H	0.702009	-1.170265	-2.779506	C	-2.304482	4.340364	-0.574910
H	-0.505208	-2.150571	-3.622113	H	-1.937003	3.123499	1.153687
C	1.595734	3.358212	0.837397	C	-1.745559	3.357353	-2.708507
H	2.047958	4.158525	1.436579	H	-0.923937	1.378719	-2.651354
H	1.206934	3.809772	-0.081256	C	-2.238809	4.434980	-1.968055
H	0.752798	2.948428	1.396780	H	-2.688725	5.174335	0.008383
C	3.858996	2.946195	-0.144737	H	-1.694367	3.421010	-3.793345
H	4.273526	3.705343	0.530689	H	-2.570568	5.339245	-2.471760

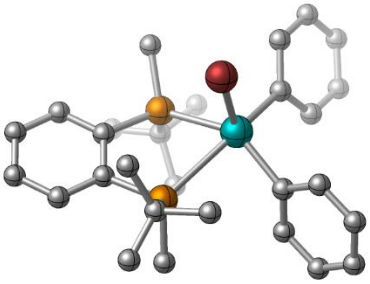

Zero-point correction= 0.587483 (Hartree/Particle)
Thermal correction to Energy= 0.624547
Thermal correction to Enthalpy= 0.625491

Thermal correction to Gibbs Free Energy= 0.517548
Sum of electronic and zero-point Energies= -5607.546839
Sum of electronic and thermal Energies= -5607.509775
Sum of electronic and thermal Enthalpies= -5607.508831
Sum of electronic and thermal Free Energies= -5607.616774

^{6}int-b

E(scf) = -5683.60303253 a.u.

ν_{min} = 28.5 cm^{-1}

C	2.505633	3.447139	-1.128184	H	4.865963	-1.761751	1.708029
H	3.142674	3.335337	-0.248877	H	4.283378	2.077082	3.562341
H	2.891092	2.781997	-1.907853	H	5.487679	-0.097481	3.450447
H	2.617832	4.479468	-1.489533	Br	1.432455	-0.280360	-2.231816
C	-2.158296	-1.043035	-0.074186	C	-2.103450	1.354712	-3.502523
C	-2.725440	-2.290611	0.245662	C	-1.319364	0.201452	2.485620
C	-2.813479	-0.211051	-1.016027	C	0.229882	-2.037579	1.274437
C	-4.046647	-0.633696	-1.541199	H	1.154426	-1.777641	1.791117
C	-3.929572	-2.705331	-0.318968	H	-0.358891	-2.702901	1.908803
H	-4.343032	-3.674615	-0.056416	H	0.485980	-2.558062	0.347973
C	-4.604583	-1.864116	-1.203214	C	-3.311529	2.617679	-1.124580
H	-5.555616	-2.165572	-1.632729	H	-3.010642	3.592371	-1.515931
H	-2.234424	-2.949378	0.952285	H	-4.302254	2.360308	-1.508960
H	-4.577957	0.007218	-2.235255	H	-3.362545	2.697410	-0.038626
P	-0.653439	-0.482001	0.846893	C	-2.208231	1.405323	2.138195
P	-2.017292	1.368209	-1.576060	H	-1.676421	2.115860	1.498318
Fe	0.983531	1.062415	-0.322248	H	-3.122387	1.094921	1.624107
C	0.491272	3.901915	0.301562	H	-2.500573	1.925099	3.057962
O	1.120578	4.397734	1.231231	C	-2.123105	-0.837200	3.281109
O	-0.874381	4.029772	0.229501	H	-2.508462	-0.365523	4.193295
C	-1.473224	4.809770	1.274662	H	-2.980716	-1.212506	2.715810
H	-2.548274	4.775736	1.093253	H	-1.508788	-1.689037	3.588395
H	-1.245477	4.398057	2.261527	C	-0.113881	0.683233	3.312850
H	-1.121643	5.845443	1.237692	H	-0.470174	1.053879	4.281281
C	1.047940	3.121979	-0.810828	H	0.609019	-0.114833	3.506545
H	0.403499	3.190361	-1.687423	H	0.413000	1.503153	2.818178
C	2.585981	0.681552	0.927042	C	-0.964304	2.295915	-3.951259
C	3.302847	-0.529339	0.871101	H	-0.957300	2.371940	-5.046201
C	2.974009	1.614019	1.909262	H	-1.092872	3.310022	-3.554900
C	4.335253	-0.814277	1.770764	H	0.011516	1.919396	-3.633215
H	3.045484	-1.266755	0.114993	C	-3.425342	1.878599	-4.090478
C	4.007618	1.340143	2.811360	H	-3.360758	1.852858	-5.185899
H	2.465227	2.573544	1.969994	H	-4.289249	1.272100	-3.805417
C	4.686686	0.120326	2.748596	H	-3.626633	2.914331	-3.802360

C	-1.828231	-0.059346	-4.041978	H	-2.643017	-0.752330	-3.815727
H	-0.900930	-0.469028	-3.636007	H	-1.722774	-0.012735	-5.133515

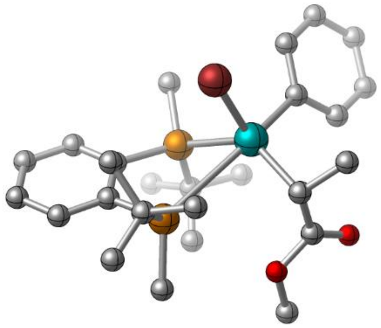

Zero-point correction= 0.608556 (Hartree/Particle)
Thermal correction to Energy= 0.647412
Thermal correction to Enthalpy= 0.648356
Thermal correction to Gibbs Free Energy= 0.537958
Sum of electronic and zero-point Energies= -5682.994476
Sum of electronic and thermal Energies= -5682.955621
Sum of electronic and thermal Enthalpies= -5682.954677
Sum of electronic and thermal Free Energies= -5683.065075

^{6}int-b-E
E(scf) = -5683.59375295 a.u.
ν_{min} =25.3 cm^{-1}

C	-2.507667	-0.445452	0.270743	C	2.651413	1.444700	1.902842
C	-3.535923	-1.259046	0.775962	C	0.468635	2.358997	2.328539
C	-2.841896	0.846015	-0.204771	C	3.012204	1.673900	3.234917
C	-4.171819	1.293140	-0.103322	H	3.378281	0.992192	1.230644
C	-4.850750	-0.807399	0.862736	C	0.820562	2.597163	3.661504
H	-5.621899	-1.458120	1.264660	H	-0.533736	2.630192	2.001875
C	-5.167498	0.481536	0.434472	C	2.094612	2.249410	4.118118
H	-6.185792	0.852018	0.505409	H	4.006129	1.403407	3.583939
H	-3.307395	-2.263826	1.107414	H	0.103153	3.049601	4.342241
H	-4.447262	2.281084	-0.453496	H	2.371596	2.429124	5.153601
P	-0.735477	-0.984254	0.240220	C	1.079353	4.607704	-0.943214
P	-1.602564	1.900489	-1.079327	O	0.861607	5.080814	0.166958
Fe	0.841936	1.559898	-0.542481	O	0.574852	5.222613	-2.067929
C	-0.779333	-2.389871	-0.963870	C	-0.065309	6.486116	-1.841533
C	-0.363050	-1.862803	1.896531	C	-0.933485	-3.284652	2.044135
C	-2.008541	1.741442	-2.924223	H	-2.025334	-3.308347	2.074562
C	-2.032851	3.622974	-0.610928	H	-0.579785	-3.708364	2.992872
C	1.843953	3.393386	-1.256867	H	-0.597632	-3.952978	1.246401
C	1.369755	1.769024	1.425998	C	1.177897	-1.955077	1.934812

H	1.492161	-2.428637	2.873384	H	-3.443253	3.401513	-3.015113
H	1.644815	-0.969849	1.878092	H	-4.195334	1.811513	-2.760115
H	1.569644	-2.562881	1.111590	H	-1.595950	3.827115	0.368813
C	-0.858080	-0.981071	3.054136	H	-3.106189	3.815084	-0.584801
H	-1.950660	-0.920265	3.079572	H	-1.572306	4.290431	-1.337429
H	-0.459331	0.034659	2.990564	H	-0.899159	-1.997495	-1.974227
H	-0.524741	-1.408882	4.007863	H	0.174384	-2.922677	-0.923034
C	-1.965796	0.251876	-3.299754	H	-1.593871	-3.089769	-0.756130
H	-2.718349	-0.332315	-2.762168	H	0.641177	7.211333	-1.425847
H	-0.978852	-0.177022	-3.101201	H	-0.911263	6.395254	-1.154274
H	-2.161498	0.146428	-4.373199	H	-0.412353	6.823725	-2.819687
C	-0.898044	2.482346	-3.689804	Br	2.137997	-0.037931	-1.747096
H	-0.802282	3.531135	-3.395999	C	2.474127	3.344881	-2.647196
H	-1.117606	2.450519	-4.763489	H	1.727942	3.336435	-3.443258
H	0.071194	2.000192	-3.535399	H	3.118443	4.218726	-2.822418
C	-3.381634	2.338046	-3.266027	H	3.084966	2.446563	-2.754332
H	-3.550182	2.244529	-4.345622	H	2.612123	3.294763	-0.482155

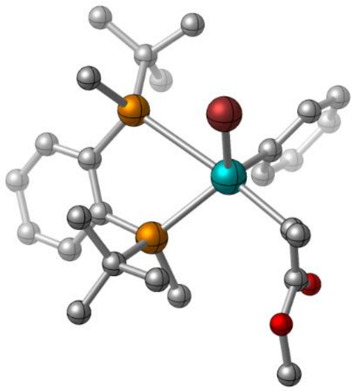

Zero-point correction= 0.610494 (Hartree/Particle)
Thermal correction to Energy= 0.649216
Thermal correction to Enthalpy= 0.650160
Thermal correction to Gibbs Free Energy= 0.540076
Sum of electronic and zero-point Energies= -5682.983259
Sum of electronic and thermal Energies= -5682.944537
Sum of electronic and thermal Enthalpies= -5682.943593
Sum of electronic and thermal Free Energies= -5683.053676

^{6}TS2

E(scf) = -5608.10129806 a.u.

ν_{min} = -167.1 cm^{-1}

C	-2.502958	-0.896361	-0.334490	H	0.106476	4.757145	1.087253
C	-3.446932	-1.935802	-0.373390	C	1.840558	5.805374	-1.659529
C	-2.881815	0.397827	-0.765792	H	3.422053	4.695015	-2.623519
C	-4.220470	0.628707	-1.124443	H	0.253955	6.686882	-0.488760
C	-4.761585	-1.697396	-0.769247	H	1.906631	6.645525	-2.345433
H	-5.477358	-2.513970	-0.791376	H	-3.360166	3.384718	-0.593454
C	-5.155587	-0.404891	-1.120074	H	-1.719762	4.069801	-0.478509
H	-6.184233	-0.204248	-1.405001	H	-2.366038	3.087129	0.848439
H	-3.155895	-2.941375	-0.089817	Br	2.090043	0.054930	-1.650133
H	-4.541131	1.621479	-1.419739	C	-0.278640	2.957299	-3.042491
P	-0.809281	-1.146411	0.352949	H	-0.155560	3.167735	-4.111873
P	-1.582005	1.701035	-0.976013	H	-0.406140	3.914728	-2.528142
Fe	0.526201	0.886882	-0.021685	H	0.644499	2.498976	-2.680690
C	-0.215730	-2.690604	-0.445637	C	-2.748582	2.685519	-3.428146
C	-1.045364	-1.545834	2.189586	H	-2.577881	2.892177	-4.492050
C	-1.491314	2.026294	-2.842888	H	-3.625535	2.036631	-3.362214
C	-2.342173	3.205775	-0.238617	H	-2.981036	3.639259	-2.944149
C	1.441613	1.842130	1.550766	C	-1.234511	0.676247	-3.537855
C	2.805808	1.539790	1.837328	H	-1.082511	0.845820	-4.610682
C	0.710283	2.436304	2.620273	H	-0.335428	0.188562	-3.149239
C	3.393195	1.835980	3.064978	H	-2.081754	-0.007528	-3.423132
H	3.407509	1.068834	1.062427	H	0.782403	-2.921841	-0.065824
C	1.290264	2.738734	3.849872	H	-0.878040	-3.543774	-0.278246
H	-0.339652	2.684320	2.468254	H	-0.134402	-2.503314	-1.519203
C	2.640020	2.441447	4.080509	C	0.373646	-1.609210	2.790916
H	4.438487	1.590834	3.241886	H	0.304511	-1.832756	3.862312
H	0.694829	3.196898	4.637061	H	0.978895	-2.395530	2.327070
H	3.096398	2.671725	5.039547	H	0.902414	-0.657886	2.679975
C	1.662523	3.657141	0.076805	C	-1.816790	-0.369999	2.813930
C	2.610350	3.619810	-0.934783	H	-1.303605	0.581587	2.644671
C	0.820336	4.741034	0.267196	H	-2.833599	-0.291978	2.415759
C	2.693544	4.707512	-1.815802	H	-1.890301	-0.513907	3.898497
H	3.256376	2.756649	-1.061701	C	-1.785551	-2.862143	2.465766
C	0.908053	5.827024	-0.617116	H	-1.825781	-3.030104	3.549261

H	-2.816278	-2.837580	2.102787	H	-1.278706	-3.723925	2.020419

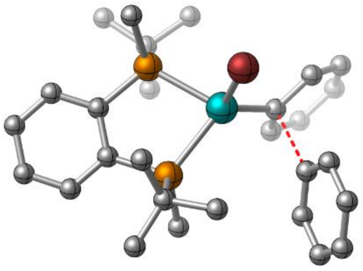

Zero-point correction= 0.584653 (Hartree/Particle)
Thermal correction to Energy= 0.621860
Thermal correction to Enthalpy= 0.622804
Thermal correction to Gibbs Free Energy= 0.513901
Sum of electronic and zero-point Energies= -5607.516645
Sum of electronic and thermal Energies= -5607.479438
Sum of electronic and thermal Enthalpies= -5607.478494
Sum of electronic and thermal Free Energies= -5607.587397

^{6}TS7

E(scf) = -3343.45340327 a.u.

ν_{min} = -159.6 cm^{-1}

C	0.424874	4.609850	-1.559587	C	0.273804	-1.482042	2.165243
H	-0.530056	5.003545	-1.914695	C	-2.535147	0.257734	-3.051507
H	0.336777	4.359409	-0.501125	C	-2.535874	2.775890	-1.483926
H	1.158261	5.430475	-1.629066	H	-2.087018	3.287754	-2.338398
C	-2.254302	-0.891857	0.576202	H	-3.624033	2.829506	-1.564264
C	-3.059453	-1.656872	1.435764	H	-2.216493	3.285905	-0.572080
C	-2.838885	0.191979	-0.126625	C	0.412159	3.372287	-3.751336
C	-4.181746	0.520552	0.124357	O	-0.540094	3.993649	-4.237028
C	-4.396075	-1.330868	1.656651	O	1.170809	2.520371	-4.517072
H	-4.995877	-1.932957	2.332874	C	0.845476	2.474635	-5.909020
C	-4.952541	-0.223179	1.015935	H	1.503009	1.718593	-6.342286
H	-5.987476	0.052556	1.195913	H	-0.199109	2.198973	-6.075322
H	-2.636773	-2.513085	1.947856	H	1.028889	3.443140	-6.387232
H	-4.640733	1.357746	-0.389467	C	0.865606	3.445971	-2.391134
P	-0.434552	-1.176978	0.416550	H	1.816795	2.976910	-2.166615
P	-1.880846	1.059041	-1.454472	C	1.069567	2.110807	0.563855
Fe	0.564213	0.830913	-0.959133	C	2.393484	2.556019	0.743056
C	-0.270924	-2.832323	-0.383086	C	0.105265	2.650744	1.440057
H	0.776058	-3.141634	-0.321275	C	2.741627	3.484540	1.730766
H	-0.901139	-3.587343	0.094384	H	3.177319	2.177982	0.087089
H	-0.536861	-2.760026	-1.437086	C	0.433828	3.580975	2.432373

H	-0.935867	2.342499	1.346084	C	1.789857	-1.230303	2.021534
C	1.758992	4.001593	2.579405	H	2.283637	-1.418282	2.982790
H	3.774929	3.808031	1.837704	H	2.245702	-1.895103	1.279924
H	-0.338231	3.978263	3.088001	H	1.996829	-0.196948	1.730637
H	2.022273	4.725240	3.346643	C	0.062500	-2.912011	2.695295
C	1.961016	-0.193720	-2.072822	H	0.528282	-2.991214	3.685490
C	3.150560	-0.594331	-1.424981	H	-0.992584	-3.170596	2.815204
C	1.827088	-0.599775	-3.417394	H	0.529664	-3.666146	2.056338
C	4.136678	-1.355021	-2.063399	C	-2.123114	-1.221982	-3.039333
H	3.315171	-0.310067	-0.386584	H	-2.463048	-1.702013	-3.964712
C	2.800091	-1.363709	-4.070855	H	-1.035354	-1.329088	-2.991472
H	0.944924	-0.306771	-3.981125	H	-2.567062	-1.765387	-2.200018
C	3.960851	-1.746453	-3.392975	C	-4.059192	0.372342	-3.201678
H	5.038449	-1.642461	-1.526619	H	-4.355734	-0.073206	-4.159311
H	2.656078	-1.657903	-5.108571	H	-4.592750	-0.162022	-2.411123
H	4.720679	-2.338997	-3.896461	H	-4.397496	1.412943	-3.208336
C	-0.319653	-0.470940	3.159191	C	-1.842503	0.963391	-4.227589
H	-0.180479	0.559115	2.823856	H	-2.129753	0.46833	-5.163281
H	-1.387484	-0.638201	3.326237	H	-2.115462	2.017406	-4.308484
H	0.190325	-0.576289	4.124624	H	-0.755416	0.90934	-4.139515

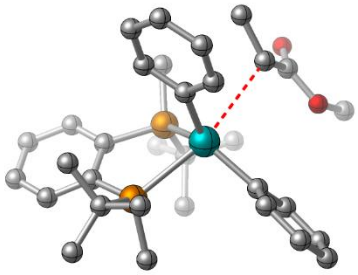

Zero-point correction= 0.692354 (Hartree/Particle)
Thermal correction to Energy= 0.735665
Thermal correction to Enthalpy= 0.736609
Thermal correction to Gibbs Free Energy= 0.614805
Sum of electronic and zero-point Energies= -3342.761049
Sum of electronic and thermal Energies= -3342.717739
Sum of electronic and thermal Enthalpies= -3342.716794
Sum of electronic and thermal Free Energies= -3342.838598

^{6}TS8
E(scf) = -3343.44037626 a.u.
ν_{min} = -309.5 cm^{-1}

C	2.609121	3.144563	-1.907661	H	2.621186	2.073553	-2.126680
H	3.503624	3.394183	-1.332105	H	2.670877	3.681058	-2.865454

C	-2.725874	-0.891983	0.083435	H	2.433150	-1.413430	-0.081303
C	-3.691920	-1.806121	0.536469	C	2.865496	-1.179824	-3.914198
C	-3.155154	0.295438	-0.556491	H	1.272307	0.240686	-3.886002
C	-4.530464	0.574598	-0.631078	C	3.688799	-2.030978	-3.172727
C	-5.053491	-1.530333	0.426173	H	4.157784	-2.758773	-1.196569
H	-5.782242	-2.249254	0.789113	H	2.986160	-1.113565	-4.993723
C	-5.473988	-0.323621	-0.136354	H	4.449588	-2.629543	-3.667499
H	-6.532414	-0.089438	-0.203806	C	-0.609637	-2.820213	-0.331062
H	-3.381370	-2.744138	0.982645	H	-1.319644	-3.569923	0.027300
H	-4.871056	1.498868	-1.085531	H	-0.690659	-2.742174	-1.418445
P	-0.918700	-1.153095	0.388061	H	0.408103	-3.136634	-0.090887
P	-1.908374	1.397949	-1.358127	C	-2.520629	3.094940	-0.973802
Fe	0.384749	0.658991	-0.704188	H	-2.709369	3.155797	0.101245
C	-0.703016	-1.358550	2.263814	H	-1.753324	3.831998	-1.218196
C	-2.170306	1.166495	-3.219948	H	-3.445336	3.341105	-1.502162
C	1.407078	4.796794	-0.436512	C	-1.413846	-0.180732	2.951291
O	2.409658	5.383846	-0.039444	H	-2.500304	-0.237663	2.831155
O	0.141751	5.259754	-0.165444	H	-1.192283	-0.196277	4.025368
C	0.079927	6.371250	0.733554	H	-1.070582	0.782055	2.560198
H	-0.981496	6.581589	0.879331	C	0.817378	-1.255988	2.504187
H	0.546253	6.125309	1.692706	H	1.217941	-0.295875	2.165733
H	0.579022	7.251515	0.315864	H	1.025610	-1.348190	3.576967
C	1.369472	3.534477	-1.151845	H	1.363767	-2.056435	1.992533
H	0.428221	3.344476	-1.655549	C	-1.215794	-2.689281	2.835214
C	1.218252	2.197090	0.495023	H	-2.299825	-2.791703	2.743170
C	2.554263	1.933735	0.948777	H	-0.742901	-3.553984	2.359510
C	0.356278	2.776544	1.488786	H	-0.977802	-2.733932	3.905437
C	2.985624	2.229346	2.236815	C	-1.145109	2.097970	-3.896016
H	3.257646	1.474677	0.257079	H	-1.162475	1.941303	-4.981106
C	0.782453	3.070088	2.777693	H	-1.372177	3.153070	-3.709302
H	-0.670279	3.006174	1.217854	H	-0.127199	1.904421	-3.544094
C	2.106039	2.803155	3.168005	C	-1.845114	-0.305111	-3.534069
H	4.007944	1.999524	2.531334	H	-2.575950	-0.981015	-3.077949
H	0.083024	3.493129	3.496501	H	-1.870560	-0.466298	-4.618400
H	2.440337	3.030145	4.176462	H	-0.850377	-0.587738	-3.176979
C	1.679907	-0.455783	-1.873371	C	-3.581613	1.499798	-3.721649
C	2.536992	-1.329068	-1.162980	H	-3.611404	1.380365	-4.811918
C	1.887949	-0.412728	-3.269748	H	-4.33386	0.828738	-3.298636
C	3.522328	-2.101619	-1.787049	H	-3.871283	2.531794	-3.500496

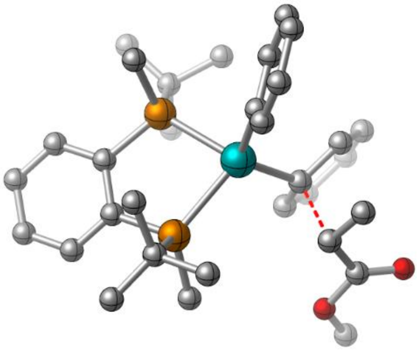

Zero-point correction= 0.692009 (Hartree/Particle)
Thermal correction to Energy= 0.735025
Thermal correction to Enthalpy= 0.735969
Thermal correction to Gibbs Free Energy= 0.615718
Sum of electronic and zero-point Energies= -3342.748367
Sum of electronic and thermal Energies= -3342.705351
Sum of electronic and thermal Enthalpies= -3342.704407
Sum of electronic and thermal Free Energies= -3342.824658

⁶TS8'

E(scf) = -3343.42463295 a.u.

ν_{min} = -157.3 cm^{-1}

C	-1.790575	-1.133625	0.694662	H	1.483420	-1.291640	-2.740092
C	-2.473315	-2.082266	1.474618	C	4.841269	-0.867173	-2.521089
C	-2.526161	-0.068308	0.115417	H	5.818276	0.759037	-1.490794
C	-3.900857	0.037864	0.389754	H	3.571593	-2.362748	-3.423242
C	-3.838620	-1.967768	1.726854	H	5.767948	-1.346183	-2.826530
H	-4.339562	-2.710174	2.341327	C	1.775055	1.960533	-3.407577
C	-4.553009	-0.893785	1.194076	C	1.489999	3.285348	-3.109628
H	-5.615161	-0.786974	1.394379	C	1.656878	1.440215	-4.686573
H	-1.934028	-2.921536	1.896392	C	1.059062	4.125699	-4.148104
H	-4.473984	0.854275	-0.036098	H	1.568739	3.676715	-2.098662
P	0.044581	-1.201123	0.452532	C	1.223356	2.285927	-5.718171
P	-1.701274	1.146808	-1.016598	H	1.890639	0.397338	-4.888015
Fe	0.767522	0.990233	-0.530689	C	0.924268	3.625219	-5.447167
C	0.889763	-1.648499	2.100184	H	0.824643	5.166913	-3.937699
C	-2.253308	0.637426	-2.763404	H	1.118276	1.899088	-6.729614
C	2.387637	0.405351	-1.724984	H	0.585599	4.277484	-6.247489
C	3.673029	0.926488	-1.384312	C	1.031192	2.363873	1.130320
C	2.419977	-0.815226	-2.460949	C	-0.286384	2.662565	1.852974
C	4.864665	0.317014	-1.773663	H	-0.825680	3.450824	1.323551
H	3.722814	1.836709	-0.794019	H	-0.947760	1.791551	1.914429
C	3.604976	-1.432548	-2.858809	H	-0.126782	3.015911	2.883114

C	1.662403	3.563626	0.593992	C	0.334024	-0.748823	3.215264
O	1.106106	4.552982	0.113758	H	0.441191	0.310545	2.973250
O	3.038811	3.495048	0.626726	H	-0.723482	-0.949506	3.412864
C	3.731401	4.563517	-0.030177	H	0.889388	-0.933805	4.142765
H	4.793901	4.380555	0.142485	C	0.783291	-3.125006	2.519144
H	3.531872	4.567218	-1.106698	H	1.360746	-3.271396	3.440490
H	3.447682	5.535585	0.383459	H	-0.242272	-3.434865	2.733241
H	1.742416	1.835544	1.773722	H	1.197674	-3.801255	1.766500
C	-2.634101	2.713163	-0.721925	C	-1.932668	1.823178	-3.692175
H	-2.736760	2.877922	0.351235	H	-2.085377	1.517199	-4.733793
H	-2.057956	3.539268	-1.145077	H	-2.587188	2.679141	-3.501112
H	-3.628813	2.705452	-1.174966	H	-0.897153	2.152420	-3.592041
C	0.271219	-2.729988	-0.555389	C	-3.744070	0.286247	-2.867987
H	1.337789	-2.950877	-0.638869	H	-4.002391	-0.592873	-2.271436
H	-0.245253	-3.582138	-0.105527	H	-4.390438	1.113441	-2.558482
H	-0.123526	-2.571736	-1.559213	H	-3.983715	0.060821	-3.914734
C	2.378801	-1.315654	1.858200	C	-1.40922	-0.581361	-3.161224
H	2.956210	-1.538923	2.763431	H	-0.341279	-0.351825	-3.122116
H	2.800882	-1.907020	1.038608	H	-1.611555	-1.431109	-2.5022
H	2.521176	-0.259814	1.614308	H	-1.65051	-0.887864	-4.186111

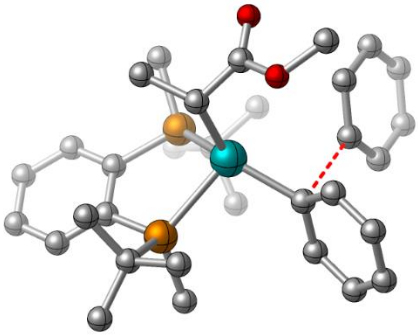

Zero-point correction= 0.692159 (Hartree/Particle)
Thermal correction to Energy= 0.735192
Thermal correction to Enthalpy= 0.736137
Thermal correction to Gibbs Free Energy= 0.616434
Sum of electronic and zero-point Energies= -3342.732474
Sum of electronic and thermal Energies= -3342.689441
Sum of electronic and thermal Enthalpies= -3342.688496
Sum of electronic and thermal Free Energies= -3342.808199

^{6}TS-b
E(scf) = -5683.59185230 a.u.
ν_{min} = -175.2 cm^{-1}

C	-2.747484	-0.730525	0.009501	H	2.309537	2.486092	-3.148488
C	-3.749166	-1.615752	0.440146	H	3.189209	2.774363	-1.640535
C	-3.124575	0.515560	-0.549563	C	-1.255687	-2.977971	2.411755
C	-4.485313	0.866855	-0.568413	H	-2.347122	-2.949470	2.450280
C	-5.095592	-1.260410	0.394549	H	-0.906819	-3.230116	3.421058
H	-5.852387	-1.958304	0.740465	H	-0.949598	-3.792899	1.749104
C	-5.462122	-0.003901	-0.088836	C	0.889862	-1.712239	2.131389
H	-6.506770	0.292011	-0.112011	H	1.154394	-1.925620	3.173931
H	-3.476594	-2.595700	0.813158	H	1.374123	-0.776235	1.844849
H	-4.796573	1.823096	-0.973109	H	1.306964	-2.514789	1.513737
P	-0.951767	-1.104928	0.202313	C	-1.188189	-0.515965	2.917268
P	-1.853195	1.589449	-1.364618	H	-2.276539	-0.423952	2.838990
Fe	0.423578	1.031726	-0.446999	H	-0.738590	0.453424	2.687507
C	-0.705003	-2.654389	-0.762758	H	-0.945963	-0.750955	3.960779
C	-0.646405	-1.626836	2.002895	C	-1.744137	-0.312972	-3.416585
C	-2.057007	1.179334	-3.218257	H	-2.450658	-0.952707	-2.879473
C	-2.528230	3.289498	-1.169156	H	-0.727938	-0.552926	-3.092343
C	1.168939	3.513904	-1.639668	H	-1.818483	-0.557184	-4.482956
H	0.253703	3.504599	-2.212369	C	-1.030746	2.018473	-4.000817
C	1.311120	1.829361	1.198105	H	-1.202185	3.093369	-3.883015
C	2.666620	1.580263	1.489535	H	-1.121018	1.787815	-5.069094
C	0.595463	2.602732	2.133740	H	-0.005494	1.788233	-3.697953
C	3.274302	2.064515	2.652477	C	-3.471623	1.482571	-3.734864
H	3.260896	0.987849	0.795833	H	-3.521198	1.223596	-4.799540
C	1.185523	3.085433	3.305794	H	-3.729356	2.542136	-3.646170
H	-0.448955	2.845061	1.943009	H	-4.234114	0.893269	-3.218474
C	2.532298	2.816817	3.566783	H	-3.522519	3.404117	-1.605314
H	4.323844	1.855372	2.847578	H	-1.848018	4.006257	-1.627748
H	0.601298	3.672172	4.011430	H	-2.575230	3.520221	-0.102540
H	2.999514	3.193090	4.473305	H	-0.761867	-2.429135	-1.828011
C	1.221007	4.459524	-0.545931	H	0.293006	-3.047131	-0.554723
O	2.240971	4.925815	-0.048284	H	-1.454154	-3.411462	-0.515157
O	-0.028405	4.836463	-0.121796	H	0.502512	5.434390	1.809618
C	-0.048885	5.797064	0.938503	H	-1.100996	5.940286	1.193022
C	2.455988	3.196915	-2.334320	H	0.386792	6.749065	0.616813
H	2.906874	4.111920	-2.751578	Br	1.858673	-0.214827	-1.895186

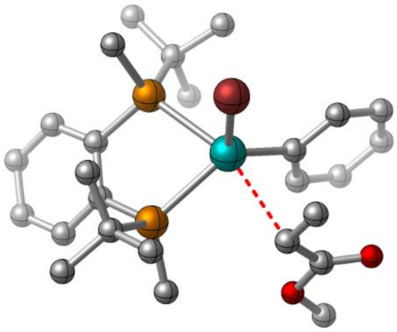

Zero-point correction=	0.603982 (Hartree/Particle)
Thermal correction to Energy=	0.643540
Thermal correction to Enthalpy=	0.644484
Thermal correction to Gibbs Free Energy=	0.531420
Sum of electronic and zero-point Energies=	-5682.987870
Sum of electronic and thermal Energies=	-5682.948312
Sum of electronic and thermal Enthalpies=	-5682.947368
Sum of electronic and thermal Free Energies=	-5683.060432

^{6}TS-b-E

E(scf) = -5683.58887008 a.u.

ν_{min} = -177.1 cm^{-1}

C	-2.587739	-0.475578	0.190787
C	-3.622138	-1.314751	0.636043
C	-2.906299	0.830431	-0.257471
C	-4.239315	1.268571	-0.176743
C	-4.939989	-0.867840	0.698933
H	-5.721513	-1.532872	1.054434
C	-5.246011	0.435646	0.307275
H	-6.267395	0.800361	0.362569
H	-3.399035	-2.331762	0.934379
H	-4.508783	2.265779	-0.504970
P	-0.818930	-0.998490	0.205550
P	-1.605960	1.879380	-1.062408
Fe	0.645453	1.146350	-0.301825
C	-0.759266	-2.417233	-0.970566
C	-0.429031	-1.799448	1.885797
C	-1.947229	1.689364	-2.922995
C	-2.081382	3.611337	-0.672729
C	1.774852	3.463800	-1.315022
C	1.431807	1.711542	1.480365
C	2.770699	1.442783	1.825837
C	0.660606	2.408234	2.430340
C	3.309644	1.832054	3.056899
H	3.408878	0.910766	1.121587
C	1.182272	2.798548	3.666903
H	-0.376267	2.653316	2.204814
C	2.512919	2.509848	3.983142
H	4.347045	1.606752	3.294327
H	0.557703	3.331774	4.380245
H	2.925603	2.813743	4.941831
C	1.002345	4.594219	-0.851707
O	0.790692	4.910031	0.317837
O	0.488396	5.349829	-1.886286
C	-0.164116	6.562291	-1.490133
C	-1.032014	-3.198440	2.098776
H	-2.122212	-3.183038	2.165300
H	-0.659848	-3.598186	3.050418
H	-0.740768	-3.903154	1.314922
C	1.110426	-1.918127	1.911210
H	1.424936	-2.334894	2.875613
H	1.594407	-0.947362	1.788112
H	1.481998	-2.584400	1.125250
C	-0.901928	-0.854458	3.001731
H	-1.992567	-0.760742	3.016194
H	-0.469276	0.143812	2.899201

H	-0.586479	-1.252525	3.973768	H	-3.160090	3.773396	-0.661810
C	-1.833177	0.201522	-3.289340	H	-1.639336	4.251377	-1.435306
H	-2.573526	-0.411148	-2.766686	H	-0.846369	-2.049966	-1.992775
H	-0.832846	-0.182554	-3.066973	H	0.207774	-2.916358	-0.872729
H	-2.000421	0.081812	-4.366215	H	-1.561587	-3.134810	-0.778201
C	-0.848442	2.467992	-3.665978	H	0.532647	7.231508	-0.975227
H	-0.811735	3.523919	-3.384383	H	-1.011489	6.369005	-0.825891
H	-1.038082	2.414759	-4.744857	H	-0.512928	7.028688	-2.413421
H	0.134768	2.032510	-3.475914	Br	2.072473	-0.071977	-1.780443
C	-3.333952	2.223849	-3.310608	C	2.327354	3.422033	-2.710553
H	-3.466762	2.114442	-4.393835	H	1.615189	3.781074	-3.454336
H	-3.450077	3.285614	-3.072022	H	3.219086	4.066672	-2.785483
H	-4.139350	1.667125	-2.823882	H	2.637588	2.408756	-2.975298
H	-1.655902	3.894088	0.291786	H	2.415191	3.058635	-0.538376

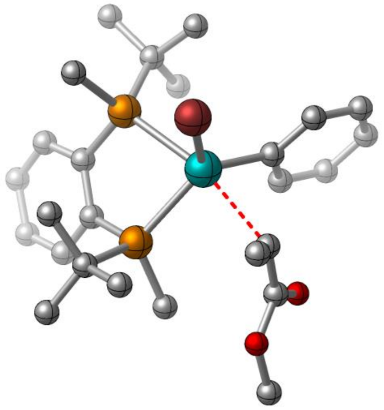

Zero-point correction= 0.604947 (Hartree/Particle)
Thermal correction to Energy= 0.644082
Thermal correction to Enthalpy= 0.645026
Thermal correction to Gibbs Free Energy= 0.533704
Sum of electronic and zero-point Energies= -5682.983924
Sum of electronic and thermal Energies= -5682.944788
Sum of electronic and thermal Enthalpies= -5682.943844
Sum of electronic and thermal Free Energies= -5683.055166

^{6}TS-c
E(scf) = -5683.58065418 a.u.
ν_{min} = -352.4 cm^{-1}

C	2.690382	2.941234	-1.793931	C	-2.681387	-0.829292	0.079966
H	3.661193	3.048566	-1.304007	C	-3.733929	-1.611684	0.582048
H	2.555910	1.892090	-2.066968	C	-2.979784	0.254437	-0.782231
H	2.720311	3.530496	-2.721528	C	-4.324930	0.558939	-1.052238

C	-5.061544	-1.308287	0.288219	H	3.428195	1.283702	0.467568
H	-5.860021	-1.923839	0.691947	C	0.950683	2.930692	2.962975
C	-5.358369	-0.207630	-0.517324	H	-0.493250	2.865236	1.392716
H	-6.390622	0.046774	-0.739007	C	2.270410	2.653069	3.361305
H	-3.516069	-2.468465	1.209002	H	4.169713	1.822963	2.748784
H	-4.575463	1.399001	-1.690771	H	0.252029	3.365701	3.674858
P	-0.924857	-1.149787	0.554476	H	2.600035	2.883431	4.370432
P	-1.598802	1.175683	-1.599257	C	-0.438790	1.391587	-4.078439
Fe	0.496606	0.576505	-0.478166	H	-0.364485	1.085915	-5.128785
C	-0.624150	-2.853727	-0.069576	H	-0.547588	2.480871	-4.058913
H	0.372693	-3.178874	0.237538	H	0.503575	1.130571	-3.586671
H	-1.373431	-3.561542	0.294861	C	-2.939363	1.079843	-4.142372
H	-0.652585	-2.829457	-1.161217	H	-2.861168	0.805541	-5.201645
C	-0.818312	-1.268987	2.443987	H	-3.808676	0.557745	-3.733224
C	-1.642045	0.680766	-3.425109	H	-3.125506	2.157460	-4.096230
C	-2.144374	2.933672	-1.551891	C	-1.453531	-0.845738	-3.495738
H	-1.485068	3.531906	-2.185083	H	-0.516071	-1.156691	-3.026068
H	-3.173160	3.068122	-1.893874	H	-2.282130	-1.376553	-3.015649
H	-2.060963	3.303783	-0.526551	H	-1.419005	-1.158830	-4.546046
C	1.853180	4.643816	-0.138668	C	0.685014	-1.101615	2.756006
O	2.958370	5.080970	0.167980	H	0.852098	-1.231802	3.831829
O	0.694058	5.244681	0.274098	H	1.295572	-1.845217	2.231140
C	0.865682	6.316638	1.207313	H	1.047747	-0.109787	2.475742
H	-0.139871	6.655231	1.464669	C	-1.310274	-2.601412	3.031293
H	1.385000	5.972866	2.107305	H	-1.162954	-2.585450	4.118296
H	1.434313	7.141850	0.766993	H	-2.374917	-2.773544	2.853611
C	1.589784	3.424275	-0.889747	H	-0.751163	-3.456023	2.639044
H	0.584077	3.365805	-1.290197	C	-1.607778	-0.098032	3.052274
C	1.401510	2.044130	0.689259	H	-1.296122	0.862977	2.631588
C	2.731725	1.758269	1.154806	H	-2.685192	-0.210502	2.897651
C	0.531586	2.632289	1.674742	H	-1.423619	-0.057146	4.132562
C	3.153264	2.060741	2.441579	Br	1.985465	-0.841544	-1.723038

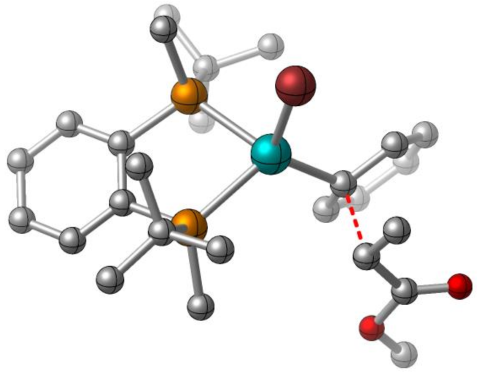

Zero-point correction= 0.603600 (Hartree/Particle)
Thermal correction to Energy= 0.643121
Thermal correction to Enthalpy= 0.644066
Thermal correction to Gibbs Free Energy= 0.530133
Sum of electronic and zero-point Energies= -5682.977055
Sum of electronic and thermal Energies= -5682.937533
Sum of electronic and thermal Enthalpies= -5682.936589
Sum of electronic and thermal Free Energies= -5683.050521

^{6}TS-c-E
E(scf) = -5683.57235643 a.u.
ν_{min} = cm^{-1}

C	-2.680867	-1.042070	0.081172	H	-3.668152	2.901276	-1.957960
C	-3.612390	-1.964062	0.585953	H	-2.772798	3.106921	-0.434075
C	-3.151102	0.070664	-0.662265	C	2.701009	4.362019	0.009603
C	-4.535759	0.275100	-0.780514	O	3.774628	4.245825	0.593851
C	-4.983043	-1.758888	0.438245	O	1.926356	5.485055	0.133651
H	-5.684843	-2.481505	0.844011	C	2.401743	6.448272	1.078428
C	-5.446326	-0.621522	-0.224023	H	1.678192	7.265934	1.064879
H	-6.512489	-0.442305	-0.326806	H	2.456506	6.016484	2.082897
H	-3.267041	-2.854245	1.098229	H	3.392407	6.823802	0.803304
H	-4.913475	1.134161	-1.324200	C	2.064384	3.338619	-0.803707
P	-0.869858	-1.235409	0.390295	C	1.028342	2.268261	0.726398
P	-1.955422	1.143603	-1.575927	C	2.052343	1.854170	1.645968
Fe	0.249261	0.789457	-0.510629	C	0.063841	3.180163	1.279956
C	-0.456563	-2.864734	-0.348916	C	2.113886	2.302430	2.956546
H	0.599163	-3.081542	-0.170913	H	2.822489	1.172505	1.289100
H	-1.072247	-3.671626	0.058385	C	0.127225	3.640339	2.589199
H	-0.611509	-2.801856	-1.427925	H	-0.740654	3.537701	0.641222
C	-0.590386	-1.400634	2.251420	C	1.152218	3.204422	3.444855
C	-2.088806	0.585360	-3.381853	H	2.905882	1.950839	3.614602
C	-2.684889	2.828911	-1.487418	H	-0.626960	4.331958	2.960008
H	-2.006690	3.532342	-1.975754	H	1.199120	3.556744	4.471572

C	-1.058756	1.412620	-4.175933	H	1.482712	-0.784352	1.850734
H	-1.094334	1.114048	-5.230586	C	-1.291862	-2.583345	2.934335
H	-1.270025	2.486335	-4.130413	H	-0.924568	-2.666253	3.964761
H	-0.040919	1.237419	-3.814663	H	-2.374001	-2.442633	2.988059
C	-3.490872	0.781826	-3.975383	H	-1.085240	-3.535693	2.435553
H	-3.484777	0.449225	-5.020589	C	-1.049056	-0.076145	2.886351
H	-4.245599	0.191650	-3.448193	H	-0.511969	0.779467	2.468618
H	-3.801730	1.831179	-3.969083	H	-2.123191	0.086419	2.744916
C	-1.701241	-0.903427	-3.430084	H	-0.852190	-0.098187	3.964552
H	-0.696051	-1.066164	-3.029743	Br	1.903877	-0.207735	-1.999181
H	-2.410419	-1.525367	-2.875033	C	1.036628	3.727459	-1.829672
H	-1.703123	-1.243058	-4.472613	H	0.217463	4.296471	-1.383622
C	0.937989	-1.555360	2.403248	H	1.478374	4.354610	-2.616978
H	1.209277	-1.460043	3.461003	H	0.626784	2.842754	-2.322495
H	1.283236	-2.535074	2.057561	H	2.744926	2.543191	-1.087247

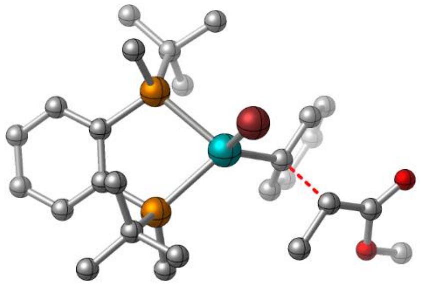

Zero-point correction= 0.603580 (Hartree/Particle)
Thermal correction to Energy= 0.643003
Thermal correction to Enthalpy= 0.643947
Thermal correction to Gibbs Free Energy= 0.531325
Sum of electronic and zero-point Energies= -5682.968776
Sum of electronic and thermal Energies= -5682.929354
Sum of electronic and thermal Enthalpies= -5682.928410
Sum of electronic and thermal Free Energies= -5683.041032

Ph-Ph
E(scf) = -463.349974591 a.u.
ν_{min} = 72.1 cm^{-1}

C	0.585817	2.369801	0.010182	H	2.344759	1.548356	-0.930749
C	1.875711	2.438842	-0.521551	H	3.584174	3.682153	-0.915094
C	2.572869	3.646880	-0.520762	H	0.219754	5.622042	0.935999
C	1.995109	4.813560	0.010621	H	-1.006421	3.480561	0.950833
C	0.695866	4.729123	0.541723	C	2.736942	6.101071	0.010625
C	-0.000550	3.520682	0.542148	C	3.558662	6.460839	-1.072220
H	0.042985	1.428971	0.009958	C	2.635563	6.992994	1.092977

C	4.254708	7.669477	-1.072537	C	4.143650	8.546221	0.009264
H	3.633789	5.798757	-1.929745	H	4.877891	7.930181	-1.923488
C	3.330533	8.202283	1.091817	H	3.243152	8.872960	1.942099
H	2.025700	6.726137	1.951095	H	4.685487	9.487624	0.008779

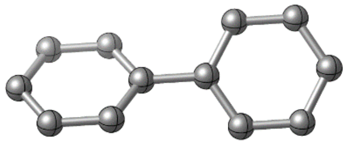

Zero-point correction= 0.181878 (Hartree/Particle)
Thermal correction to Energy= 0.190723
Thermal correction to Enthalpy= 0.191668
Thermal correction to Gibbs Free Energy= 0.147374
Sum of electronic and zero-point Energies= -463.168097
Sum of electronic and thermal Energies= -463.159251
Sum of electronic and thermal Enthalpies= -463.158307
Sum of electronic and thermal Free Energies= -463.202601

Ph-R
E(scf) = -538.801314090 a.u.
ν_{min} = 35.2 cm^{-1}

C	3.058339	2.612569	-1.445380	H	0.965918	3.138687	-1.275835
H	4.003257	2.802969	-0.930539	C	1.709724	2.798998	0.698568
H	2.886252	1.532759	-1.469661	C	2.676258	3.060036	1.681067
H	3.153564	2.973153	-2.474538	C	0.585420	2.039337	1.041909
C	2.120782	4.818603	-0.680750	C	2.521650	2.567645	2.977202
O	3.206765	5.365198	-0.676492	H	3.546896	3.658551	1.428157
O	0.952776	5.482735	-0.600534	C	0.430580	1.543024	2.338497
C	1.056965	6.915497	-0.467987	H	-0.171774	1.834059	0.289534
H	0.031789	7.282145	-0.412580	C	1.398415	1.805514	3.310020
H	1.603624	7.178806	0.441590	H	3.277751	2.780160	3.727885
H	1.568138	7.347649	-1.332349	H	-0.447230	0.953565	2.588407
C	1.896331	3.312350	-0.727138	H	1.278242	1.421552	4.319033

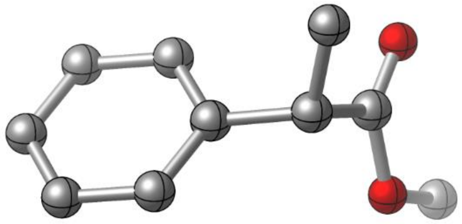

Zero-point correction= 0.200313 (Hartree/Particle)

Thermal correction to Energy= 0.211882
Thermal correction to Enthalpy= 0.212826
Thermal correction to Gibbs Free Energy= 0.161657
Sum of electronic and zero-point Energies= -538.601001
Sum of electronic and thermal Energies= -538.589432
Sum of electronic and thermal Enthalpies= -538.588488
Sum of electronic and thermal Free Energies= -538.639657

THF
E(scf) = -232.473470188 a.u.
ν_{min} = 65.0 cm^{-1}

C	-0.547406	2.930858	-1.213244	H	2.496088	2.716303	-0.347258
O	0.712038	2.241405	-1.285503	H	2.312015	3.372542	-1.988968
C	1.782329	3.170532	-1.044899	H	0.988993	4.366992	0.584026
C	1.133495	4.445474	-0.499731	H	1.727171	5.340954	-0.703321
C	-0.222383	4.426888	-1.219330	H	-0.991131	5.027313	-0.725052
H	-1.170790	2.620666	-2.060415	H	-0.111005	4.789117	-2.247886
H	-1.068615	2.646083	-0.285981				

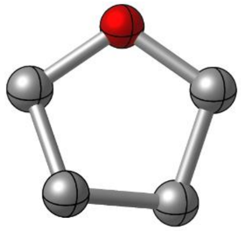

Zero-point correction= 0.116755 (Hartree/Particle)
Thermal correction to Energy= 0.121651
Thermal correction to Enthalpy= 0.122595
Thermal correction to Gibbs Free Energy= 0.088549
Sum of electronic and zero-point Energies= -232.356715
Sum of electronic and thermal Energies= -232.351819
Sum of electronic and thermal Enthalpies= -232.350875
Sum of electronic and thermal Free Energies= -232.384921

^{2}TS8'
E(scf) = -3343.43392962 a.u.
ν_{min} = -299.9 cm^{-1}

C	-2.085339	-1.021640	-0.338472	H	-5.334072	-1.473328	-2.445460
C	-2.723995	-2.268421	-0.419464	H	-2.320811	-3.117271	0.121472
C	-2.605200	0.070491	-1.081397	H	-4.220357	0.708484	-2.363992
C	-3.789245	-0.116399	-1.812125	P	-0.615090	-0.757186	0.759002
C	-3.877951	-2.443164	-1.181553	P	-1.617323	1.644546	-1.169252
H	-4.350595	-3.419674	-1.233644	Fe	0.529105	1.078983	-0.290182
C	-4.424599	-1.356451	-1.863254	C	0.310517	-2.349307	0.771395

C	-1.380708	-0.738342	2.509587
C	-1.832470	2.425562	-2.909589
C	-2.591093	2.819472	-0.124024
C	1.668618	1.742862	1.268913
C	2.915851	1.071086	1.274778
C	1.217292	2.238634	2.514456
C	3.622376	0.848019	2.455066
H	3.336811	0.726206	0.339968
C	1.935855	2.037643	3.690262
H	0.290523	2.799363	2.565808
C	3.141188	1.328013	3.677050
H	4.565117	0.307518	2.415014
H	1.545095	2.430074	4.626001
H	3.700518	1.168993	4.594166
C	1.338491	2.915699	-0.160769
C	2.422913	3.102285	-1.052766
C	0.604711	4.074910	0.192946
C	2.704298	4.351790	-1.604810
H	3.036869	2.253770	-1.330499
C	0.898036	5.322707	-0.345870
H	-0.215075	3.994021	0.897953
C	1.947386	5.474969	-1.260388
H	3.531522	4.447481	-2.304490
H	0.299586	6.183345	-0.055944
H	2.177804	6.449379	-1.680990
H	-3.663440	2.733693	-0.317417
H	-2.267301	3.840033	-0.338289
H	-2.404759	2.617726	0.929785
C	-0.728671	3.500072	-2.996953
H	-0.771977	3.980064	-3.982320
H	-0.853210	4.279671	-2.242153
H	0.268696	3.072389	-2.871205
C	-3.189364	3.121075	-3.137700
H	-3.177259	3.589314	-4.129866

H	-4.039018	2.434765	-3.120298
H	-3.379021	3.914424	-2.410491
C	-1.592020	1.391552	-4.020502
H	-1.702939	1.885059	-4.993825
H	-0.581176	0.982508	-3.976112
H	-2.303558	0.562060	-3.987021
H	1.102427	-2.249449	1.518133
H	-0.305906	-3.215114	1.025752
H	0.805726	-2.500241	-0.186623
C	-0.221037	-0.850782	3.518866
H	-0.586042	-0.591367	4.520304
H	0.169292	-1.871487	3.568863
H	0.605846	-0.179357	3.283357
C	-2.115553	0.597880	2.683455
H	-1.437739	1.447012	2.569960
H	-2.928978	0.698734	1.958280
H	-2.555821	0.653199	3.686590
C	-2.371708	-1.885609	2.766220
H	-2.689771	-1.849621	3.816088
H	-3.268577	-1.803609	2.146924
H	-1.924324	-2.869824	2.596571
C	1.274679	-0.077016	-1.994855
C	0.350216	-1.129572	-2.626828
H	0.326663	-2.048709	-2.040119
H	-0.678853	-0.788788	-2.716599
H	0.688903	-1.410675	-3.636209
C	2.576235	-0.678067	-1.696022
O	2.794316	-1.815285	-1.285369
O	3.635916	0.179191	-1.926271
C	4.933777	-0.340203	-1.608212
H	5.637281	0.468999	-1.812553
H	5.002627	-0.636076	-0.556721
H	5.17205	-1.209052	-2.22917
H	1.424057	0.75931	-2.688662

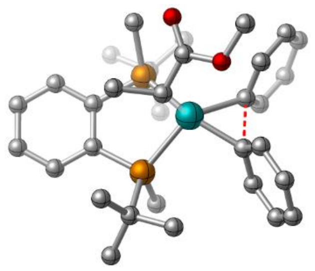

Zero-point correction= 0.695400 (Hartree/Particle)
Thermal correction to Energy= 0.737127
Thermal correction to Enthalpy= 0.738071
Thermal correction to Gibbs Free Energy= 0.624336
Sum of electronic and zero-point Energies= -3342.738530
Sum of electronic and thermal Energies= -3342.696803
Sum of electronic and thermal Enthalpies= -3342.695859
Sum of electronic and thermal Free Energies= -3342.809593

^{4}TS7

E(scf) = -3343.46003614 a.u.

ν_{min} = -138.4 cm^{-1}

C	0.003439	4.214989	-2.406891	H	-4.080150	2.833457	-0.963350
H	-0.376870	4.253480	-1.383603	H	-2.609823	3.354162	-0.111883
H	0.243687	5.249239	-2.705687	C	1.613347	2.811651	-3.785845
H	-0.782924	3.862867	-3.077865	O	0.893258	2.716407	-4.778074
C	-2.213737	-0.877004	0.637390	O	2.922474	2.441982	-3.793167
C	-2.866169	-1.777207	1.496046	C	3.400446	1.875648	-5.020046
C	-2.965883	0.171601	0.050178	H	4.405714	1.509292	-4.807439
C	-4.316838	0.322592	0.407165	H	2.763627	1.051459	-5.344974
C	-4.212213	-1.625925	1.822337	H	3.438557	2.634443	-5.809426
H	-4.688739	-2.333555	2.494472	C	1.215116	3.352863	-2.500778
C	-4.936076	-0.558297	1.291284	H	1.990170	3.456528	-1.753169
H	-5.981164	-0.418347	1.551742	C	0.796061	2.291069	0.518705
H	-2.318428	-2.608286	1.922192	C	2.143987	2.644973	0.751985
H	-4.902083	1.132131	-0.014077	C	-0.136460	2.836379	1.426133
P	-0.390559	-0.995833	0.313494	C	2.537580	3.481488	1.801645
P	-2.191378	1.240094	-1.249202	H	2.918948	2.253071	0.092750
Fe	0.314437	0.971279	-1.006092	C	0.234061	3.683441	2.478503
C	-0.229821	-2.582098	-0.611981	H	-1.189327	2.575697	1.334676
H	0.830419	-2.824430	-0.719308	C	1.578207	4.010611	2.670025
H	-0.745551	-3.403640	-0.107974	H	3.589252	3.723033	1.942667
H	-0.649613	-2.459496	-1.610789	H	-0.523032	4.080258	3.152057
C	0.486573	-1.359224	1.968921	H	1.875011	4.665000	3.485811
C	-2.927148	0.604727	-2.880192	C	1.497633	-0.078184	-2.325231
C	-2.990952	2.886698	-1.021757	C	2.764686	-0.464570	-1.834399
H	-2.720512	3.521207	-1.866924	C	1.170591	-0.559031	-3.608309

C	3.639714	-1.280007	-2.559052
H	3.084396	-0.121148	-0.851791
C	2.025467	-1.390956	-4.342712
H	0.228874	-0.272590	-4.068583
C	3.267022	-1.757412	-3.819256
H	4.607757	-1.548790	-2.140553
H	1.724915	-1.744927	-5.327016
H	3.936060	-2.400798	-4.385280
C	-0.059877	-0.419548	3.055887
H	0.002992	0.628878	2.753928
H	-1.101516	-0.644195	3.303825
H	0.534994	-0.539814	3.969624
C	1.971703	-1.035664	1.705980
H	2.553203	-1.233731	2.614667
H	2.385822	-1.655721	0.904379
H	2.110879	0.013823	1.436602
C	0.392139	-2.820893	2.442866
H	0.975156	-2.926442	3.366324
H	-0.628533	-3.133918	2.673946
H	0.809615	-3.520910	1.713860
C	-2.506021	-0.865625	-3.030078
H	-2.832177	-1.244944	-4.005918
H	-1.421730	-0.978997	-2.974956
H	-2.957826	-1.496024	-2.257845
C	-4.457387	0.707151	-2.957529
H	-4.790922	0.344881	-3.938135
H	-4.947811	0.092810	-2.197331
H	-4.811559	1.737296	-2.854244
C	-2.29405	1.454729	-3.999353
H	-2.574692	1.038802	-4.974831
H	-2.653831	2.488498	-3.973866
H	-1.202017	1.483215	-3.950092

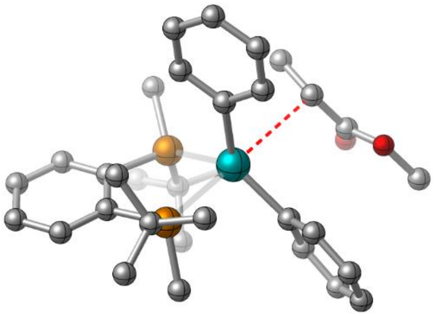

Zero-point correction= 0.692523 (Hartree/Particle)
Thermal correction to Energy= 0.735668
Thermal correction to Enthalpy= 0.736612
Thermal correction to Gibbs Free Energy= 0.616623
Sum of electronic and zero-point Energies= -3342.767513
Sum of electronic and thermal Energies= -3342.724368
Sum of electronic and thermal Enthalpies= -3342.723424
Sum of electronic and thermal Free Energies= -3342.843413

References

1. *CrysAlisPro*, version 171.39.46; Rigaku Corporation: Oxford, UK, 2018.

2. Sheldrick, G. M. *SHELXT*, version 2018/2; *Acta. Crystallogr.* **2015**, *A71*, 3-8.

3. Sheldrick, G. M. *SHELXL*, version 2018/3; *Acta. Crystallogr.* **2015**, *C71*, 3-8.

4. Dolomanov, O. V.; Bourhis, L. J.; Gildea, R. J.; Howard, J. A. K.; Puschmann, H. *Olex2*, version 1.2-ac3; *J. Appl. Cryst.* **2009**, *42*, 339-341.

5. (a) Lee, C.; Yang, W.; Parr, R. G., Development of the Colle-Salvetti Correlation-Energy Formula into a Functional of the Electron Density. *Phys. Rev. B* **1988**, *37*, 785–789. (b) Becke, A. D., Density-Functional Thermochemistry. III. The Role of Exact Exchange. *J. Chem. Phys.* **1993**, *98*, 5648–5652.

6. (a) Grimme, S. Accurate description of van der Waals complexes by density functional theory including empirical corrections. *J. Comput. Chem.* **2004**, *25*, 1463-1473. (b) Grimme, S.; Antony, J.; Ehrlich, S.; Krieg, H. A consistent and accurate ab initio parametrization of density functional dispersion correction (DFT-D) for the 94 elements H-Pu. *J. Chem. Phys.* **2010**, *132*, 154104. (c) Grimme, S. Density functional theory with London dispersion corrections. *WIRES Comput. Mol. Sci.* **2011**, *1*, 211-228. (d) Ehrlich, S.; Moellmann, J.; Grimme, S. Dispersion-Corrected Density Functional Theory for Aromatic Interactions in Complex Systems. *Acc. Chem. Res.* **2012**, *46*, 916-926.

7. (a) Petersson, G. A.; Tensfeldt, T. G.; Montgomery, J. A., Jr. A Complete Basis Set Model Chemistry. III. The Complete Basis Set-quadratic Configuration Interaction Family of Methods. *J. Chem. Phys.* **1991**, *94*, 6091–6101. (b) Petersson, G. A.; Bennett, A.; Tensfeldt, T. G.; Al-Laham, M. A.; Shirley, W. A.; Mantzaris, J. A Complete Basis Set Model Chemistry. I. The Total Energies of Closed-shell Atoms and Hydrides of the First-row Elements. *J. Chem. Phys.* **1988**, *89*, 2193–2218.

8. Marenich, A. V.; Cramer, C. J.; Truhlar, D. G. Universal Solvation Model Based on Solute Electron Density and on a Continuum Model of the Solvent Defined by the Bulk Dielectric Constant and Atomic Surface Tensions. *J. Phys. Chem. B* **2009**, *113*, 6378–6396.

9. Gaussian 16, Revision C.01, Frisch, M. J.; Trucks, G. W.; Schlegel, H. B.; Scuseria, G. E.; Robb, M. A.; Cheeseman, J. R.; Scalmani, G.; Barone, V.; Petersson, G. A.; Nakatsuji, H.; Li, X.; Caricato, M.; Marenich, A. V.; Bloino, J.; Janesko, B. G.; Gomperts, R.; Mennucci, B.; Hratchian, H. P.; Ortiz, J. V.; Izmaylov, A. F.; Sonnenberg, J. L.; Williams-Young, D.; Ding, F.; Lipparini, F.; Egidi, F.; Goings, J.; Peng, B.; Petrone, A.; Henderson, T.; Ranasinghe, D.; Zakrzewski, V. G.; Gao, J.; Rega, N.; Zheng, G.; Liang, W.; Hada, M.; Ehara, M.; Toyota, K.; Fukuda, R.; Hasegawa, J.; Ishida, M.; Nakajima, T.; Honda, Y.; Kitao, O.; Nakai, H.; Vreven, T.; Throssell, K.; Montgomery, J. A., Jr.; Peralta, J. E.; Ogliaro, F.; Bearpark, M. J.; Heyd, J. J.; Brothers, E. N.; Kudin, K. N.; Staroverov, V. N.; Keith, T. A.; Kobayashi, R.; Normand, J.; Raghavachari, K.; Rendell, A. P.; Burant, J. C.; Iyengar, S. S.; Tomasi, J.; Cossi, M.; Millam, J. M.; Klene, M.; Adamo, C.; Cammi, R.; Ochterski, J. W.; Martin, R. L.; Morokuma, K.; Farkas, O.; Foresman, J. B.; Fox, D. J. Gaussian, Inc., Wallingford CT, 2016.

10. Legault, C. Y. (2009) CYLview, 1.0b, Université de Sherbrooke: Sherbrooke, Canada, http://www.cylview.org.
