# Supplementary material for: Insight into Radical Initiation, Solvent Effects, and Biphenyl Production in Iron–Bisphosphine Cross-Couplings
Source: ACS Catal. 2023 Jun 22;13(13):8987–96. doi: 10.1021/acscatal.3c02008 (PMC10334425; doi:10.1021/acscatal.3c02008)

checkCIF/PLATON report

Structure factors have been supplied for datablock(s) neimca04

THIS REPORT IS FOR GUIDANCE ONLY. IF USED AS PART OF A REVIEW PROCEDURE FOR PUBLICATION, IT SHOULD NOT REPLACE THE EXPERTISE OF AN EXPERIENCED CRYSTALLOGRAPHIC REFEREE.

No syntax errors found. CIF dictionary Interpreting this report

Datablock: neimca04

Bond precision: C-C = 0.0095 A Wavelength=1.54184

Cell:	a=9.89150(12)	b=17.8509(2)	c=26.9080(4)
	alpha=90	beta=90	gamma=90
Temperature:	100 K		

	Calculated	Reported
Volume	4751.21(11)	4751.21(11)
Space group	P 21 21 21	P 21 21 21
Hall group	P 2ac 2ab	P 2ac 2ab
Moiety formula	C20.96 H32.13 Br1.17 Fe P2, C21.73 H32.78 Br1.05 Fe P2	0.5(C21.729 H32.774 Br1.045 Fe P2), 0.5(C20.954 H32.128 Br1.174
Sum formula	C42.69 H64.91 Br2.22 Fe2 P4	C21.34 H32.45 Br1.11 Fe P2
Mr	990.96	495.51
Dx,g cm-3	1.385	1.385
Z	4	8
Mu (mm-1)	8.552	8.553
F000	2042.7	2043.0
F000'	2035.70	
h,k,lmax	12,22,34	12,22,34
Nref	10146[5638]	9952
Tmin,Tmax	0.512,0.569	0.432,1.000
Tmin'	0.365	

Correction method= # Reported T Limits: Tmin=0.432 Tmax=1.000
AbsCorr = MULTI-SCAN

Data completeness= 1.77/0.98 Theta(max)= 77.968

R(reflections)= 0.0439(9205)   wR2(reflections)= 0.1143(9952)

S = 1.052   Npar= 507

The following ALERTS were generated. Each ALERT has the format
test-name_ALERT_alert-type_alert-level.
Click on the hyperlinks for more details of the test.

Alert level C

```
PLAT042_ALERT_1_C Calc. and Reported MoietyFormula Strings  Differ     Please Check
PLAT077_ALERT_4_C Unitcell Contains Non-integer Number of Atoms ..     Please Check
PLAT094_ALERT_2_C Ratio of Maximum / Minimum Residual Density ....       2.15 Report
PLAT341_ALERT_3_C Low Bond Precision on  C-C Bonds ..............     0.0095 Ang.
```

Alert level G

```
PLAT002_ALERT_2_G Number of Distance or Angle Restraints on AtSite         15 Note
PLAT003_ALERT_2_G Number of Uiso or Uij Restrained non-H Atoms ...          7 Report
PLAT045_ALERT_1_G Calculated and Reported Z Differ by a Factor ...      0.500 Check
PLAT068_ALERT_1_G Reported F000 Differs from Calcd (or Missing)...     Please Check
PLAT171_ALERT_4_G The CIF-Embedded .res File Contains EADP Records          3 Report
PLAT175_ALERT_4_G The CIF-Embedded .res File Contains SAME Records          2 Report
PLAT176_ALERT_4_G The CIF-Embedded .res File Contains SADI Records          2 Report
PLAT178_ALERT_4_G The CIF-Embedded .res File Contains SIMU Records          3 Report
PLAT188_ALERT_3_G A Non-default SIMU Restraint Value has been used     0.0100 Report
PLAT188_ALERT_3_G A Non-default SIMU Restraint Value has been used     0.0100 Report
PLAT188_ALERT_3_G A Non-default SIMU Restraint Value has been used     0.0050 Report
PLAT189_ALERT_3_G A Non-default SAME Restraint Value for SecondPar     0.0400 Report
PLAT189_ALERT_3_G A Non-default SAME Restraint Value for SecondPar     0.0400 Report
PLAT191_ALERT_3_G A Non-default SADI Restraint Value has been used     0.0100 Report
PLAT191_ALERT_3_G A Non-default SADI Restraint Value has been used     0.0100 Report
PLAT232_ALERT_2_G Hirshfeld Test Diff (M-X)  Br2      --Fe2      .        6.4 s.u.
PLAT301_ALERT_3_G Main Residue  Disorder ..............(Resd  1  )        24% Note
```

Author Response: Both independent molecules are modeled as disorders of two distinct species, (PP)FeBrPh and (PP)FeBr~2~, with disorder ratios of 0.955(2):0.045(2) and 0.826(3):0.174(3), respectively, for molecules containing Fe1 and Fe2. The bromido ligand set of the minor component of disorder for molecule Fe2 is additionally modeled as disordered over two positions (0.903(2):0.097(2)).

```
PLAT301_ALERT_3_G Main Residue  Disorder ..............(Resd  2  )        22% Note
```

Author Response: Both independent molecules are modeled as disorders of two distinct species, (PP)FeBrPh and (PP)FeBr~2~, with disorder ratios of 0.955(2):0.045(2) and 0.826(3):0.174(3), respectively, for molecules containing Fe1 and Fe2. The bromido ligand set of the minor component of disorder for molecule Fe2 is additionally modeled as disordered over two positions (0.903(2):0.097(2)).

```
PLAT304_ALERT_4_G Non-Integer Number of Atoms in ..... (Resd  1  )      57.26 Check
PLAT304_ALERT_4_G Non-Integer Number of Atoms in ..... (Resd  2  )      58.55 Check
PLAT791_ALERT_4_G Model has Chirality at P1          (Sohnke SpGr)          S Verify
PLAT791_ALERT_4_G Model has Chirality at P2          (Sohnke SpGr)          S Verify
PLAT791_ALERT_4_G Model has Chirality at P3          (Sohnke SpGr)          S Verify
PLAT791_ALERT_4_G Model has Chirality at P4          (Sohnke SpGr)          S Verify
PLAT860_ALERT_3_G Number of Least-Squares Restraints ............         57 Note
```

Author Response: Analogous bond lengths and angles between positions of disorder were restrained to be similar. Anisotropic displacement parameters for proximal atoms were constrained to be equivalent and/or restrained to be similar.

```
PLAT912_ALERT_4_G Missing # of FCF Reflections Above STh/L=  0.600         51 Note
PLAT978_ALERT_2_G Number C-C Bonds with Positive Residual Density.          0 Info
```

```
   0 ALERT level A = Most likely a serious problem - resolve or explain
   0 ALERT level B = A potentially serious problem, consider carefully
   4 ALERT level C = Check. Ensure it is not caused by an omission or oversight
  27 ALERT level G = General information/check it is not something unexpected

   3 ALERT type 1 CIF construction/syntax error, inconsistent or missing data
   5 ALERT type 2 Indicator that the structure model may be wrong or deficient
  11 ALERT type 3 Indicator that the structure quality may be low
  12 ALERT type 4 Improvement, methodology, query or suggestion
   0 ALERT type 5 Informative message, check
```

It is advisable to attempt to resolve as many as possible of the alerts in all categories. Often the minor alerts point to easily fixed oversights, errors and omissions in your CIF or refinement strategy, so attention to these fine details can be worthwhile. In order to resolve some of the more serious problems it may be necessary to carry out additional measurements or structure refinements. However, the purpose of your study may justify the reported deviations and the more serious of these should normally be commented upon in the discussion or experimental section of a paper or in the "special_details" fields of the CIF. checkCIF was carefully designed to identify outliers and unusual parameters, but every test has its limitations and alerts that are not important in a particular case may appear. Conversely, the absence of alerts does not guarantee there are no aspects of the results needing attention. It is up to the individual to critically assess their own results and, if necessary, seek expert advice.

Publication of your CIF in IUCr journals

A basic structural check has been run on your CIF. These basic checks will be run on all CIFs submitted for publication in IUCr journals (*Acta Crystallographica*, *Journal of Applied Crystallography*, *Journal of Synchrotron Radiation*); however, if you intend to submit to *Acta Crystallographica Section C* or *E* or *IUCrData*, you should make sure that full publication checks are run on the final version of your CIF prior to submission.

Publication of your CIF in other journals

Please refer to the *Notes for Authors* of the relevant journal for any special instructions relating to CIF submission.

PLATON version of 28/11/2022; check.def file version of 28/11/2022

Datablock neimca04 - ellipsoid plot

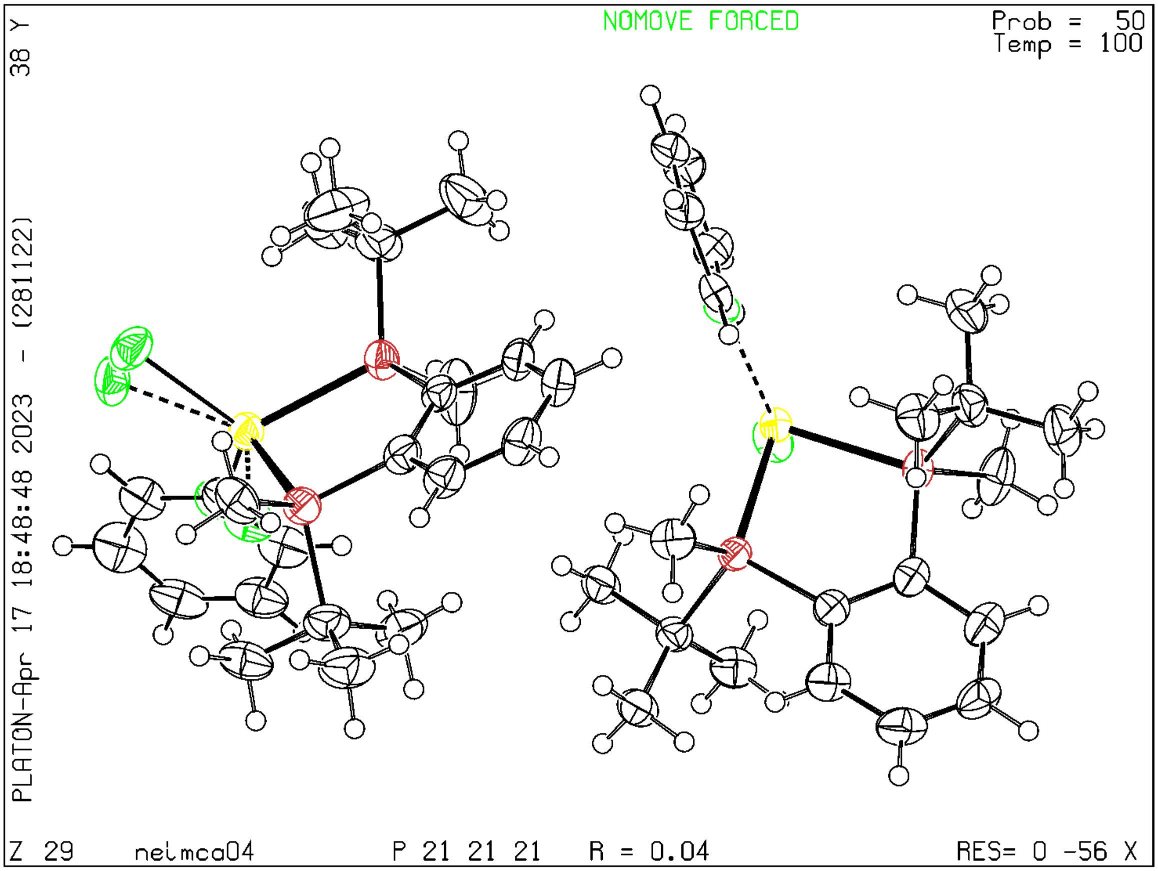

Supplement: Supplementary file 3 — cs3c02008_si_003.pdf [file cs3c02008_si_003.pdf]
