# Supplementary material for: Insight into Radical Initiation, Solvent Effects, and Biphenyl Production in Iron–Bisphosphine Cross-Couplings
Source: ACS Catal. 2023 Jun 22;13(13):8987–96. doi: 10.1021/acscatal.3c02008 (PMC10334425; doi:10.1021/acscatal.3c02008)

# checkCIF/PLATON report

Structure factors have been supplied for datablock(s) neimca01mo

THIS REPORT IS FOR GUIDANCE ONLY. IF USED AS PART OF A REVIEW PROCEDURE FOR PUBLICATION, IT SHOULD NOT REPLACE THE EXPERTISE OF AN EXPERIENCED CRYSTALLOGRAPHIC REFEREE.

No syntax errors found. CIF dictionary Interpreting this report

# Datablock: neimca01mo

---

| | | | |
|---|---|---|---|
| Bond precision: | C-C = 0.0112 A | | Wavelength=0.71073 |
| Cell: | a=9.6327(2) | b=11.1804(4) | c=22.2889(6) |
| | alpha=90 | beta=90 | gamma=90 |
| Temperature: | 100 K | | |

| | Calculated | Reported |
|---|---|---|
| Volume | 2400.46(12) | 2400.46(12) |
| Space group | P 21 21 21 | P 21 21 21 |
| Hall group | P 2ac 2ab | P 2ac 2ab |
| Moiety formula | 2(C16 H28 Br2 Fe P2), C2 H4 O, 2(C H3) | C16 H28 Br2 Fe P2, 0.5(C4 H10 O) |
| Sum formula | C36 H66 Br4 Fe2 O P4 | C18 H33 Br2 Fe O0.50 P2 |
| Mr | 1070.07 | 535.05 |
| Dx,g cm-3 | 1.480 | 1.481 |
| Z | 2 | 4 |
| Mu (mm-1) | 4.092 | 4.092 |
| F000 | 1084.0 | 1084.0 |
| F000' | 1084.24 | |
| h,k,lmax | 13,15,30 | 13,15,30 |
| Nref | 6462[ 3639] | 6459 |
| Tmin,Tmax | 0.566,0.640 | 0.628,1.000 |
| Tmin' | 0.277 | |

Correction method= # Reported T Limits: Tmin=0.628 Tmax=1.000
AbsCorr = MULTI-SCAN

Data completeness= 1.77/1.00 Theta(max)= 29.129

R(reflections)= 0.0499( 5859) wR2(reflections)= 0.1038( 6459)

S = 1.234 Npar= 238

The following ALERTS were generated. Each ALERT has the format
**test-name_ALERT_alert-type_alert-level.**
Click on the hyperlinks for more details of the test.

## Alert level C

```
PLAT042_ALERT_1_C Calc. and Reported MoietyFormula Strings  Differ     Please Check
PLAT341_ALERT_3_C Low Bond Precision on  C-C Bonds ..............    0.01125 Ang.
PLAT906_ALERT_3_C Large K Value in the Analysis of Variance ......      2.679 Check
PLAT934_ALERT_3_C Number of (Iobs-Icalc)/Sigma(W) > 10 Outliers ..          1 Check
```

## Alert level G

```
PLAT002_ALERT_2_G Number of Distance or Angle Restraints on AtSite          5 Note
PLAT003_ALERT_2_G Number of Uiso or Uij Restrained non-H Atoms ...          5 Report
PLAT045_ALERT_1_G Calculated and Reported Z Differ by a Factor ...      0.500 Check
PLAT083_ALERT_2_G SHELXL Second Parameter in WGHT  Unusually Large      10.15 Why ?
PLAT171_ALERT_4_G The CIF-Embedded .res File Contains EADP Records          1 Report
PLAT175_ALERT_4_G The CIF-Embedded .res File Contains SAME Records          1 Report
PLAT178_ALERT_4_G The CIF-Embedded .res File Contains SIMU Records          1 Report
PLAT188_ALERT_3_G A Non-default SIMU Restraint Value has been used     0.0100 Report
PLAT189_ALERT_3_G A Non-default SAME Restraint Value for SecondPar     0.0400 Report
PLAT300_ALERT_4_G Atom Site Occupancy of O1         Constrained at        0.5 Check
PLAT300_ALERT_4_G Atom Site Occupancy of C18        Constrained at        0.5 Check
PLAT300_ALERT_4_G Atom Site Occupancy of C19        Constrained at        0.5 Check
PLAT300_ALERT_4_G Atom Site Occupancy of H18A       Constrained at        0.5 Check
PLAT300_ALERT_4_G Atom Site Occupancy of H18B       Constrained at        0.5 Check
PLAT300_ALERT_4_G Atom Site Occupancy of H19A       Constrained at        0.5 Check
PLAT300_ALERT_4_G Atom Site Occupancy of H19B       Constrained at        0.5 Check
PLAT300_ALERT_4_G Atom Site Occupancy of C17        Constrained at        0.5 Check
PLAT300_ALERT_4_G Atom Site Occupancy of H17A       Constrained at        0.5 Check
PLAT300_ALERT_4_G Atom Site Occupancy of H17B       Constrained at        0.5 Check
PLAT300_ALERT_4_G Atom Site Occupancy of H17C       Constrained at        0.5 Check
PLAT300_ALERT_4_G Atom Site Occupancy of C20        Constrained at        0.5 Check
PLAT300_ALERT_4_G Atom Site Occupancy of H20A       Constrained at        0.5 Check
PLAT300_ALERT_4_G Atom Site Occupancy of H20B       Constrained at        0.5 Check
PLAT300_ALERT_4_G Atom Site Occupancy of H20C       Constrained at        0.5 Check
PLAT302_ALERT_4_G Anion/Solvent/Minor-Residue Disorder (Resd  2  )       100% Note
```

**Author Response: The cocrystallized diethyl ether molecule is disordered in channels along [100]. It was modeled as disordered over a crystallographic screw axis.**

```
PLAT302_ALERT_4_G Anion/Solvent/Minor-Residue Disorder (Resd  3  )       100% Note
```

**Author Response: The cocrystallized diethyl ether molecule is disordered in channels along [100]. It was modeled as disordered over a crystallographic screw axis.**

```
PLAT302_ALERT_4_G Anion/Solvent/Minor-Residue Disorder (Resd  4  )       100% Note
```

**Author Response: The cocrystallized diethyl ether molecule is disordered in channels along [100]. It was modeled as disordered over a crystallographic screw axis.**

```
PLAT304_ALERT_4_G Non-Integer Number of Atoms in ..... (Resd  2  )       3.50 Check
PLAT398_ALERT_2_G Deviating  C-O-C   Angle From 120 for O1       .      109.3 Degree
PLAT773_ALERT_2_G Check long C-C Bond in CIF: C17      --C18             1.78 Ang.
PLAT773_ALERT_2_G Check long C-C Bond in CIF: C19      --C20             1.79 Ang.
PLAT789_ALERT_4_G Atoms with Negative _atom_site_disorder_group  #         15 Check
PLAT791_ALERT_4_G Model has Chirality at P1          (Sohnke SpGr)          S Verify
PLAT791_ALERT_4_G Model has Chirality at P2          (Sohnke SpGr)          S Verify
PLAT860_ALERT_3_G Number of Least-Squares Restraints ............         27 Note
```

**Author Response: Analogous bond lengths and angles along the disordered diethyl ether molecule were restrained to be similar. Anisotropic displacement parameters for proximal atoms, including those due to symmetry, were restrained to be similar and/or constrained to be equivalent.**

```
PLAT910_ALERT_3_G Missing # of FCF Reflection(s) Below Theta(Min).          2 Note
PLAT912_ALERT_4_G Missing # of FCF Reflections Above STh/L=  0.600          1 Note
PLAT933_ALERT_2_G Number of HKL-OMIT Records in Embedded .res File          2 Note
PLAT978_ALERT_2_G Number C-C Bonds with Positive Residual Density.          0 Info
```

---

```
   0 ALERT level A = Most likely a serious problem - resolve or explain
   0 ALERT level B = A potentially serious problem, consider carefully
   4 ALERT level C = Check. Ensure it is not caused by an omission or oversight
  39 ALERT level G = General information/check it is not something unexpected

   2 ALERT type 1 CIF construction/syntax error, inconsistent or missing data
   8 ALERT type 2 Indicator that the structure model may be wrong or deficient
   7 ALERT type 3 Indicator that the structure quality may be low
  26 ALERT type 4 Improvement, methodology, query or suggestion
   0 ALERT type 5 Informative message, check
```

---

---

It is advisable to attempt to resolve as many as possible of the alerts in all categories. Often the minor alerts point to easily fixed oversights, errors and omissions in your CIF or refinement strategy, so attention to these fine details can be worthwhile. In order to resolve some of the more serious problems it may be necessary to carry out additional measurements or structure refinements. However, the purpose of your study may justify the reported deviations and the more serious of these should normally be commented upon in the discussion or experimental section of a paper or in the "special_details" fields of the CIF. checkCIF was carefully designed to identify outliers and unusual parameters, but every test has its limitations and alerts that are not important in a particular case may appear. Conversely, the absence of alerts does not guarantee there are no aspects of the results needing attention. It is up to the individual to critically assess their own results and, if necessary, seek expert advice.

**Publication of your CIF in IUCr journals**

A basic structural check has been run on your CIF. These basic checks will be run on all CIFs submitted for publication in IUCr journals (*Acta Crystallographica*, *Journal of Applied Crystallography*, *Journal of Synchrotron Radiation*); however, if you intend to submit to *Acta Crystallographica Section C* or *E* or *IUCrData*, you should make sure that full publication checks are run on the final version of your CIF prior to submission.

**Publication of your CIF in other journals**

Please refer to the *Notes for Authors* of the relevant journal for any special instructions relating to CIF submission.

---

**PLATON version of 28/11/2022; check.def file version of 28/11/2022**

**Datablock neimca01mo** - ellipsoid plot

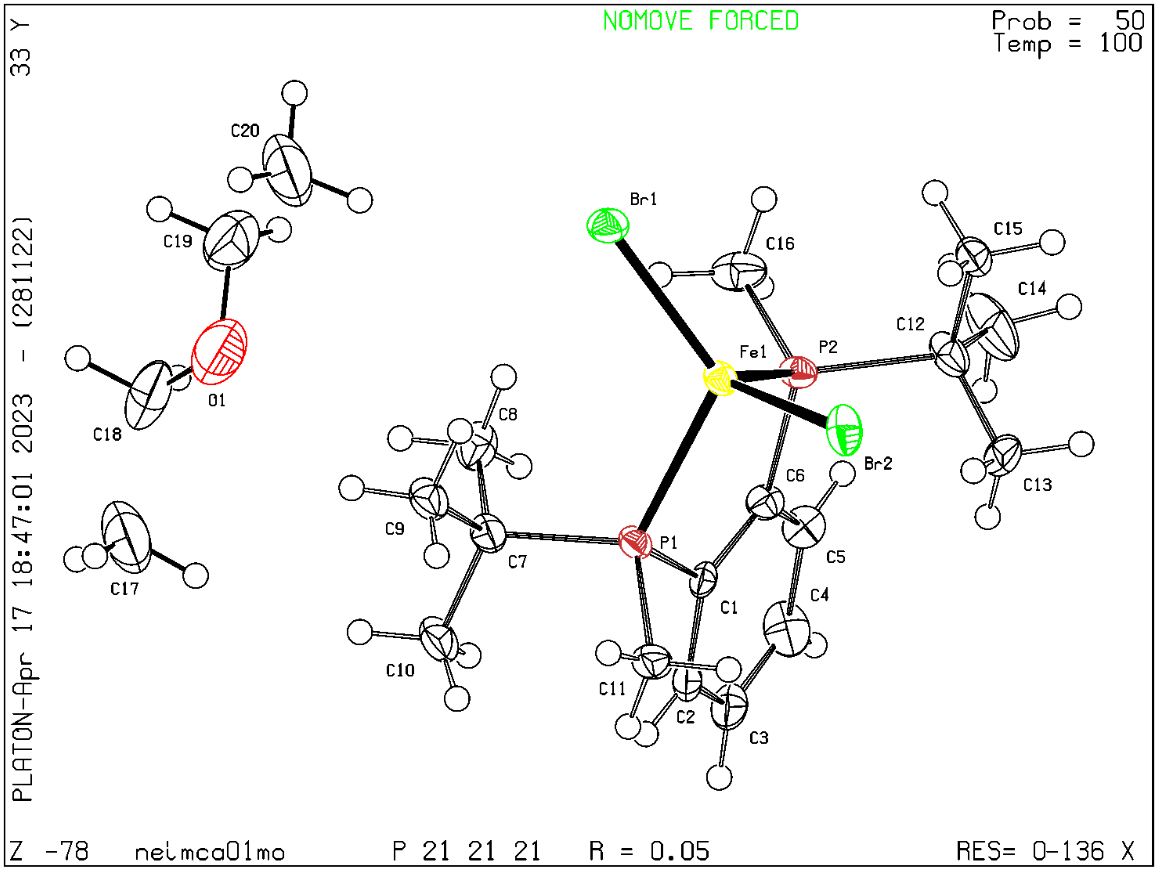

Supplement: Supplementary file 4 — cs3c02008_si_004.pdf [file cs3c02008_si_004.pdf]
